# Gene Duplication and Evolution Dynamics in the Homeologous Regions Harboring Multiple Prolamin and Resistance Gene Families in Hexaploid Wheat

**DOI:** 10.3389/fpls.2018.00673

**Published:** 2018-05-23

**Authors:** Naxin Huo, Shengli Zhang, Tingting Zhu, Lingli Dong, Yi Wang, Toni Mohr, Tiezhu Hu, Zhiyong Liu, Jan Dvorak, Ming-Cheng Luo, Daowen Wang, Jong-Yeol Lee, Susan Altenbach, Yong Q. Gu

**Affiliations:** ^1^United States Department of Agriculture-Agricultural Research Service, Western Regional Research Center, Albany, CA, United States; ^2^Department of Plant Sciences, University of California, Davis, Davis, CA, United States; ^3^Hena Institute of Science and Technology, Xinxiang, China; ^4^State Key Laboratory of Plant Cell and Chromosome Engineering, Institute of Genetics and Developmental Biology, Chinese Academy of Sciences, Beijing, China; ^5^National Institute of Agricultural Science, Rural Development Administration, Jeonju, South Korea

**Keywords:** wheat prolamins, disease resistance genes, gene family, gene duplication, genome evolution, phylogeny, *Aegilops tauschii*, *Triticum aestivum*

## Abstract

Improving end-use quality and disease resistance are important goals in wheat breeding. The genetic loci controlling these traits are highly complex, consisting of large families of prolamin and resistance genes with members present in all three homeologous A, B, and D genomes in hexaploid bread wheat. Here, orthologous regions harboring both prolamin and resistance gene loci were reconstructed and compared to understand gene duplication and evolution in different wheat genomes. Comparison of the two orthologous D regions from the hexaploid wheat Chinese Spring and the diploid progenitor *Aegilops tauschii* revealed their considerable difference due to the presence of five large structural variations with sizes ranging from 100 kb to 2 Mb. As a result, 44% of the *Ae. tauschii* and 71% of the Chinese Spring sequences in the analyzed regions, including 79 genes, are not shared. Gene rearrangement events, including differential gene duplication and deletion in the A, B, and D regions, have resulted in considerable erosion of gene collinearity in the analyzed regions, suggesting rapid evolution of prolamin and resistance gene families after the separation of the three wheat genomes. We hypothesize that this fast evolution is attributed to the co-evolution of the two gene families dispersed within a high recombination region. The identification of a full set of prolamin genes facilitated transcriptome profiling and revealed that the A genome contributes the least to prolamin expression because of its smaller number of expressed intact genes and their low expression levels, while the B and D genomes contribute similarly.

## Introduction

Gene duplication is one of the most important evolutionary processes that generate genetic diversity and functional novelty, and therefore plays an essential role in adaptation and speciation (Kondrashov, 2012; Magadum et al., 2013; Panchy et al., 2016). Plant genomes can accommodate large genomic redundancy and genetic diversity by retaining a high proportion of duplicate genes as compared to animal systems. On average, 65% of the annotated genes in plant genomes have a duplicate copy (Panchy et al., 2016). Although the mechanisms determining gene loss and retention after duplication are not well understood, retained gene copies often undergo further evolution through four trajectories (conservation, neofunctionalization, subfunctionalization, and specialization) (Freeling et al., 2015; Panchy et al., 2016).

Polyploidy is recognized as a major evolutionary force in plants (Soltis et al., 2015; Alix et al., 2017; Van de Peer et al., 2017). Bread wheat is an allohexaploid species (*Triticum aestivum* L. 2*n* = 6× = 42, genome AABBDD) generated by two rounds of allopolyploidization events (Matsuoka, 2011). In the first event occurring 0.36 to 0.5 million years ago, two diploid ancestors, *Triticum urartu* (2*n* = 2× = 14, genome AA) and an unconfirmed species related to *Aegilops speltoides* (2*n* = 2× = 14, genome SS), hybridized to form the cultivated allotetraploid emmer wheat (*Triticum turgidum* ssp. *dicoccum*, 2*n* = 4× = 28, genome AABB). The D genome was introduced when an allotetraploid wheat hybridized with an ancestral diploid *Aegilops tauschii* (DD) genome around 8000 to 10000 years ago. Hexaploid wheat therefore contains three related subgenomes, presumably with triplicate copies for each gene. Genetic evolution in polyploidy species is more complicated due to the interaction of multiple genomes in a single cell (Chen, 2007; Li et al., 2015). However, the genetic redundancy can buffer the rapid changes, thereby accelerating genome evolution. In nascent allopolyploid wheat, elimination of coding and non-coding DNA sequences, differential microRNA expressions, transposon activation, and gene silencing have been documented (Feldman and Levy, 2012; Li et al., 2015).

Hexaploid wheat speciation led to better adaptation to climates, enhanced yield potential, and increased economic value. The wheat prolamins confer the unique viscoelastic properties that provide value to wheat by facilitating the processing of flour into bread, pasta, noodles and other food products. The prolamins are composed of several complex protein groups that are all rich in glutamine and proline, but differ in size, domain structure, and biochemical properties (Shewry et al., 2002). The high molecular weight and low molecular weight glutenin subunits are linked together to form large polymers through intermolecular disulfide bonds and provide the elasticity to wheat dough, while the monomeric gliadins (subdivided into α, γ, δ, and ω-gliadins) confer extensibility. Unfortunately, prolamins are also known to be major triggers for celiac disease (CD), a food-sensitive autoimmune disorder that impacts 0.7∼2.0% of the human population, as well as other food allergies and sensitives (Sollid et al., 2012).

Genes encoding wheat prolamins are primarily mapped to three genomic regions (Shewry et al., 2003). The *Glu-1* loci encoding HMW-glutenin subunits (HMW-GS) are located on the long arms of group 1 chromosomes, while the *Gli-2* loci encoding α-gliadin genes are mapped to the short arm of group 6 chromosomes. The third genomic region on the short arm of group 1 chromosomes carries two tightly linked prolamin loci - *Glu-3* encoding LMW-glutenin subunits (LMW-GS) and *Gli-1* encoding γ-, δ-, and ω-gliadins. The prolamins account for ∼80% of endosperm proteins (Altenbach et al., 2011), largely attributed to the multiple gene copies in each prolamin locus and their high levels of expression in endosperm tissue. Elucidation of the genomic organizations of prolamin genes will help us better understand their evolution, expression, and the association of end-use quality with allelic variation (Bonafede et al., 2015). The genomic region carrying the *Glu-3* and *Gli-1* loci is particularly interesting since several resistance (*R*) loci are closely linked to the prolamin loci. These *R* loci have been shown to confer resistances to various pathogens including leaf, stem and stripe rust disease, powdery mildew, and Russian wheat aphid (Huang et al., 2003; Spielmeyer and Lagudah, 2003; Yahiaoui et al., 2004). Recent reports on the *Glu-3* and *Gli-1* genomic region from *Ae. tauschii*, the progenitor D genome donor of hexaploid wheat, revealed physical association of prolamin genes with nucleotide-binding domain and leucine-rich repeat (NLR) and receptor-like kinase (RLK) type resistance gene families and suggested that evolutionary dynamics in the region play important roles in gene duplication and expansion (Dong L. et al., 2016).

Our understanding of the genomic organization and evolution in the *Glu-3* and *Gli-1* regions in hexaploid wheat is still very limited. Most previous studies focused on identifying prolamin genes from different wheat species and cultivars. However, the tight linkage of the two loci and presence of multiple gene copies in each locus makes it very challenging to correlate allelic variations with the end-use properties of wheat flour. Recent advances in genome sequencing provide unprecedented opportunities to access genomic regions of interest quickly and economically. However, one major challenge in sequencing and characterizing genomic regions as complex as those carrying the prolamin genes is the presence of multiple tandemly duplicated gene copies and high repetitive DNA contents (Dong J. et al., 2016). Next generation sequencing methods with short reads often make it difficult to reconstruct these chromosomal regions. In addition, attempts to sequence the genomic regions from bacterial artificial chromosome (BAC) clones often provide limited information because the insert size of BAC clones often does not cover the entire prolamin region (Huang and Cloutier, 2008; Dong et al., 2010). PacBio single molecule real-time (SMRT) sequencing technology, which generates long sequence reads, has proved useful and effective in resolving complex genomic regions (Dong J. et al., 2016). Recently, all six maize prolamin gene regions from a maize inbred line were assembled from PacBio genome shotgun sequencing data (Dong J. et al., 2016). Three homeologous α-gliadin regions from hexaploid wheat cv Chinese Spring were also successfully reconstructed using large PacBio sequence contigs (Huo et al., 2018). In both studies, a restriction enzyme-based optical genome map was essential in validating and improving the assembly of these complex chromosomal regions.

In this study, we reconstructed the genomic regions carrying multiple prolamin and *R* gene loci from the A, B, and D genomes of hexaploid wheat cv Chinese Spring (CS) using PacBio sequence contigs and BioNano genome maps. We then conducted detailed sequence analysis to identify genes and pseudogenes for syntenic analyses among the CS A, B, and D genomes and performed phylogenetic analyses to understand rapid and independent evolutions of prolamin and *R* genes in different wheat genomes. The identification of a full complement of prolamin genes in a single genetic background facilitated our transcriptome analysis to understand the contribution of individual prolamin genes. This work represents the first report in which complex genomic regions controlling two important traits are characterized in detail in three homoeologous genomes of hexaploid Chinese Spring wheat.

## Materials and Methods

### *De Novo* BioNano Genome Map Assembly and Analysis

High molecular weight (HMW) DNA was isolated from young leaves (grown in darkness) of hexaploid wheat (*Triticum aestivum L.*) genotype ‘Chinese Spring’ by Amplicon Express (Pullman, WA, United States). The nicking endonuclease Nt.*Bsp*QI (New England BioLabs, Ipswich, MA, United States) was used to label high-quality HMW DNA molecules at specific sequence motifs (GCTCTTC) based on sequences of the publicly available hexaploid wheat genome (Clavijo et al., 2017). The nicked DNA molecules were stained according to the instructions of IrysPrep Reagent Kit (BioNano Genomics, San Diego, CA, United States), loaded onto the nanochannel array of IrysChip (BioNano Genomics), and automatically imaged by the Irys system (BioNano Genomics), as previously described in detail (Luo et al., 2017). Raw DNA molecules >20 kb were collected and converted into BNX files by AutoDetect software to obtain basic labeling and DNA length information. The filtered raw DNA molecules in BNX format were aligned, clustered, and assembled into the BioNano genome (BNG) map using the BioNano Genomics assembly pipeline as described in previous publications (Lam et al., 2012; Cao et al., 2014). The *P*-value thresholds used for pairwise assembly, extension/refinement, and merge stages were 1× 10^-10^, 1× 10^-11^, and 1× 10^-15^, respectively. The initial BNG map was then checked for potential chimeric BNG contigs and was further refined.

### Sequence Analysis and Gene Annotation

To reconstruct the genomic regions containing the prolamin and resistance gene loci, sequence contigs of Chinese Spring generated using PacBio read-only assembly and hybrid assembly of PacBio and Illumina reads (Zimin et al., 2017) were searched with BLAST using the sequences of all the 103 genes annotated in the *Ae. tauschii Glu-3* and *Gli-1* region (Dong L. et al., 2016). Sequence contigs with high stringent matches (*E*-value less than 1e^-100^) were downloaded. The extracted sequences were digested *in silico* according to the restriction site of Nt.*Bsp*QI by using Knickers (BioNano Genomics) and then aligned with the CS BNG map by computing with RefAligner (BioNano Genomics). The visualization of the alignment was performed with snapshot in IrysView (BioNano Genomics). Software packages used for these operations can be obtained from BioNano Genomics^1^. Manual check and editing are used to improve the final assembly by aligning, merging, and reorienting contigs (Hastie et al., 2013). The final assembled sequences for the A, B, and D genomes in this study were deposited in the NCBI GenBank under the accession numbers, MG560140, MG560141, and MG560142, respectively.

For sequence annotation, the final assembled genomic sequences for the A, B, and D genomes were first submitted to TriAnnot pipeline for automated gene annotation (Leroy et al., 2012). In addition, a homology search was performed against the NCBI non-redundant databases using BLASTN, BLASTX, and TBLASTX algorithm to verify annotated genes and identify missed genes and pseudogenes. Because gene annotation often includes transposable elements, only genes that have homology in other monocots were included.

### Transcriptome Data Analysis

To analyze the expression of prolamin genes, Chinese Spring RNA-seq data (176.5 Gbp) derived from endosperm tissue at three time points (10, 20 and 30 days post-anthesis) were downloaded from NCBI (ERP004505). The Chinese Spring coding sequences (CDS) (TGAC v1.0) were downloaded from EnsemblPlants^2^. The annotated prolamin gene sequences along with the TGAC CDS (minus the prolamin genes) were used as reference for RNA-Seq analysis using the CLC Genomic Workbench (v8.5) RNA-Seq Analysis Toolbox. Because of the high nucleotide similarities among the prolamin gene family members, stringent mapping parameters with mismatch cost 2, insertion and deletion cost 3, length fraction 0.9, similarity 0.99 were employed in mapping. The FPKM values were calculated using the function in the CLC Toolbox. Manual check of RNA-seq alignment with the target gene was used to confirm the assembly of the prolamin gene sequences, including mutation sites causing pseudogenization.

### Sequence Alignment and Phylogenetic Analysis

For construction of phylogenetic trees, the coding sequences of both prolamin and resistance genes were used. Prolamin genes contain no introns and therefore, the coding sequences can be easily identified through gene annotation. To identify the coding sequences of both *NLR* and *RLK* resistance genes, both hidden Markov model (HMM) and BLAST search were performed (Shao et al., 2014). The identified coding sequences were further examined to see if they encode LRR, CC, NSB, and Kinase domains using Pfam analysis and SMART protein motif analysis^3^. The coding sequences of the genes were aligned in MEGA7 by MUSCLE with default settings (Kumar et al., 2016). The obtained alignments were then subjected to visual inspections and manual adjustments to improve their qualities. Short sequences containing large deletions were removed since they could be often problematic in later phylogenetic analyses. Phylogenetic trees were constructed using the neighbor-joining method in the MEGA7 program with the confidence probability estimated using the bootstrap test with 1000 replications.

## Results

### Reconstruction and Gene Content of Genomic Regions Harboring Prolamin and *R* Loci

To generate high-quality sequences covering the genomic regions harboring multiple prolamin and *R* gene loci on the short arms of the wheat group 1 chromosomes, 103 genes annotated from the orthologous region in *Ae. tauschii* were used in a BLASTN search against the Chinese Spring (CS) genomic sequence data generated by PacBio long reads (Zimin et al., 2017). This CS final assembly is ∼15 Gb in size and has a weighted average (N50) contig size of over 230 kb, representing the most complete and contiguous assembly of the published wheat genome to date (Zimin et al., 2017). The retrieved sequence contigs were then aligned with the Chinese Spring genome map to identify CS BNG map contigs. Since the N50 for the CS optical map is over 1.6 Mb (Huo et al., 2018), 4–5 optical map contigs could span a ∼5 Mb genomic region. The identified optical map contigs were then used to search CS PacBio contigs with RefAligner (BioNano Genomics). Through this analysis, additional sequence contigs belonging to the genomic regions were identified. All the optical map contigs and PacBio sequence contigs were aligned together. In general, gaps between two adjacent contigs are usually not shared by the gaps between two adjacent sequence contigs. Therefore, two adjacent optical map contigs can be often bridged with a sequence contig as shown in Supplementary Figure S1. Through this process, we were able to order optical map contigs to build the optical maps spanning the prolamin genomic regions in Chinese Spring. The optical maps were then used as frameworks to generate sequence scaffolds by aligning, ordering, re-orienting, and merging sequence contigs (Hastie et al., 2013). Three consensus sequences with lengths of 5,335,195, 6,535,908, and 5,639,164 bps were generated for the A, B, and D genomes, respectively. There are only 9, 4, and 15 gaps for the A, B, and D genome sequences, respectively (Supplementary Figure S1). By estimation, ∼92% of the sequences were covered by the optical genome maps.

To better delineate gene duplication and evolution, it is important to also annotate pseudogenes, particularly those among high copy prolamin and *R* genes that are often disrupted by various mechanisms. Automated gene annotation pipelines often only annotate full-length intact genes. However, pseudogenes can be easily identified through manual annotation by BLASTN search of known genes against the genomic region. Therefore, we employed both the automated TriAnnot method (Leroy et al., 2012) and manual annotation process to identify genes in the sequenced regions. In the analyzed regions, 96, 164, and 129 genes were annotated for the A, B, and D genomes, respectively (Supplementary Table S1). As expected, multiple copies of prolamin and *R* genes were detected for each homeologous genome. Pseudogenes accounted for high percentages - 57 out of 88 *NLR*, 19 out of 41 *RLK*, and 25 out of 52 prolamin genes (Supplementary Table S1 and Supplementary Figure S2).

### Comparison of the Orthologous Region of the D Genomes From *Ae. tauschii* and Chinese Spring

**FIGURE 1:**
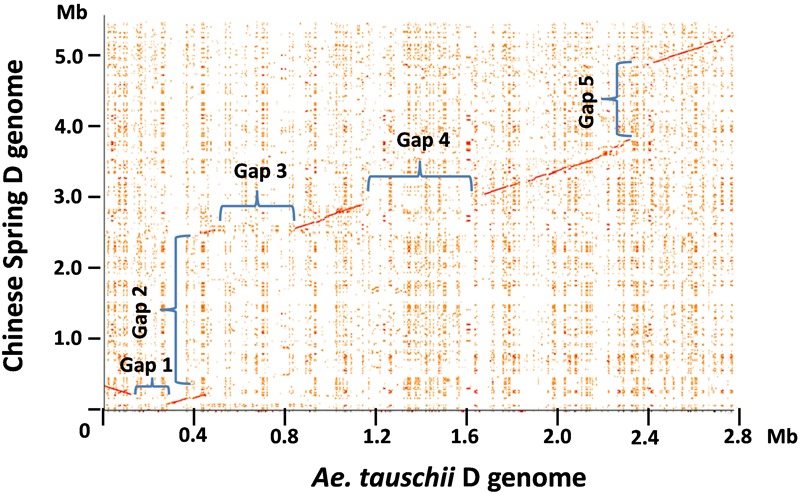
Pairwise comparison of the hexaploid and *Ae. tauschii* D genome regions harboring prolamin and resistance gene families. The dot plot between the orthologous regions from the hexaploid wheat Chinese Spring and diploid *Ae. tauschii* was performed using the r2cat program with default parameter setting (Husemann and Stoye, 2010). Each distinct gap on the dot plot is assigned a number, and the sequences involved in the gap regions are described in the text.

The genomic region carrying the *Glu-3* and *Gli-1* loci in the progenitor D from diploid *Ae. tauschii* has been characterized recently (Dong L. et al., 2016). To delineate the sequence variations in the orthologous regions between the diploid and hexaploid D genomes, we performed a dot matrix analysis as shown in **Figure 1**. In this figure, sequence divergences are seen as disruptions in the main matrix diagonal line. In general, gaps along the diagonal line represent sequence variations that could result from different types of rearrangements including insertions, deletions, inversions, and translocations. A total of five gaps with an estimated size of over 100 kb each were identified in the compared regions, indicating considerable structural variations between the two D genomes. Gap2 is the largest structural variation at over 2 Mb consisting of 34 genes that are present in CS but absent in *Ae. tauschii* AL/78 (**Figure 1** and Supplementary Table S2). Among these genes, two are *RLK* and five are *NLR* genes. In several cases, these *R* genes are interspersed with other gene types. Interestingly, the insertion of this 2-Mb region occurred between two ω-gliadin genes (ω-D1 and ω-D2) in CS, while in *Ae. tauschii*, the two corresponding genes (ω-D^t^1 and ω-D^t^2) were separated from each other by a distance of ∼20 kb (Supplementary Table S2), suggesting that this structural variation occurred between the two ω-gliadin genes. Gap3 is about 340 kb in size with a total of 17 genes that are present in *Ae. tauschii* AL/78 but absent in CS. This represents a high gene density region (one gene/20 kb) and most genes (13) in this region belong to the *NLR* and *RLK* gene families (Supplementary Table S2). Gap4 with a size of ∼525 kb represents a region that is only present in *Ae. tauschii* and contains nine genes with one *NLR*. Gap5 spans a 1.2-Mb region present in CS and contains 17 genes with seven *NLRs* and one LMW-GS. Gap1 is the smallest with a size just over 100 kb and includes two genes that are present in *Ae. tauschii* but absent in CS. These large structural variations reduced the total sequence length that is shared in the two compared regions to only ∼1.6 Mb (56% of the *Ae. tauschii* region, 29% of the CS region). In other words, 44% of the *Ae. tauschii* or 71% of the CS regions are not shared or orthologous in the compared region between the two D genomes, including at least 79 genes (Supplementary Table S2).

To validate these structural variations between the two D genomes, we first located the corresponding positions of structural variations in the two D genome regions and then examined if the sequence assemblies nearby these positions were supported by the BioNano maps (Supplementary Figure S1). Since the sequence accuracy can be all confirmed by the BioNano maps, we eliminated the possibility that the structural variations were due to sequence assembly errors. Next, we used the sequences that are present in one D genome to blast against the entire assembled sequence data of the other D genome that showed absence of the sequences in the compared region. We didn’t retrieve any sequences or sequence contigs that can align well to the present sequence in the regions involved in Gap1, 3, 4, and 5, further supporting that they are presence/absence variations. However, the 2 Mb sequence region present in CS did identify two highly similar sequence regions in *Ae. tauschii*. A dotplot analysis between the CS 2 Mb region with the first 7 Mb region on the short arm of the *Ae. tauschii* chromosome 1 revealed the two matched regions separated by a distance of ∼2 Mb (Supplementary Figure S3). Therefore, the structural variation involved in Gap2 was likely caused by translocation and/or inversion events.

We also examined gene collinearity in the compared regions from the two D genomes. Except for genes located in the structural variation regions, gene collinearity in the aligned regions was well maintained. We identified 71 orthologous gene pairs in the aligned regions and six genes (non-colinear) present in only one of the genomes (Supplementary Table S2). Two non-colinear genes encode prolamins. One is ω-gliadin (ω*-D3*), which is 12 kb away from an adjacent ω-gliadin gene (ω*-D2*). These genes are 100% identical in the coding region, and there is only one SNP (T/C) at -165 upstream from the start codon. It is likely that ω*-D3* originated from a recent tandem duplication of ω*-D2* in the CS D genome. The other one is a LMW-GS pseudogene (*LMW-D4*). Although *LMW-D4* is most closely related to the intact *LMW-D3* genes (94% nucleotide identity), *LMW-D3* and *LMW-D4* are 114 kb apart, with a JA-induced gene between them. It is unclear if *LMW-D4* originated from a duplication event in the Chinese Spring D genome, or if there was a deletion event that removed its counterpart in *Ae. tauschii* AL8/78. We noticed that three of the non-colinear genes were adjacent to the large structural variations (Supplementary Table S2). In these cases, the non-colinear genes were absent in the genome containing the extra genes from the structural variation events. One possible explanation is that the insertion of a DNA fragment could be accompanied by a sequence or gene deletion in the recipient site due to DNA breakage.

### Gene Collinearity in the Homeologous Regions of the A, B, and D Genomes

**FIGURE 2:**
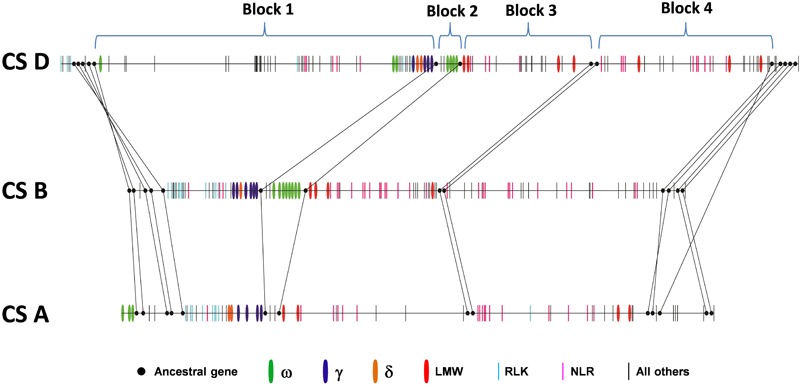
Collinearity of orthologous regions harboring prolamin and resistance gene families from the wheat A, B, and D genomes. Ancestral genes that are shared with grass genomes are shown in solid dots and connected with lines. Other genes are represented by vertical bars with colors to indicate different types of prolamins and resistance genes. Black bars represent genes other than prolamin or resistance genes.

Collinearity analysis was also performed in the orthologous regions of the A, B, and D genomes. Previous analysis based on the comparison of the *Ae. tauschii* region with orthologous regions from *Brachypodium*, rice and sorghum identified 14 ancestral genes shared among all genomes analyzed (Dong L. et al., 2016). We found that these genes were also maintained in the A, B and D genomes with the exception of one ancestral gene that was missing in the B genome (**Figure 2** and Supplementary Table S1). These ancestral genes were used to define four syntenic blocks to facilitate collinearity analysis (**Figure 2** and Supplementary Table S1). We found that gene number differed greatly among these syntenic blocks. For example, in Block 1, the D genome had 51 genes, the B genome had 29 genes, and the A genome had only 18 genes. In addition, the gene content in syntenic blocks could be quite different. In Block 2, both the D and B genome contained multiple copies of ω-gliadin genes, while ω-gliadin genes were absent in the A genome (Supplementary Table S1). It is possible that these ω-gliadin genes located between amylase inhibitor genes (AI) and LMW-GS genes have been deleted from the A genome. In Block 1, genes in the A and B genomes were primarily prolamin and *R* genes, while many other types of genes were present in the D genome region (Supplementary Table S1). These non-colinear genes were mainly derived from the Gap2 region described in the previous section, supporting the notion that the large structural variation occurred only in the Chinese Spring D genome. Furthermore, in Block 1, there were no orthologous ω-gliadin genes in either A or B genomes. However, in the A genome, three ω-gliadin genes are found in the region before Block 1, suggesting a possible translocation event (Supplementary Table S1).

Besides the variations in gene number and content in syntenic blocks, the existence of multiple copies of prolamin and/or *R* gene family members in each block also complicated collinearity analyses in identifying orthologous counterparts. Orthologous genes evolve from a common ancestral gene by speciation, while paralogous genes are derived from gene duplication within a species. Therefore, a single gene in a species could have multiple orthologs after duplication in the genomes of different species. Therefore, the relationship of gene family members (ortholog or paralog) is often difficult to determine based on the linear order of genes in the analyzed regions. This is the case for prolamin and *R* genes in the analyzed regions. We found that the orthologous relationships among the A, B, and D genomes could not be unambiguously delineated based solely on gene contents and order, although syntenic blocks were clearly defined (Supplementary Table S1).

### Phylogenetic Analysis of Wheat Prolamin Genes

**FIGURE 3:**
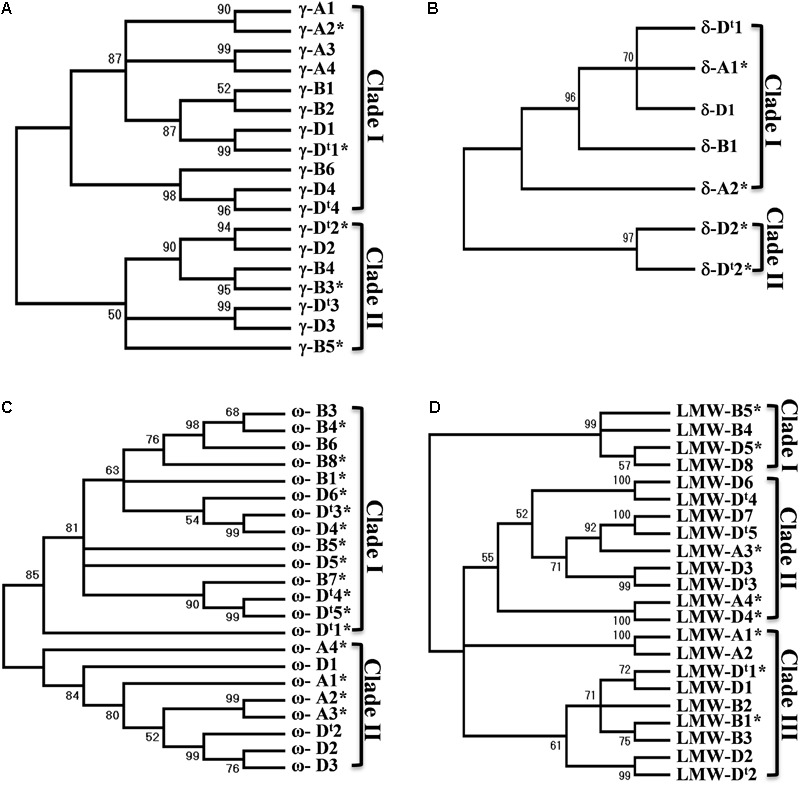
Phylogenetic trees for prolamin genes. Nucleotide sequences of different groups of prolamin genes from the A, B, and D genomes of Chinese Spring and D genome of *Ae. tauschii* were used for phylogenetic analysis. Phylogenetic trees (**A** for γ-gliadin, **B** for δ-gliadin, **C** for ω-gliadin, and **D** for LMW-GS) were reconstructed using MEGA7 with the Neighbor-Joining method. The bootstrap consensus tree inferred from 1000 replicates is taken to represent the evolutionary history of the prolamin genes analyzed. The percentage of replicate trees in which the associated genes clustered together in the bootstrap test (1000 replicates) are shown next to the branches. Sequences from *Ae. tauschii* are indicated with ^t^.

A phylogenetic tree often provides useful information about the evolutionary relationship of gene family members. Phylogenetic trees were then reconstructed for each group of prolamin genes in the analyzed regions (Supplementary Figure S2). A total of 14 γ-gliadin genes were identified in the CS genome, and were grouped into two clades. All four γ-gliadins from the A genome were in one clade, while γ-gliadins from the D and B genomes were present in both clades (**Figure 3A**), suggesting that the A genome might have lost the genes corresponding to those in Clade I. The four genes from the D genome were all clustered with the homologous copies from *Ae. tauschii*, supporting the notion that there are no significant changes in γ-gliadins between the hexaploid and progenitor D genomes. δ-gliadins, a new class of wheat gliadins recognized recently, likely existed in an ancestral Triticeae species prior to the divergence of wheat and barley (Anderson et al., 2012). Five δ-gliadins were identified in CS. The A and D genomes each had two copies separated by less than 20 kb (Supplementary Table S1), whereas the B genome had only one full-length copy in Clade I (**Figure 3B**), suggesting a possible deletion event. A total of 19 ω-gliadin genes were identified, including one (ω*-A4*) that was not located within the analyzed region. These genes were divided into two main clades: one containing genes from the D and B genomes, and the other from the D and A genomes (**Figure 3C**). It is possible that both A and B genome have differentially lost or duplicated some ω-gliadin genes or such a result may reflect a hybrid origin of the D genome from a cross between A and B as proposed recently (Marcussen et al., 2014).

Among the 17 LMW-GS genes identified in CS, the A genome has four copies and B genome has five copies, while the D genome has eight copies (**Figure 3D**). *LMW-D8* (full-length) and *LMW-B5* (pseudogene) were not located in the analyzed regions and could represent copies that have been translocated to different regions (Supplementary Figure S2). The LMW-GS genes were grouped into three clades (**Figure 3D**). Clade III contained genes from all the three genomes. Genes in Clade II are from the A and D genome, while Clade I only had B and D genome genes, suggesting that differential gene duplications and deletions of LMW-GS genes have taken place after the separation of the A, B, and D genomes. Taken together, the prolamin gene families in *Glu-3* and *Gli-1* existed before the divergence of the A, B and D genomes, but also evolved independently since then.

### Phylogenetic Analysis of *R* Genes

Nucleotide-binding domain and leucine-rich repeat and RLK genes are both highly duplicated in the analyzed regions. Previously, we showed that NLR genes in *Ae. tauschii* region can be divided into different subgroups and suggested that these *R* genes were likely translocated into the current position from multiple genomic regions and expanded via gene duplication, while the high number of RLK genes is primarily due to local duplication (Dong L. et al., 2016). When the NLR phylogenetic tree included the *R* genes from the homoelogous genomes, it was noted that each subgroup (PM3, LR21, and RPP13) contained genes from all the subgenomes (Supplementary Figure S4), suggesting that these subgroups existed before the divergence of the A, B, and D genomes. The result can also help identify candidate orthologous genes. However, we also observed that *R* genes from the syntenic blocks defined by the ancestral genes were not always associated in the same branches or subgroups. Moreover, genes from different syntenic blocks could be grouped in the same branch or more closely related (Supplementary Figure S4). Similar results were also observed for RLK genes (Supplementary Figure S5). In addition, we noted that many branches contained genes only from two of the genomes, a result also seen in the phylogenetic analysis of prolamin genes. Again, it is not clear if such observation reflects rapid reshuffling of *R* genes in the different wheat genomes or is due to the hybrid origin of the D genome (Marcussen et al., 2014).

### Transcriptome Analysis of Prolamin Gene Expression

**FIGURE 4:**
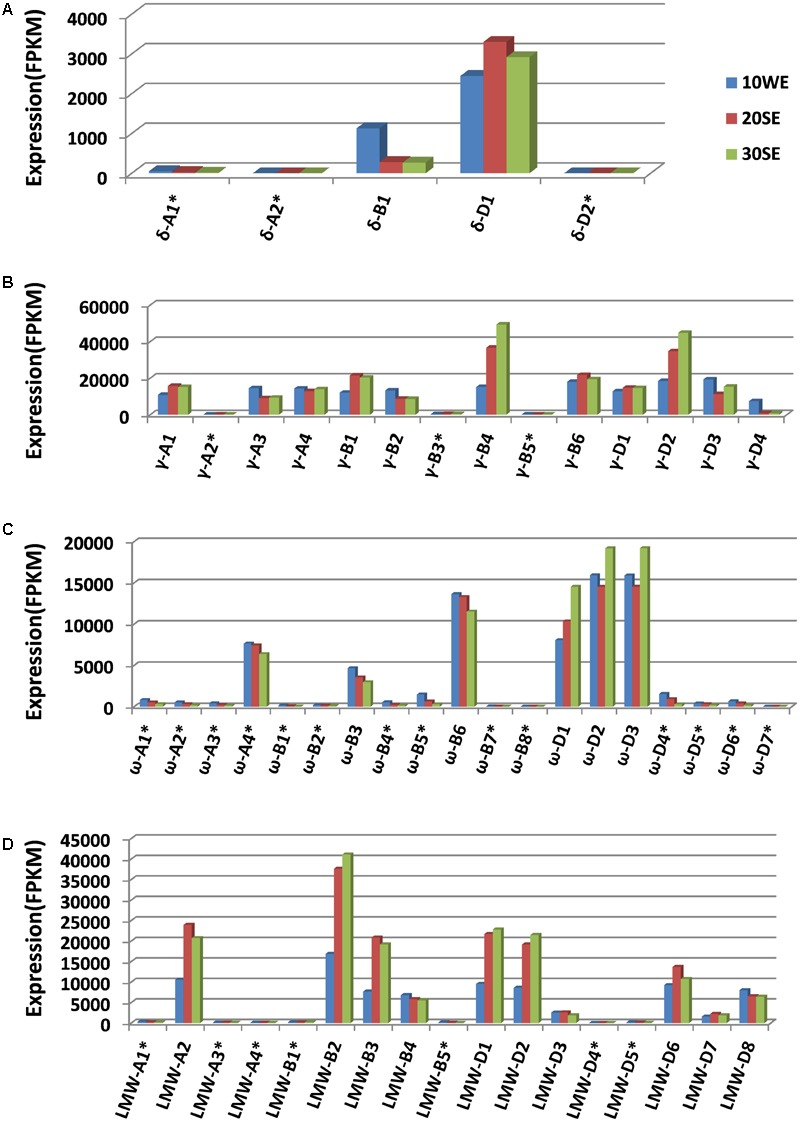
Expression profiles of different groups of prolamin genes (**A** for γ-gliadin, **B** for δ-gliadin, **C** for ω-gliadin, and **D** for LMW-GS). Transcriptome datasets generated from different grain development stages were downloaded from the published result (Pfeifer et al., 2014). 10WE represents whole endosperm at 10 days post-anthesis, 20SE, starchy endosperm at 20 days post-anthesis, 30SE, starchy endosperm at 30 days post-anthesis. For expression profiling, CLC genomic workbench RNA-seq analysis toolbox was used to map transcriptome reads to the complete annotated wheat gene set (Clavijo et al., 2017) with the prolamin genes replaced with the prolamin gene set annotated in this study. FPKM values were calculated using the functions in the toolbox. Pseudogenes are indicated with ^∗^.

Wheat prolamin genes are known to be specifically expressed in endosperm tissues during grain development. The identification of a full complement of prolamin genes allowed us to map transcriptome reads to individual prolamin genes, providing a more accurate view of their expression. Mapping the transcriptome reads to the annotated gene set is also useful in validating the prolamin gene assembly manually (Huo et al., 2017). Through this process, all 27 prolamin pseudogene sequences were confirmed, indicating that the high rate of pseudogenes (49%) is likely associated with the dynamic sequence evolution in the analyzed regions. The expression of pseudogenes is very low compared to intact genes (**Figure 4**). This is expected as the transcripts of pseudogenes are unstable and regulated at the post-transcriptional level by a mechanism called nonsense-mediated mRNA decay (Hug et al., 2016). However, the expression level of pseudogene ω*-A4* was found to be somewhat higher than that of the intact gene ω*-B3*. When the ω*-A4* coding sequence was analyzed, a premature stop codon was identified near the end of the coding region that would result in a protein that is 12 amino acids shorter than the expected full-length translation product (360 vs. 372 amino acids).

We also examined the expression of each prolamin gene group from the A, B, and D genomes. The expression of δ-gliadin genes is mostly contributed by δ*-D1*. In CS, both δ-gliadin genes from the A genome are pseudogenes and do not appear to be expressed (**Figure 4A**). Most γ-gliadin genes are intact. The expression of each intact gene is approximately equivalent, except γ*-B4* and γ*-D2* with slightly higher expression and γ*-D4* with somewhat lower expression (**Figure 4B**). Fourteen of 19 ω-gliadin genes are pseudogenes with little to no expression. Among the six ω-gliadin genes with FPKM values above 200 (including ω*-A4* that contains a stop codon at the end of the coding region), three are from the D genome (**Figure 4C**). Therefore, the contribution of the D genome to ω-gliadins is greater than the A and B genomes combined. Among ten intact LMW-GS genes, only one is from the A genome, three from the B genome, and the rest from the D genome (**Figure 4D**). For this group of prolamins, it appears that the A genome contributes the least to expression, considering its small number of expressed intact genes and their low expression levels, while the D and B genomes seem to contribute similarly.

The large number of sequence reads in the Chinese Spring transcriptome data allowed us to assess abundances of prolamin transcripts in the endosperm tissue. This was accomplished by calculating the number of transcript reads mapped to each prolamin genes divided by the total number of reads mapped to the complete annotated wheat genes set (Supplementary Table S3). In this analysis, HMW-GS and α-gliadin genes were also included to provide a better view of the relative transcript abundance of each prolamin gene group in hexaploid wheat. At 30 days post-anthesis, γ-gliadin, α-gliadin, and LMW-GS transcripts account for 20.55, 19.39, and 16.2% of the total mapped reads, respectively. ω-gliadin and HMW-GS transcripts each account for over 7% of total, while δ-gliadin transcripts account for only 0.3%. Again, we observed that the contribution of A genome to the transcripts of each prolamin group was considerably less than the B and D genomes (Supplementary Table S3). Nevertheless, the transcripts from all the prolamin groups account for about 70% of the total mapped reads, indicating the high abundance of prolamin transcripts.

## Discussion

The genomic regions carrying multiple prolamin loci on the short arm of wheat group 1 chromosomes are hotspots of duplication for prolamin and *R* genes, making their accurate sequence assembly very challenging. In this work, we took advantage of Chinese Spring long sequence contigs from PacBio long reads (Zimin et al., 2017) and the BioNano genome map to reconstruct the orthologous regions carrying both prolamin and *R* gene families from the wheat A, B, and D genomes. The BioNano genome map is useful for generating high-quality sequence assemblies to resolve these complex regions by validating, orientating, and ordering sequence contigs. It also serves as a tool to confirm the structural variations observed in hexaploid wheat and its D genome progenitor. This work represents the first report highlighting the evolutionary dynamics of the genomic regions harboring prolamin and *R* gene families in a hexaploid wheat species.

### Structural Variations of the D Genomes of *Ae. tauschii* and Hexaploid Wheat

*Aegilops tauschii*, the donor of the wheat D genome, has been widely employed to investigate molecular changes during and after wheat polyploidization (Li et al., 2015). In this study, a detailed sequence comparison revealed dramatic structural variations in the analyzed orthologous regions from *Ae. tauschii* AL8/78 and hexaploid wheat Chinese Spring. Five large structural variations with sizes over 100 kb were detected. Consequently, in the two compared D regions, only 56% of the *Ae. tauschii* and 29% of the CS sequences are conserved, while the rest of the sequences are unique either to the *Ae. tauschii* or CS region. We noted that the sequences involved in four structural variations are presence/absence variations between the two genomes, while one structural variation involved in a 2-Mb sequence region is likely caused by translocation and/or inversion events. Such a variation can result in considerable erosion of gene collinearity (gene content and order) in the compared region. However, these genes are still present in the genome. Given the short divergence time of the two D genomes and the high conservation in those aligned regions, it was quite unexpected to find multiple large structural variations, particularly those as extreme as the 2-Mb region containing 34 genes. The mechanism underlying these large structural variations is unclear. However, since *RLK* and *NLR* genes are distributed across the analyzed regions, including those involved in the structural variations, it is likely that these large structural variations represent local sequence rearrangements, rather than translocations from other places. Nevertheless, they are responsible for the major difference in the two D regions, including 79 non-shared or non-colinear genes. It has been proposed that the frequent structural variations observed among maize inbreds may contribute to the high levels of genetic and phenotypic diversity (Springer et al., 2009). When different structural variants are combined naturally or through breeding, there is opportunity for formation of novel alleles *via* unequal crossing over. Allelic variations of prolamin and *R* genes are particularly important for the improvement of grain end-use quality and disease resistance phenotypes. Since only the Chinese Spring and *Ae. tauschii* AL8/78 D genomes were examined in this study, at this point, it is not clear if the structural variations exist at the diploid level or occurred in the hexaploid genome. Further characterization is needed to understand their allelic variations in relation to phenotypic differences at the genomic sequence level for crop improvement. Meanwhile, it is also unclear if large structural variations are evenly distributed across the genome or biased depending on chromosomal locations. The genomic regions in this study are known to locate in the distal end of the chromosome where frequent sequence changes are likely due to the high rate of recombination events (Akhunov et al., 2003; Dvorak and Akhunov, 2005; Choulet et al., 2010; Dong L. et al., 2016). Further study on the association of large structural variations with recombination is required.

### Rapid and Dynamic Evolution of Prolamin and *R* Gene Regions in the A, B, and D Genomes

In comparison with related grass model species, the *Ae. tauschii* 1DS region containing prolamin and *R* genes has undergone dramatic evolutionary changes as evidenced by the high number of non-syntenic genes being six times more than conserved ancestral genes (Dong L. et al., 2016). This high rate of non-syntenic genes reflects the fast evolution of Triticeae species (Glover et al., 2015; Luo et al., 2017). It appears that the insertion of non-syntenic genes followed by local duplications played important roles in gene expansion in this prolamin and *R* gene region (Dong L. et al., 2016). Prolamin and *R* gene families are among the non-syntenic genes in the *Ae. tauschii* region, suggesting that they evolved rapidly in Triticeae lineage since its separation from the other grass species around 25–30 MYA. Prior to this study, their genomic organization and evolution in different Triticeae genomes remained unclear. The A, B, and D genomes of hexaploid wheat are closely related with their divergence time from a common progenitor some 2.5–4.5 MYA (Huang et al., 2002). A recent study suggests that the genome donors of hexaploid wheat diverged only between 2.1 and 2.9 MYA (Middleton et al., 2014). Therefore, the wheat A, B, and D genomes provide a window into the recent evolution of prolamin and *R* genes. Our comparative analysis based on syntenic blocks revealed considerable variations with respect to gene numbers and contents, suggesting that sequence changes including gene duplication, deletion, and translocation might have occurred frequently and independently in different wheat genomes. Although the phylogenetic tree analyses of prolamin and *R* genes facilitated the identification of orthologs that existed prior to the divergence of the A, B and D genomes, they also confirmed frequent occurrences of genome-specific duplication of both prolamin and resistance genes after their separation. Considering the large variations in gene numbers and contents in the orthologous regions, our results support continuous and rapid evolution of both prolamin and *R* genes in the Triticeae genomes.

Triticeae genomes are known to evolve rapidly in comparison to compact genomes such as *Brachypodium*, sorghum, and rice (Luo et al., 2013; Glover et al., 2015; Luo et al., 2017). However, detailed comparative analyses among the Triticeae genomes have only been reported in a few genetic loci (Gu et al., 2004; Chantret et al., 2005; Zhang et al., 2011). It has been shown that the sequences of intergenic regions consisting of repetitive DNAs are completely diverged in the A, B, and D genomes due to the rapid amplification and fast turnover of transposable elements (Gu et al., 2004). The overall collinearity among the A, B and D genomes is less clear. Well-maintained collinearity was observed in the orthologous HMW-GS regions (Gu et al., 2004), while in the hardness loci, the loss of *Pina* and *Pinb* genes was caused by deletions that occurred independently in the A and B genomes (Chantret et al., 2005). A large-scale comparison at contiguous sequence levels was only recently reported for the short arms of *Ae. tauschii* 3D (At3DS) and Chinese Spring 3B (Ta3BS) (Xie et al., 2017). The result indicated that only 58.23% of At3DS genes and 47.2% of Ta3BS genes have orthologous gene pairs, suggesting that a large proportion of the genes have been rearranged since the divergence of the two genomes. However, the driving force for the dynamic changes among the Triticeae genomes is not well understood. It has been shown that recombination is positively or negatively associated with several genome structure features, including gene density, gene deletion and duplication, SNP rate, and TE distribution. (Akhunov et al., 2003; Dvorak and Akhunov, 2005; Glover et al., 2015; Luo et al., 2017; Xie et al., 2017). Coincidently, the prolamin and *R* loci in *Ae. tauschii* are located in the distal chromosomal region where the recombination rate is high with a cross-over (CO) frequency of 3.14 cM/Mb, over ninefold higher than the average for chromosome 1D (Dong L. et al., 2016). Therefore, the high recombination rate may play the central role in shaping the structure and evolution of this agronomically important region.

### Evolution of Prolamin and *R* Gene Families

Genomic structural variation that contributes to the overall fitness of the organism can be naturally selected and fixed in the population. The evolution of wheat prolamins is likely involved in speciation while the duplication and evolution of disease resistance genes are key sources for adaptation to the changing environment. Both gene families originated as non-syntenic genes as they are absent in the orthologous regions of rice and sorghum genomes (Dong L. et al., 2016). It has been proposed that the prolamin genes in *Glu-3* and *Gli-1* loci might originate from a single translocation event followed by further amplification and evolution to form different types of prolamin groups (LMW-GS, γ-, ω- δ-gliadins) with distinct domain structures (Dong L. et al., 2016). There are only three genomic regions containing prolamin genes in a wheat genome. HMW-GS originated from a duplication of an ancestral globulin gene that occurred before the divergence of Triticeace and *Brachypodium* lineage (Xu and Messing, 2009; Gu et al., 2010). Therefore, one can speculate that the prolamin genes in *Glu-3* and *Gli-1* loci might originate from an ancestral gene related to HMW-GS. We exclude α-gliadins from the origin of *Glu-3* and *Gli-1* loci because they are the youngest group of wheat prolamin that evolved after the divergence of wheat and rye ∼7–8 million years ago (Huo et al., 2017). On the contrary, *R* genes are present in many genetic loci. The *R* genes in the analyzed region likely originated from multiple translocation events (Dong L. et al., 2016). For instance, among the NLR group, *R* genes can be divided into RPP13, RPM3, PM3, and LR21 subfamilies that each has closer homologs located in different genomic regions (Dong L. et al., 2016). The current study indicated that the genomic regions might continuously experience structural changes as both the prolamin and *R* gene families showed considerable erosion of collinearity in the A, B, and D genomes, likely resulting from gene duplication, deletion, and translocation etc. Both prolamins and *R* genes are known to evolve dynamically and rapidly. They are often prone to duplication. The observed high number of prolamin and resistance genes indicated that sequence duplications have occurred frequently in the genomic regions. In the case that a duplication of a prolamin gene spans a region containing a resistance gene, both genes will be duplicated. The fact that the two gene families are intermingled at the genomic regions suggests that such a case is frequent. Therefore, co-localization of prolamin and *R* genes might have accelerated their evolution in the genomic regions. In addition, the high content of TEs in the wheat genome can encourage ectopic recombination in which crossing over occurs at non-homologous, rather than along homologous, loci. Such recombination can cause dramatic chromosome rearrangement and gene copy number variations (Kent et al., 2017). Nevertheless, because of their physical closeness, selection naturally or through wheat breeding for the resistance allele will bring together the phenotype of the linked prolamin allele or vice versa. This provides partial explanations to the association of good resistance traits with poor end-use quality in some wheat germplasm or cultivars (Bonafede et al., 2015).

### Expression and Function of Wheat Prolamins

The end-use property of wheat grain is controlled by both quantity and quality of prolamins (Shewry et al., 2002). Therefore, determining the expression of individual prolamin genes and understanding contribution of each to the overall gluten content and functionality will facilitate the development of wheat varieties with improved end-use properties. However, because of the complexity of their large gene families, unique repeat domains in the coding region, and lack of complete sequence information, expression analysis of prolamin genes using PCR-based approach or *de novo* assembly of transcriptome reads has been challenging. In this study, a full complement of prolamin gene sequences in the *Glu-3* and *Gli-1* loci from a single wheat accession were generated using long PacBio reads with sequence errors corrected with Illumina reads. We found that corrected PacBio reads are superior to Illumina short reads in sequencing prolamin genes, particularly these ω-gliadin genes that contain long stretches of repeated domains (Anderson et al., 2009) (Supplementary Table S4). With the complete set of prolamin gene sequences, we profiled expression by mapping transcriptome reads, providing a detailed view of how individual genes and genomes contribute to the overall prolamin expression. Of 55 prolamin genes identified (Supplementary Figure S2), 29 genes likely encode complete proteins, including 11 γ-gliadins, two δ-gliadins, five ω-gliadins, and ten LMW-GS. Considerable allelic variations exist for the end-use quality among wheat cultivars (Bonafede et al., 2015; Cuesta et al., 2015). An important question is how allelic variations among prolamins in different wheat genotypes influence both the technological and immunogenic properties of the flour. To this end, the knowledge gained from this study combined with similar studies in other genotypes will facilitate proteomic studies that can address this question and ultimately lead to the development of new strategies to improve the end-use quality and healthfulness of wheat.

## Author Contributions

NH, SZ, YW, and TZ performed the experiment and generated data. NH, TZ, SZ, SA, TH, LD, DW, TM, ZL, JD, M-CL, J-YL, and YG participated in the data analysis as well as in preparation of the manuscript. YG and SA managed the research project. YG wrote the manuscript. All authors read and approved the manuscript.

## Conflict of Interest Statement

The authors declare that the research was conducted in the absence of any commercial or financial relationships that could be construed as a potential conflict of interest. The reviewer MM and handling Editor declared their shared affiliation.

## Footnotes

1 http://www.bionanogenomics.com/support/software-updates/

2 ftp://ftp.ensemblgenomes.org/pub/plants/release-37/fasta/triticum_aestivum/cds/

3 http://smart.embl-heidelberg.de/

## References

[B1] Akhunov E. D. Akhunova A. R. Linkiewicz A. M. Dubcovsky J. Hummel D. Lazo G. (2003). Synteny perturbations between wheat homoeologous chromosomes caused by locus duplications and deletions correlate with recombination rates. 100 10836–10841. 10.1073/pnas.1934431100 12960374 PMC196889

[B2] Alix K. Gerard P. R. Schwarzacher T. Heslop-Harrison J. S. P. (2017). Polyploidy and interspecific hybridization: partners for adaptation, speciation and evolution in plants. 120 183–194. 10.1093/aob/mcx079 28854567 PMC5737848

[B3] Altenbach S. B. Tanaka C. K. Hurkman W. J. Whitehand L. C. Vensel W. H. Dupont F. M. (2011). Differential effects of a post-anthesis fertilizer regimen on the wheat flour proteome determined by quantitative 2-DE. 9:46. 10.1186/1477-5956-9-46 21816081 PMC3168407

[B4] Anderson O. D. Dong L. Huo N. Gu Y. Q. (2012). A new class of wheat gliadin genes and proteins. 7:e52139. 10.1371/journal.pone.0052139 23284903 PMC3527421

[B5] Anderson O. D. Gu Y. Q. Kong X. Lazo G. R. Wu J. (2009). The wheat omega-gliadin genes: structure and EST analysis. 9 397–410. 10.1007/s10142-009-0122-2 19367421 PMC2700870

[B6] Bonafede M. D. Tranquilli G. Pflüger L. A. Peña R. J. Dubcovsky J. (2015). Effect of allelic variation at the Glu-3/Gli-1 loci on breadmaking quality parameters in hexaploid wheat (*Triticum aestivum* L.). 62 143–150. 10.1016/j.jcs.2015.02.001 27818572 PMC5096839

[B7] Cao H. Hastie A. R. Cao D. Lam E. T. Sun Y. Huang H. (2014). Rapid detection of structural variation in a human genome using nanochannel-based genome mapping technology. 3:34. 10.1186/2047-217X-3-34 25671094 PMC4322599

[B8] Chantret N. Salse J. Sabot F. Rahman S. Bellec A. Laubin B. (2005). Molecular basis of evolutionary events that shaped the hardness locus in diploid and polyploid wheat species (Triticum and Aegilops). 17 1033–1045. 10.1105/tpc.104.029181 15749759 PMC1087984

[B9] Chen Z. J. (2007). Genetic and epigenetic mechanisms for gene expression and phenotypic variation in plant polyploids. 58 377–406. 10.1146/annurev.arplant.58.032806.103835 17280525 PMC1949485

[B10] Choulet F. Wicker T. Rustenholz C. Paux E. Salse J. Leroy P. (2010). Megabase level sequencing reveals contrasted organization and evolution patterns of the wheat gene and transposable element spaces. 22 1686–1701. 10.1105/tpc.110.074187 20581307 PMC2910976

[B11] Clavijo B. J. Venturini L. Schudoma C. Accinelli G. G. Kaithakottil G. Wright J. (2017). An improved assembly and annotation of the allohexaploid wheat genome identifies complete families of agronomic genes and provides genomic evidence for chromosomal translocations. 27 885–896. 10.1101/gr.217117.116 28420692 PMC5411782

[B12] Cuesta S. Guzman C. Alvarez J. B. (2015). Molecular characterization of novel LMW-i glutenin subunit genes from *Triticum urartu* Thum. ex Gandil. 128 2155–2165. 10.1007/s00122-015-2574-1 26152575

[B13] Dong J. Feng Y. Kumar D. Zhang W. Zhu T. Luo M. C. (2016). Analysis of tandem gene copies in maize chromosomal regions reconstructed from long sequence reads. 113 7949–7956. 10.1073/pnas.1608775113 27354512 PMC4961126

[B14] Dong L. Huo N. Wang Y. Deal K. Wang D. Hu T. (2016). Rapid evolutionary dynamics in a 2.8-Mb chromosomal region containing multiple prolamin and resistance gene families in *Aegilops tauschii*. 87 495–506. 10.1111/tpj.13214 27228577

[B15] Dong L. Zhang X. Liu D. Fan H. Sun J. Zhang Z. (2010). New insights into the organization, recombination, expression and functional mechanism of low molecular weight glutenin subunit genes in bread wheat. 5:e13548. 10.1371/journal.pone.0013548 20975830 PMC2958824

[B16] Dvorak J. Akhunov E. D. (2005). Tempos of gene locus deletions and duplications and their relationship to recombination rate during diploid and polyploid evolution in the Aegilops-Triticum alliance. 171 323–332. 10.1534/genetics.105.041632 15996988 PMC1456522

[B17] Feldman M. Levy A. A. (2012). Genome evolution due to allopolyploidization in wheat. 192 763–774. 10.1534/genetics.112.146316 23135324 PMC3522158

[B18] Freeling M. Scanlon M. J. Fowler J. E. (2015). Fractionation and subfunctionalization following genome duplications: mechanisms that drive gene content and their consequences. 35 110–118. 10.1016/j.gde.2015.11.002 26657818

[B19] Glover N. M. Daron J. Pingault L. Vandepoele K. Paux E. Feuillet C. (2015). Small-scale gene duplications played a major role in the recent evolution of wheat chromosome 3B. 16:188. 10.1186/s13059-015-0754-6 26353816 PMC4563886

[B20] Gu Y. Q. Coleman-Derr D. Kong X. Anderson O. D. (2004). Rapid genome evolution revealed by comparative sequence analysis of orthologous regions from four triticeae genomes. 135 459–470. 10.1104/pp.103.038083 15122014 PMC429398

[B21] Gu Y. Q. Wanjugi H. Coleman-Derr D. Kong X. Anderson O. D. (2010). Conserved globulin gene across eight grass genomes identify fundamental units of the loci encoding seed storage proteins. 10 111–122. 10.1007/s10142-009-0135-x 19707805

[B22] Hastie A. R. Dong L. Smith A. Finklestein J. Lam E. T. Huo N. (2013). Rapid genome mapping in nanochannel arrays for highly complete and accurate de novo sequence assembly of the complex *Aegilops tauschii* genome. 8:e55864. 10.1371/journal.pone.0055864 23405223 PMC3566107

[B23] Huang L. Brooks S. A. Li W. Fellers J. P. Trick H. N. Gill B. S. (2003). Map-based cloning of leaf rust resistance gene Lr21 from the large and polyploid genome of bread wheat. 164 655–664. 12807786 10.1093/genetics/164.2.655 PMC1462593

[B24] Huang S. Sirikhachornkit A. Su X. Faris J. Gill B. Haselkorn R. (2002). Genes encoding plastid acetyl-CoA carboxylase and 3-phosphoglycerate kinase of the *Triticum*/*Aegilops* complex and the evolutionary history of polyploid wheat. 99 8133–8138. 10.1073/pnas.072223799 12060759 PMC123033

[B25] Huang X. Q. Cloutier S. (2008). Molecular characterization and genomic organization of low molecular weight glutenin subunit genes at the Glu-3 loci in hexaploid wheat (*Triticum aestivum* L.). 116 953–966. 10.1007/s00122-008-0727-1 18305921

[B26] Hug N. Longman D. Cáceres J. F. (2016). Mechanism and regulation of the nonsense-mediated decay pathway. 44 1483–1495. 10.1093/nar/gkw010 26773057 PMC4770240

[B27] Huo N. Dong L. Zhang S. Wang Y. Zhu T. Mohr T. (2017). New insights into structural organization and gene duplication in a 1.75-Mb genomic region harboring the α-gliadin gene family in *Aegilops tauschii*, the source of wheat D genome. 92 571–583. 10.1111/tpj.13675 28857322

[B28] Huo N. Zhu T. Altenbach S. Dong L. Wang W. Mohr T. (2018). Dynamic evolution of α-gliadin gene family in homeologous genomes of hexaploid wheat. 8:5181. 10.1038/s41598-018-23570-5 29581476 PMC5980091

[B29] Husemann P. Stoye J. (2010). r2cat:syneny plots and comparative assembly. 26 570–571. 10.1093/bioinformatics/btp690 20015948 PMC2820676

[B30] Kent T. V. Uzunovic J. Wright S. L. (2017). Coevolution between transposable elements and recombination. 372:20160458. 10.1098/rstb.2016.0458 29109221 PMC5698620

[B31] Kondrashov F. A. (2012). Gene duplication as a mechanism of genomic adaptation to a changing environment. 279 5048–5057. 10.1098/rspb.2012.1108 22977152 PMC3497230

[B32] Kumar S. Stecher G. Tamura K. (2016). MEGA7: molecular evolutionary genetics analysis version 7.0 for bigger datasets. 33 1870–1874. 10.1093/molbev/msw054 27004904 PMC8210823

[B33] Lam E. T. Hastie A. Lin C. Ehrlich D. Das S. K. Austin M. D. (2012). Genome mapping on nanochannel arrays for structural variation analysis and sequence assembly. 30 771–776. 10.1038/nbt.2303 22797562 PMC3817024

[B34] Leroy P. Guilhot N. Sakai H. Bernard A. Choulet F. Theil S. (2012). TriAnnot: a versatile and high performance pipeline for the automated annotation of plant genomes. 3:5. 10.3389/fpls.2012.00005 22645565 PMC3355818

[B35] Li A. L. Geng S. F. Zhang L. Q. Liu D. C. Mao L. (2015). Making the bread: insights from newly synthesized allohexaploid wheat. 8 847–859. 10.1016/j.molp.2015.02.016 25747845

[B36] Luo M. C. Gu Y. Q. Puiu D. Wang H. Twardziok S. O. Deal K. R. (2017). Genome sequence of the progenitor of the wheat D genome *Aegilops tauschii*. 551 498–502. 10.1038/nature24486 29143815 PMC7416625

[B37] Luo M. C. Gu Y. Q. You F. M. Deal K. R. Ma Y. Hu Y. (2013). A 4-gigabase physical map unlocks the structure and evolution of the complex genome of *Aegilops tauschii*, the wheat D-genome progenitor. 110 7940–7945. 10.1073/pnas.1219082110 23610408 PMC3651469

[B38] Magadum S. Banerjee U. Murugan P. Gangapur D. Ravikesavan R. (2013). Gene duplication as a major force in evolution. 92 155–161. 10.1007/s12041-013-0212-8 23640422

[B39] Marcussen T. Sandve S. R. Heier L. Spannagl M. Pfeifer M. International Wheat Genome Sequencing Consortium (2014). Ancient hybridizations among the ancestral genomes of bread wheat. 345:1250092. 10.1126/science.1250092 25035499

[B40] Matsuoka Y. (2011). Evolution of polyploid *Triticum* wheats under cultivation: the role of domestication, natural hybridization and allopolyploid speciation in their diversification. 52 750–764. 10.1093/pcp/pcr018 21317146

[B41] Middleton C. P. Senerchia N. Stein N. Akhunov E. D. Keller B. Wicker T. (2014). Sequencing of chloroplast genomes from wheat, barley, rye and their relatives provides a detailed insight into the evolution of the Triticeae tribe. 9:e85761. 10.1371/journal.pone.0085761 24614886 PMC3948623

[B42] Panchy N. Lehti-Shiu M. Shiu S. H. (2016). Evolution of gene duplication in plants. 171 2294–2316. 10.1104/pp.16.00523 27288366 PMC4972278

[B43] Pfeifer M. Kugler K. G. Sandve S. R. Zhan B. Rudi H. Hvidsten T. R. (2014). Genome interplay in the grain transcriptome of hexaploid bread wheat. 345:1250091. 10.1126/science.1250091 25035498

[B44] Shao Z.-Q. Zhang Y.-M. Hang Y.-Y. Xue J.-Y. Zhou G.-C. Ping W. (2014). Long-term evolution of nucleotide-binding site-leucine-rich repeat genes: understanding gained from and beyond the legume family. 166 217–234. 10.1104/pp.114.243626 25052854 PMC4149708

[B45] Shewry P. R. Halford N. G. Belton P. S. Tatham A. S. (2002). The structure and properties of gluten: an elastic protein from wheat grain. 357 133–142. 10.1098/rstb.2001.1024 11911770 PMC1692935

[B46] Shewry P. R. Halford N. G. Lafiandra D. (2003). Genetics of wheat gluten proteins. 49 111–184. 10.1016/S0065-2660(03)01003-4 12779252

[B47] Sollid L. M. Qiao S.-M. Anderson R. P. Gianfrani C. Konig F. (2012). Nomenclature and listing of celiac disease relevant gluten T-cell epitopes restricted by HLA-DQ molecules. 64 455–460. 10.1007/s00251-012-0599-z 22322673 PMC3349865

[B48] Soltis P. S. Marchant D. B. Van De Peer Y. Soltis D. E. (2015). Polyploidy and genome evolution in plants. 35 119–125. 10.1016/j.gde.2015.11.003 26656231

[B49] Spielmeyer W. Lagudah S. (2003). Homoeologous set of NBS-LRR genes located at leaf and stripe rust resistance loci on short arms of chromosome 1 of wheat. 3 86–90. 12590346 10.1007/s10142-002-0074-2

[B50] Springer N. M. Ying K. Fu Y. Ji T. Yeh C. T. Jia Y. (2009). Maize inbreds exhibit high levels of copy number variation (CNV) and presence/absence variation (PAV) in genome content. 5:e1000734. 10.1371/journal.pgen.1000734 19956538 PMC2780416

[B51] Van de Peer Y. Mizrachi E. Marchal K. (2017). The evolutionary significance of polyploidy. 18 411–424. 10.1038/nrg.2017.26 28502977

[B52] Xie J. Huo N. Zhou S. Wang Y. Guo G. Deal K. R. (2017). Sequencing and comparative analyses of *Aegilops tauschii* chromosome arm 3DS reveal rapid evolution of Triticeae genomes. 44 51–61. 10.1016/j.jgg.2016.09.005 27765484

[B53] Xu J. H. Messing J. (2009). Amplification of prolamin storage protein genes in different subfamilies of the *Poaceae*. 119 1397–1412. 10.1007/s00122-009-1143-x 19727653

[B54] Yahiaoui N. Srichumpa P. Dudler R. Keller B. (2004). Genome analysis at different ploidy levels allows cloning of the powdery mildew resistance gene Pm3b from hexaploid wheat. 37 528–538. 10.1046/j.1365-313X.2003.01977.x 14756761

[B55] Zhang Z. Belcram H. Gornicki P. Charles M. Just J. Huneau C. (2011). Duplication and partitioning in evolution and function of homoeologous Q loci governing domestication characters in polyploid wheat. 108 18737–18742. 10.1073/pnas.1110552108 22042872 PMC3219148

[B56] Zimin A. V. Puiu D. Hall R. Kingan S. Clavijo B. J. Salzberg S. L. (2017). The first near-complete assembly of the hexaploid bread wheat genome, *Triticum aestivum*. 6 1–7. 10.1093/gigascience/gix097 29069494 PMC5691383

