# Supplementary material for: Gene Duplication and Evolution Dynamics in the Homeologous Regions Harboring Multiple Prolamin and Resistance Gene Families in Hexaploid Wheat
Source: Front Plant Sci. 2018 May 23;9:673. doi: 10.3389/fpls.2018.00673 (PMC5974169; doi:10.3389/fpls.2018.00673)
Supplement: Supplementary file 1 [file Table_1.PDF]

Table S1. Annotation and colinearity of the orthologous prolamin and resistance gene regions from Chinese Spring A, B, and D genomes

Note: Ancestral genes are highlighted in blue. Large syntenic blocks between conserved ancestral genes are highlighted in yellow. in many cases, the colinearity among syntenic blocks is difficult to draw due to various sequence variations; therefore, orthologous genes may not be connected by alignment in the same rows. Putative prolamin and resistance pseudogenes are indicated with *

Annotation	Chinese Spring D			Chinese Spring B			Chinese Spring A		
	Gene symbol	Start	End	Gene symbol	Start	End	Gene symbol	Start	End
Putative LRR receptor-like serine/threonine-protein kinase (fragment)	RLK-D1*	2991	5478						
Charged multivesicular body protein 5		5817	9900						
Putative LRR receptor-like serine/threonine-protein kinase	RLK-D2	48964	52457						
Peptidase		72012	74481						
putative disease resistance protein RGA3 (fragment)				NLR-B1*	1	2378			
Disease resistance protein RPP13				NLR-B2	26937	30651			
Putative LRR receptor-like serine/threonine-protein kinase (Pseudo)				RLK-B1*	35115	38364			
Putative LRR receptor-like serine/threonine-protein kinase (fragment)				RLK-B2*	44549	48487			
NRC1a				NLR-B3	63044	67014			
Putative disease resistance RPP13-like protein 3 (Pseudo)				NLR-B4*	156500	160447			
Putative disease resistance RPP13-like protein 3 (fragment)				NLR-B5*	188482	190098			
putative disease resistance protein RGA3 (Pseudo)				NLR-B6*	245292	249847			
putative disease resistance RPP13-like protein 1 (fragment)				NLR-B7*	294009	295948			
Cysteine-rich receptor-like protein kinase 6					303536	305857			
putative disease resistance RPP13-like protein 1 (fragment)				NLR-B8*	316739	318927			
Cysteine-rich receptor-like protein kinase 6					326759	328483			
Putative disease resistance protein RGA4 (fragment)				NLR-B9*	339681	341139			
Cysteine-rich receptor-like protein kinase 6					349648	350995			
putative disease resistance RPP13-like protein 1 (Pseudo)				NLR-B10*	363856	373615			
uncharacterized protein LOC109742529					377233	386287			
putative late blight resistance protein homolog R1A-4				NLR-B11	490653	493705			
Putative disease resistance protein RGA4				NLR-B12	565241	570977			
calcium-transporting ATPase 1					584552	600499			
Snn1					617533	620777			
4-hydroxyphenylpyruvate dioxygenase					800351	818537			
putative alliin lyase					818631	820715			
putative disease resistance protein RGA3				NLR-B13	958960	963329			
receptor kinase-like protein Xa21				RLK-B3	1121048	1125033			
receptor kinase-like protein Xa21 (Pseudo)				RLK-B4	1372752	1375685			
MDIS1-interacting receptor like kinase 2-like (Pseudo)				RLK-B5*	1382414	1385600			
NRC1 motif deletion mutant 2, partial (fragment)				NLR-B14*	1391539	1393888			
hypothetical protein F775_23406					1398755	1407759			
myb-related protein P-like					1428134	1428964			
myb-related protein P-like					1440820	1444119			
hydroxycinnamoyltransferase 1-like					1496568	1499423			
hydroxycinnamoyltransferase 1-like					1516134	1517113			
D49Peptidase					1799252	1801185			
SNF1-related protein kinase regulatory subunit beta-3					1882372	1883165			
hypothetical protein TRIUR3_33392					2079783	2086432			
flavin-containing monooxygenase FMO GS-OX-like 9 isoform X2					2103861	2105682			
Putative disease resistance protein RGA3 (fragment)				NLR-B15*	2145179	2152301			
F-box/LRR-repeat protein 14-like					2157359	2159161			
Putative LRR receptor-like serine/threonine-protein kinase (Pseudo)				RLK-B6*	2163466	2166803			
PREDICTED: charged multivesicular body protein 5-like					2167286	2170279			
Putative LRR receptor-like serine/threonine-protein kinase				RLK-B7	2172237	2175585			
Putative LRR receptor-like serine/threonine-protein kinase (fragment)					2185365	2188822			
MDIS1-interacting receptor like kinase 2-like (fragment)				RLK-B9*	2233927	2235710			
Disease resistance protein RPM1 (repeat insertion)				NLR-B16*	2273199	2291483			
Wall-associated receptor kinase 5					2303159	2309233			
MDIS1-interacting receptor like kinase 2-like				RLK-B10	2314114	2317335			
F-box/LRR-repeat protein 14-like					2327801	2331178			
Putative LRR receptor-like serine/threonine-protein kinase (fragment)				RLK-B11*	2332350	2336297			
F-box/LRR-repeat protein 14-like					2362517	2364359			
Putative LRR receptor-like serine/threonine-protein kinase				RLK-B12	2368606	2372151			
D49Peptidase					2423038	2426249			
SNF1-related protein kinase regulatory subunit beta-3								1	1137
Disease resistance RPP8-like protein 3 (repeat insertion)							NLR-A1*	19726	50270
putative NBS-LRR resistance protein (fragment)							NLR-A2*	61282	64876
Disease resistance protein RPP13 (fragment)							NLR-A3*	98236	101878
AAA-ATPase ASD, mitochondrial-like								160133	161710
wall-associated receptor kinase 5-like								301766	307016
putative NBS-LRR resistance protein (fragment)							NLR-A4*	335244	337569
3'-N-debenzoyl-2'-deoxytaxol N-benzoyltransferase								374909	377238
transcription factor MYC2-like								461821	462427
root hair defective 3 GTP-binding protein-like								725627	732246
Disease resistance protein RPP13 (fragment)							NLR-A5*	834573	838720
Putative disease resistance protein RGA4 (fragment)							NLR-A6*	858926	866244
F-box/LRR-repeat protein 14-like								900975	902050
F-box/LRR-repeat protein 14-like								905047	907895
MDIS1-interacting receptor like kinase 2-like (fragment)							RLK-A1*	932930	934504
SNF								936646	938697
Putative LRR receptor-like serine/threonine-protein kinase							RLK-A2	940391	943770
SNF								944118	947606
F-box/LRR-repeat protein 14-like								970491	972377
ω-gliadin							ω-A1*	977427	978413
ω-gliadin protein							ω-A2*	1031825	1032968
ω-gliadin							ω-A3*	1051374	1052489
Exonuclease		187804	188733		2437837	2439135		1074189	1075501
hypothetical protein F775_23701		180229	186736		2439578	2443549		1077074	1078463
glutathione S-transferase T3-like								1173266	1174065

transcription initiation factor TFIID subunit 6		144199	151004		2536886	2544520		1213391	1222653
E3 ubiquitin-protein ligase ORTHRUS 2-like		124710	131834		2555958	2560392		1336950	1343886
Meiosis protein mei2		114649	121989		2562972	2568957		1345498	1352836
charged multivesicular body protein 5-like		82677	86218		2718236	2721885		1435373	1438688
Putative LRR receptor-like serine/threonine-protein kinase	RLK-D3	78335	82426	RLK-B13	2723418	2725951	RLK-A3	1440686	1443498
ω-gliadin	ω-D1	205184	206317						
SNF1-related protein kinase regulatory subunit beta-3		355763	356969						
Fatty acyl-CoA reductase 1		473227	474362						
uncharacterized protein LOC109769789		483074	484748						
cytokinin riboside 5'-monophosphate phosphoribohydrolase LOG6		692058	700877						
F-box/kelch-repeat protein At1g23390-like		1263420	1264339						
hypothetical protein F775_32967		1283591	1286954						
50S ribosomal protein L28, chloroplastic		1532056	1533264						
uncharacterized protein LOC109734558		1539457	1542456						
peroxisomal membrane protein PEX14-like isoform X3		1542773	1546312						
E3 ubiquitin-protein ligase SIS3		1546660	1550671						
uncharacterized protein LOC109734571 isoform X2		1553242	1557641						
hypothetical protein F775_30898		1567097	1568816						
proline-rich receptor-like protein kinase PERK8		1574218	1575211						
proline-rich receptor-like protein kinase PERK8		1575314	1577538						
hypothetical protein F775_18614		1578594	1582195						
putative phospholipid-transporting ATPase 9		1603783	1609847						
uncharacterized protein LOC109734557 isoform X1		1629048	1632480						
hypothetical protein F775_42529		1648577	1651273						
paired amphipathic helix protein Sin3-like 4		1683656	1694271						
Putative LRR receptor-like serine/threonine-protein kinase (fragment)	RLK-D4*	1892459	1893454						
wall-associated receptor kinase 5-like		1894699	1899227						
SnTox1 sensitivity protein		1918926	1921234						
hypothetical protein F775_20942		1928251	1931924						
putative NBS-LRR resistance protein	NLR-D1	1942544	1945963						
Lr21	NLR-D2	1953760	1957291						
hypothetical protein F775_04597		1972278	1977433						
histone acetyltransferase p300-like isoform X1		2001949	2003503						
hypothetical protein F775_17120		2052825	2055109						
uncharacterized protein At2g27730, mitochondrial-like		2135391	2136501						
Putative disease resistance protein RGA4 (Pseudo)	NLR-D3*	2147947	2151662						
Disease resistance RPP8-like protein 3 (fragment)	NLR-D4*	2179787	2182977						
Speckle-type POZ protein-like protein B		2197049	2199416						
hypothetical protein TRIUR3_16355		2323418	2326174						
Disease resistance protein RPP13 (Pseudo)	NLR-D5*	2350277	2353511						
ω-gliadin	ω-D2	2569605	2570762						
ω-gliadin	ω-D3	2583522	2584679						
Peptidase		2577230	2578395						
Charged multivesicular body protein 5		2598329	2599708						
Putative LRR receptor-like serine/threonine-protein kinase	RLK-D5	2600467	2604365						
Putative LRR receptor-like serine/threonine-protein kinase	RLK-D6	2612456	2615829						
Pentatricopeptide repeat protein		2633381	2634229						
probable L-gulonolactone oxidase 4		2644275	2647543						
SNF7 domain protein		2663188	2664488						
MDIS1-interacting receptor like kinase 2-like isoform X1	RLK-D7*	2666646	2669764						
Putative LRR receptor-like serine/threonine-protein kinase (fragment)				RLK-B14*	2749477	2753193			
F-box/LRR-repeat protein 14-like					2759622	2761082			
D49Peptidase					2764384	2766319			
Putative LRR receptor-like serine/threonine-protein kinase				RLK-B15	2771569	2777151			
F-box/LRR-repeat protein 14-like					2782248	2784056			
Putative LRR receptor-like serine/threonine-protein kinase				RLK-B16	2800549	2803955			
Putative LRR receptor-like serine/threonine-protein kinase (fragment)				RLK-B17*	2806994	2808899			
Putative LRR receptor-like serine/threonine-protein kinase (fragment)				RLK-B18*	2820825	2822339			
probable leucine-rich repeat receptor-like protein kinase (fragment)				RLK-B19*	2829997	2833428			
F-box/LRR-repeat protein 14-like					2835176	2839798			
EBNA-1-like protein					2848765	2856067			
Disease resistance protein RPM1 (repeat insertion)				NLR-B17*	2873918	2888153			
Putative LRR receptor-like serine/threonine-protein kinase				RLK-B20	3000864	3004126			
Wall-associated receptor kinase 5					3026681	3032742			
Peptidase					3052598	3055547			
MDIS1-interacting receptor like kinase 2-like (Pseudo)				RLK-B21*	3062214	3065998			
PREDICTED: receptor-like protein kinase 2 (fragment)				RLK-B22*	3075296	3080095			
Putative LRR receptor-like serine/threonine-protein kinase (Pseudo)				RLK-B23*	3093396	3096886			
disease susceptibility protein LOV1 (repeat insertion)				NLR-B18*	3103432	3118063			
disease resistance protein RPP13-like				NLR-B19	3128700	3138860			
Putative LRR receptor-like serine/threonine-protein kinase				RLK-B24	3147951	3149713			
F-box/LRR-repeat protein 14-like					3191370	3195364			
hypothetical protein TRIUR3_00483								1451375	1454463
Putative LRR receptor-like serine/threonine-protein kinase							RLK-A4	1477051	1480489
Putative LRR receptor-like serine/threonine-protein kinase							RLK-A5	1489147	1492439
F-box/LRR-repeat protein 14-like								1519958	1521886
Putative LRR receptor-like serine/threonine-protein kinase (fragment)							RLK-A6*	1565200	1569953
Putative disease resistance protein RGA1 (Pseudo)							NLR-A7*	1601311	1605157
Putative LRR receptor-like serine/threonine-protein kinase							RLK-A7	1639833	1643443
Putative LRR receptor-like serine/threonine-protein kinase							RLK-A8	1655048	1658429
Putative LRR receptor-like serine/threonine-protein kinase							RLK-A9	1663915	1667186
Putative LRR receptor-like serine/threonine-protein kinase (fragment)							RLK-A10*	1684493	1686304
AetG27								1711267	1712919
carotenoid 9,10(9',10')-cleavage dioxygenase-like								1740422	1744757
γ-gliadin				γ-B1	3205846	3206754			
γ-gliadin	γ-D1	2679925	2680833	γ-B2	3231931	3232824			
δ-gliadin	δ-D1	2720967	2721941	δ-B1	3243809	3244762	δ-A1*	1760690	1761589
δ-gliadin	δ-D2*	2733623	2734676				δ-A2*	1779285	1780176

Block 1

δ-gliadin	γ-D2	2738634	2739617	γ-B3*	3296693	3297757	γ-A1	1829536	1830708
γ-gliadin	γ-D3	2800548	2801435	γ-B4	3338800	3339906	γ-A2*	1893006	1894050
γ-gliadin	γ-D4	2809334	2810230	γ-B5*	3376470	3377504	γ-A3	2013589	2014627
γ-gliadin				γ-B6	3381440	3382315	γ-A4	2021309	2022347
cyclophilin-like protein		2837432	2839865		3396272	3399121		2027521	2028411
AI1	AI-D1	2840895	2841637	AI-B1	3400021	3400411	AI-A1	2029775	2030451
AI2	AI-D2	2846183	2846953	AI-B2	3414588	3415364	AI-A2	2033222	2033981
ω gliadin	ω-D4*	2871351	2872513	ω-B1*	3502805	3504114			
ω gliadin	ω-D5*	2883837	2885001	ω-B2*	3544802	3546024			
ω gliadin	ω-D6*	2915026	2916538	ω-B3	3572262	3573497			
ω gliadin	ω-D7*	2922170	2922499	ω-B4*	3584545	3585843		Block 2	
ω gliadin				ω-B5*	3596906	3598222			
ω gliadin,				ω-B6	3608287	3609621			
ω gliadin,				ω-B7*	3653082	3654432			
ω gliadin,				ω-B8*	3663027	3664482			
PREDICTED: uncharacterized protein LOC104582784		2942902	2944368		3736624	3737219		2041896	2043337
LMW-GS P-31	LMW-D1	2955499	2956563	LMW-B1*	3746287	3747351	LMW-A1*	2075119	2075568
LMW-GS P-31	LMW-D2	2970318	2971241	LMW-B2	3811503	3812615	LMW-A2	2180811	2181941
LMW-GS P-31				LMW-B3	3912124	3913203			
Putative disease resistance protein RGA4	NLR-D6	2978561	2982601						
ARM REPEAT PROTEIN INTERACTING WITH ABF2-like		3008626	3011346						
truncated powdery mildew resistance protein Pm3 (fragment)	NLR-D7*	3109025	3110862						
Putative disease resistance protein RGA4 (Pseudo)	NLR-D8*	3138272	3143174						
hypothetical protein F775_17960		3187683	3189324						
Disease resistance protein RPP13	NLR-D9	3380954	3385673						
hypothetical protein F775_11559		3390044	3392619						
SWIM Zn-finger protein		3393149	3394247						
methylsterol monooxygenase 1-1-like		3426058	3428332						
probable flavin-containing monooxygenase 1		3432576	3434592						
hypothetical protein F775_15043		3467498	3468528						
Cytochrome P450 71C4		3472484	3483391						
dirigent protein 15-like		3540175	3541412						
hypothetical protein F775_17246		3547619	3548333						
myrosinase-binding protein 2-like		3572293	3574444						
low-molecular-weight glutenin subunit group 8 type IV	LMW-D3	3667220	3668116					Block 3	
hypothetical protein F775_22394		3694144	3695695						
low molecular weight glutenin subunit LMW-8	LMW-D4*	3781377	3782276						
Putative disease resistance protein RGA4				NLR-B20	3920509	3924961			
Pm3b-like disease resistance protein (Pseudo)				NLR-B21*	3970782	3973568			
Pm3-like disease resistance protein (fragment)				NLR-B22*	3980822	3983576			
predicted protein [Hordeum vulgare subsp. vulgare]					3984266	3985324			
PM3-1B				NLR-B23	4076134	4080660			
putative disease resistance protein RGA4 isoform X1 (fragment)				NLR-B24*	4159214	4163453			
Pm3b-like disease resistance protein 2Q9				NLR-B25	4173361	4179332			
putative disease resistance protein RGA4				NLR-B26	4209262	4213761			
Pm3b-like disease resistance protein 2Q9				NLR-B27	4283247	4287689			
PREDICTED: putative disease resistance RPP13-like protein 3				NLR-B28	4292459	4295620			
PREDICTED: disease resistance protein RPP13-like				NLR-B29	4322047	4325499			
Disease resistance protein RPM1				NLR-B30	4334197	4336594			
putative disease resistance protein RGA4 isoform X1 (Pseudo)				NLR-B31*	4417285	4421560			
Pm3b-like disease resistance protein 2Q9 (Pseudo)				NLR-B32*	4503361	4507446			
dirigent protein 15-like					4533169	4534306			
myrosinase-binding protein 2-like					4547825	4550101			
myrosinase-binding protein 2-like					4561938	4563525			
dehydrogenase Adh1,alcohol					4611467	4613683			
putative disease resistance protein RGA4 (fragment)				NLR-B33*	4625435	4628148			
keratin-associated protein 9-1-like					4646783	4647425			
protein FAR1-RELATED SEQUENCE					4650676	4654539			
low molecular weight glutenin subunit				LMW-B4	4656638	4657690			
dirigent protein 15-like					4663850	4665145			
Pm3-like protein (fragment)							NLR-A8*	2192039	2194784
powdery mildew resistance protein PM3CS							NLR-A9	2545608	2550049
Serine/threonine-protein kinase								2550263	2551616
powdery mildew resistance protein PM3 variant (fragment)							NLR-A10*	2552270	2553984
Putative disease resistance protein RGA4							NLR-A11	2597456	2602141
disease resistance protein RPM1-like (fragment)							NLR-A12*	2703359	2705597
2'-deoxymugineic-acid 2'-dioxygenase-like								2843807	2845039
(+)-δ-cadinene synthase isozyme XC14								3061919	3065800
protein DJ-1 homolog A-like					4690017	4691946		3569861	3571925
Ankyrin-1		3912157	3918052		4693203	4698739		3597941	3601610
hypothetical protein F775_31607		3919482	3920951		4700625	4701938		3605627	3607359
Pm3b-like disease resistance protein 2Q9	NLR-D10	3921836	3926064						
Cw7 protein, putative		4008589	4011787						
leucine zipper putative tumor suppressor 2-like		4030186	4032293						
disease resistance protein RPM1 (repeat insertion)	NLR-D11*	4130101	4139286						
putative disease resistance RPP13-like protein 3 (fragment)	NLR-D12*	4143406	4145432						
putative disease resistance protein RGA4 (fragment)	NLR-D13*	4148664	4151762						
low molecular weight glutenin	LMW-D5*	4290584	4291696						
uncharacterized protein LOC8071902		4331731	4333416						
hypothetical protein F775_24341		4443898	4445004						
dirigent protein 15-like		4445007	4449276						
methylsterol monooxygenase 1-1-like		4577062	4578234						
predicted protein		4742310	4748827						
putative disease resistance protein RGA4 (fragment)	NLR-D14*	4761591	4764196						
powdery mildew resistance protein PM3b (fragment)	NLR-D15*	4787437	4789036						
powdery mildew resistance protein PM3 variant (Pseudo)	NLR-D16*	4793859	4800055						
putative disease resistance protein RGA4 (fragment)	NLR-D17*	4848579	4850188						
truncated powdery mildew resistance protein Pm3	NLR-D18	4886535	4889030						

Block 4

hypothetical protein TRIUR3_26627		4908585	4912214						
Putative disease resistance protein RGA4 (Pseudo)	NLR-D19*	5047263	5051479						
LMW-GS P-11	LMW-D6	5055321	5056531						
putrescine hydroxycinnamoyltransferase 3-like		5158675	5160186						
anthranilate N-benzoyltransferase protein 1-like		5165870	5167254						
acetylserotonin O-methyltransferase 1-like		5205486	5206950						
Lectin-domain containing receptor kinase A4.3		5233130	5234712						
protein DETOXIFICATION 12-like		5258182	5263132						
Serine/threonine-protein kinase		5268294	5269052						
Pm3b-like disease resistance protein				NLR-B34	4702594	4707059			
dirigent protein 15-like					4721624	4722584			
myrosinase-binding protein 2-like					4907067	4908660			
anthranilate N-benzoyltransferase protein 1-like					4959685	4961079			
Pm3b, partial (fragment)				NLR-B35*	5010804	5012961			
glycine-rich cell wall structural protein-like					5041794	5044386			
Pm3-like disease resistance protein (fragment)				NLR-B36	5045182	5047979			
Pm3				NLR-B37	5058942	5061969			
disease resistance protein RPM1-like (fragment)				NLR-B38*	5229544	5236242			
putative disease resistance RPP13-like protein 3				NLR-B39	5240436	5244583			
powder mildew resistance-like protein (fragment)				NLR-B40*	5260981	5264362			
myrosinase-binding protein 2-like					5323879	5325744			
dirigent protein 15-like					5339212	5340382			
dirigent protein 15-like					5378998	5380168			
putative disease resistance protein RGA4				NLR-B41	5446630	5451058			
putative nuclease HARBI1					5648652	5650562			
Pm3b-like disease resistance protein (Pseudo)				NLR-B42*	5721730	5726145			
Pm3b-like disease resistance protein (repeat insertion)				NLR-B43*	5757335	5773577			
Cytochrome P450 71C4					5888230	5891424			
putative flavin-containing monooxygenase 1					5894174	5902112			
Putative disease resistance protein RGA4				NLR-B44	5911798	5916147			
Auxin response factor 13					6154850	6159547			
myrosinase-binding protein 2-like					6206569	6208818			
Glycoside hydrolase					6268590	6275051			
putative disease resistance protein RGA4 isoform X2				NLR-B45	6277584	6281893			
PM3-1B (fragment)				NLR-B46*	6292839	6293566			
putrescine hydroxycinnamoyltransferase 3-like					6339466	6342155			
acetylserotonin O-methyltransferase 1-like					6393673	6395288			
protein DETOXIFICATION 12-like					6420829	6422242			
powdery mildew resistance protein PM3 variant (fragment)							NLR-A13*	3607196	3609231
powdery mildew resistance protein PM3 variant (fragment)							NLR-A14*	3611451	3613486
powdery mildew resistance protein PM3 variant (fragment)							NLR-A15*	3615706	3617741
powdery mildew resistance protein PM3 variant (fragment)							NLR-A16*	3620248	3621997
powdery mildew resistance protein PM3 variant (Pseudo)							NLR-A17*	3624577	3628334
powdery mildew resistance protein PM3A (Pseudo)							NLR-A18*	3644606	3648891
powdery mildew resistance protein PM3b							NLR-A19	3773009	3777079
receptor kinase-like protein Xa21							RLK-A11	3980607	3984029
powdery mildew resistance protein PM3b (fragment)							NLR-A20*	4116932	4121168
hypothetical protein F775_28772								4130261	4132083
stripe rust resistance protein YR10 (Pseudo)							NLR-A21*	4260712	4264071
Pm3b-like disease resistance protein (fragment)							NLR-A22*	4401276	4402036
Serine/threonine-protein kinase								4437142	4441661
Putative disease resistance protein RGA4							NLR-A23	4443465	4447909
cellulose synthase-like protein								4527268	4529507
dirigent protein 15-like								4609605	4615317
low-molecular-weight glutenin subunit group 7 type IV	LMW-D7	5271184	5272095				LMW-A3*	4617350	4621694
low-molecular-weight glutenin subunit, partial							LMW-A4*	4711696	4712740
Ribosomal protein S19, mitochondrial								4715816	4717213
serine/threonine-protein kinase 19 isoform X2		5321218	5325288		6445879	6452724		4719935	4724609
wall-associated receptor kinase 3-like		5336724	5340756					5034476	5038208
wall-associated receptor kinase 3-like								5048985	5053821
wall-associated receptor kinase 3-like								5064491	5066114
predicted protein		5341513	5344949					4934028	4938927
RING-H2 finger protein ATL70-like		5355104	5360433					4918899	4920637
mitochondrial L2 ribosomal protein		5363246	5365496						
hypothetical protein F775_26572		5367484	5368444		6456142	6457823		4912360	4912978
Hypothetical protein OsI_18100		5368850	5374639		6468476	6472011		4906841	4910939
serine/threonine-protein kinase 19 isoform X4					6496876	6498036			
F-box protein								5257071	5258971
transforming growth factor-beta receptor-associated protein 1		5425256	5432378		6523392	6529506		5319870	5328908
polygalacturonase inhibitor-like		5432393	5434849		6529791	6531402		5329377	5331543
G-type lectin S-receptor-like serine/threonine-protein kinase At2g19130		5434877	5438518		6533955	6535908		5332659	5335019
