# Supplementary material for: Gene Duplication and Evolution Dynamics in the Homeologous Regions Harboring Multiple Prolamin and Resistance Gene Families in Hexaploid Wheat
Source: Front Plant Sci. 2018 May 23;9:673. doi: 10.3389/fpls.2018.00673 (PMC5974169; doi:10.3389/fpls.2018.00673)
Supplement: Supplementary file 2 [file Table_2.PDF]

Table S2. Annotation and colinearity in the orthologous prolamin and resistance regions from *Ae. tauschii* and Chinese Spring D genomes.

Note: Ancestral genes that are also shared with rice, Brachypodium, and sorghum are highligted with light blue. Genes that are not located in the five large sturcutral vaiation region, but also not shared by the two D genomes are highlighted with red. Regions representing large structural variations are highligted with blue. Different groups of prolamins are also shown in difference color

Annotation	Ae. tauschii Gene ID	Gene Symbol	AETstart	AET end	CSD Gene ID	Gene Symbol	Start	End	Brachypodium	Rice	Sorghum
Putative Protein	1		1	430							
LRR-Kinase	2	RLK-D^t1	189880	202743			Gap 1				
NBS-LRR (Lr21-1)	3	NLR-D^t1	250780	254559							
LRR receptor-like serine/threonine-protein kinase					1	RLK-D1	2991	5478			
Charged multivesicular body protein 5					2		5817	9900			
LRR receptor-like serine/threonine-protein kinase					3		48964	52457			
Peptidase					4	RLK-D2	72012	74481			
LRR-Kinase	4	RLK-D^t2	280662	283063	5	RLK-D3	78335	82426			
SNF7 domain protein	5		284421	287303	6		82677	86218	Bradi2g39980.1	LOC_Os05g01250.1	Sb09g000340.1
Nucletide binding protein	6		325829	330029	7		114649	121989	Bradi2g39990.1	LOC_Os05g01240.4	Sb09g000330.1
E3 ubiquitin ligase	7		333401	336291	8		124710	131834	Bradi2g40000.1	LOC_Os05g01230.1	Sb09g000320.1
Transcription initiation factor	8		351576	357403	9		144199	151004			
Hypothetical protein	9		417131	419053	10		180229	186736	Bradi2g40010.1	LOC_Os05g01210.2	Sb09g000310.1
Exonuclease	10		420923	421846	11		187804	188733	Bradi2g40020.1	LOC_Os05g01200.1	Sb09g000300.1
ω-gliadin	11	ω-D^t1	438700	439714	12	ω-D1	205184	206317			
SNF1-related protein kinase regulatory subunit beta-3			Gap 2		13		355763	356969			
Fatty acyl-CoA reductase 1					14		473227	474362			
uncharacterized protein LOC109769789					15		483074	484748			
tokinin riboside 5'-monophosphate phosphoribohydrolase LOG6					16		692058	700877			
F-box/kelch-repeat protein At1g23390-like					17		1263420	1264339			
hypothetical protein F775_32967					18		1283591	1286954			
50S ribosomal protein L28, chloroplastic					19		1532056	1533264			
uncharacterized protein LOC109734558					20		1539457	1542456			
peroxisomal membrane protein PEX14-like isoform X3					21		1542773	1546312			
E3 ubiquitin-protein ligase SIS3					22		1546660	1550671			
uncharacterized protein LOC109734571 isoform X2					23		1553242	1557641			
hypothetical protein F775_30898					24		1567097	1568816			
proline-rich receptor-like protein kinase PERK8					25		1574218	1575211			
proline-rich receptor-like protein kinase PERK8					26	RLK-D4	1575314	1577538			
hypothetical protein F775_18614					27		1578594	1582195			
putative phospholipid-transporting ATPase 9					28		1603783	1609847			

uncharacterized protein LOC109734557 isoform X1					29		1629048	1632480			
hypothetical protein F775_42529					30		1648577	1651273			
paired amphipathic helix protein Sin3-like 4					31		1683656	1694271			
Putative LRR receptor-like serine/threonine-protein kinase					32		1892459	1893454			
wall-associated receptor kinase 5-like					33		1894699	1899227			
SnTox1 sensitivity protein					34		1918926	1921234			
hypothetical protein F775_20942					35		1928251	1931924			
putative NBS-LRR resistance protein					36	NLR-D1	1942544	1945963			
Lr21					37	NLR-D2	1953760	1957291			
hypothetical protein F775_04597					38		1972278	1977433			
histone acetyltransferase p300-like isoform X1					39		2001949	2003503			
hypothetical protein F775_17120					40		2052825	2055109			
uncharacterized protein At2g27730, mitochondrial-like					41		2135391	2136501			
Putative disease resistance protein RGA4					42	NLR-D3	2147947	2151662			
Disease resistance RPP8-like protein 3					43	NLR-D4	2179787	2182977			
Speckle-type POZ protein-like protein B					44		2197049	2199416			
hypothetical protein TRIUR3_16355					45		2323418	2326174			
Disease resistance protein RPP13					46	NLR-D5	2350277	2353511			
ω-gliadin	12	ω-D^{t}2	459426	460574	47	ω-D2	2569605	2570762			
Omega-D3					48	ω-D3	2583522	2584679			
Peptidase	13		473741	474408	49		2577230	2578395			
SNF7 domain protein	14		480090	480926	50		2598329	2599708			
LRR-Kinase	16	RLK-D^{t}3	487486	491207	51	RLK-D5	2600467	2604365			
LRR-Kinase	17	RLK-D^{t}4	498879	502299	52	RLK-D6	2612456	2615829			
LRR-Kinase	18	RLK-D^{t}5	518182	521314			Gap 3				
LRR-Kinase	19	RLK-D^{t}6	525015	528468							
LRR-Kinase	20	RLK-D^{t}7	540053	543642							
Peptidase	21		546337	548035							
LRR-Kinase	22	RLK-D^{t}8	591141	596176							
NBS-LRR (Lr21-2)	23	NLR-Dt2	618688	622704							
LRR-Kinase	24	RLK-D^{t}9	631846	634932							
LRR-Kinase	25	RLK-D^{t}10	672119	675527							
NBS-LRR (RPM1)	26	NLR-Dt3	740553	742925							
Mov34/MPN/PAD-1 family protein	27		754865	756886							
LRR-Kinase	28	RLK-D^{t}11	759116	761042							
SNF7 domain protein	29		777052	777926							
LRR-Kinase	30	RLK-D^{t}12	779010	782399							
SNF7 domain protein	31		784829	785839							
LRR-Kinase	32	RLK-D^{t}13	786959	790289							
LRR-Kinase	33	RLK-D^{t}14	841576	844908							

LRR-Kinase	34	RLK-D^{t}15	859635	862627							
Pentatricopeptide repeat protein	35		865769	866707	53		2633381	2634229			
L-gulonolactone oxidase	36		872648	874999	54		2644275	2647543			
SNF7 domain protein	37		890589	891889	55		2663188	2664488			
LRR-Kinase	38	RLK-D^{t}16	894048	897247	56	RLK-D7	2666646	2669764			
γ-gliadin	39	γ-D^{t}1	907416	908324	57	γ-D1	2679925	2680833			
δ-gliadin	40	δ-D^{t}1	948015	948992	58	δ-D1	2720967	2721941			
δ-gliadin	41	δ-D^{t}2	969708	969986	59	δ-D2	2733623	2734676			
γ-gliadin	42	γ-D^{t}2	974546	975538	60	γ-D2	2738634	2739617			
γ-gliadin	43	γ-D^{t}3	1008548	1009411	61	γ-D3	2800548	2801435			
γ-gliadin	44	γ-D^{t}4	1015678	1016568	62	γ-D4	2809334	2810230			
Cyclophilin-like protein	45		1034221	1037685	63		2837432	2839865	Bradi2g39950.1	LOC_Os05g01270.1	Sb09g000350.1
α-amylase inhibitor	46	AI-D^{t}1	1037686	1038428	64	AI-D1	2840895	2841637			
α-amylase inhibitor	47	AI-Dt2	1043061	1043666	65	AI-D2	2846183	2846953	Bradi2g39940.1		
ω-gliadin	48	ω-D^{t}3	1061162	1062306	66	ω-D4	2871351	2872513			
ω-gliadin	49	ω-D^{t}4	1074063	1074794	67	ω-D5	2883837	2885001			
ω-gliadin	50	ω-D^{t}5	1105011	1106249	68	ω-D6	2915026	2916538			
ω-gliadin	51	ω-D^{t}6	1111883	1112212	69	ω-D7	2922170	2922499			
Expressed protein	52		1133680	1133889	70		2942902	2944368	Bradi2g39930.1	LOC_Os05g01290.1	Sb09g000370.1
LMW-glutenin	53	LMW-D^{t}1	1143541	1144605	71	LMW-D1	2955499	2956563	Bradi2g39920.1		
LMW-glutenin	54	LMW-D^{t}2	1158348	1159265	72	LMW-D2	2970318	2971241	Bradi2g39910.1		
NBS-LRR (PM3-1)	55	NLR-D^{t}4	1167049	1169605	73	NLR-D6	2978561	2982601			
ARM REPEAT PROTEIN INTERACTING WITH ABF2-like					74		3008626	3011346			
truncated powdery mildew resistance protein Pm3					75	NLR-D7	3109025	3110862			
Pm3-like short fragment	56		1176790	1176356			Gap 4				
EBNA-1-like protein	57		1201445	1208803							
Hypothetical protein, expressed	58		1210068	1210754							
Werner Syndrome-like exonuclease-like	59		1225104	1225822							
Cw7 protein, putative	60		1310858	1314056							
A-kinase anchor protein 9 isoform	61		1315014	1316225							
E3 ubiquitin ligase	62		1333088	1333947							
Hypothetical protein OsI_30800	63		1395368	1397433							
NBS-LRR (PM3-4)	64	NLR-D^{t}8	1665799	1670193							
NBS-LRR (PM3-2)	65	NLR-D^{t}5	1727327	1732229	76	NLR-D8	3138272	3143174			
jasmonate-induced protein	66		1770165	1771446	77		3187683	3189324			
Werner syndrome ATP-dependent helicase	67		1903040	1904110							
NBS-LRR (RPP13)	68	NLR-D^{t}6	1961429	1966130	78	NLR-D9	3380954	3385673	Bradi2g39850		
respiratory burst oxidase-like protein J	69		1970908	1973049	79		3390044	3392619			
SWIM Zn-finger protein	70		1973580	1974677	80		3393149	3394247			
Methylsterol monooxygenase	71		2021275	2022894	81		3426058	3428332			

Flavin-containing monooxygenase	72		2027163	2029782	82		3432576	3434592			
Flavin-containing monooxygenase	73		2047992	2049282	83		3467498	3468528			
Cytochrome P450	74		2054952	2058120	84		3472484	3483391			
JA-induced protein	75		2122950	2125755	85		3540175	3541412			
JA-induced protein	76		2132035	2132741	86		3547619	3548333			
JA-induced protein	77		2157225	2158476	87		3572293	3574444			
LMW-glutenin	78	LMW-D^{t}3	2240444	2241301	88	LMW-D3	3667220	3668116			
JA-induced protein	79		2307940	2308319	89		3694144	3695695			
low molecular weight glutenin subunit LMW-8					90	LMW-D4	3781377	3782276			
Ankyrin-1 [Aegilops tauschii]	80		2369946	2373597	91		3912157	3918052	Bradi2g39890.1	LOC_Os05g01310.1	Sb09g000390.1
hypothetical protein F775_31607	81		2377519	2378067	92		3919482	3920951	Bradi2g39900.1	LOC_Os05g01300.1	Sb09g000380.1
NBS-LRR (PM3-3)	82	NLR-D^{t}7	2379232	2383669	93	NLR-D10	3921836	3926064			
uncharacterized protein KIAA0930 homolog			Gap 5		94		4008589	4011787			
leucine zipper putative tumor suppressor 2-like					95		4030186	4032293			
disease resistance protein RPM1					96	NLR-D11	4130101	4139286			
putative disease resistance RPP13-like protein 3					97	NLR-D12	4143406	4145432			
putative disease resistance protein RGA4					98	NLR-D13	4148664	4151762			
low molecular weight glutenin					99	LMW-D5	4290584	4291696			
uncharacterized protein LOC8071902					100		4331731	4333416			
dirigent protein 15-like					101		4443898	4445004			
hypothetical protein F775_24341					102		4445007	4449276			
methylsterol monooxygenase 1-1-like					103		4577062	4578234			
predicted protein					104		4742310	4748827			
putative disease resistance protein RGA4					105	NLR-D14	4761591	4764196			
powdery mildew resistance protein PM3b					106	NLR-D15	4787437	4789036			
predicted protein					107		4793859	4800055			
Putative disease resistance protein RGA4					108	NLR-D16	4848579	4850188			
truncated powdery mildew resistance protein Pm3					109	NLR-D17	4886535	4889030			
hypothetical protein TRIUR3_26627					110		4908585	4912214			
predicted protein	83		2429720	2430088							
NBS-LRR (PM3-5)	84	NLR-D^{t}9	2490358	2494792	111	NLR-D18	5047263	5051479			
LMW-glutenin	85	LMW-D^{t}4	2499673	2500722	112	LMW-D6	5055321	5056531			
Anthranilate N-benzoyltransferase protein 1	86		2593241	2594635	113		5158675	5160186			
Anthranilate N-benzoyltransferase protein 1	87		2601233	2602618	114		5165870	5167254			
O-methyltransferase-like protein	88		2640946	2642227	115		5205486	5206950			
Lectin-domain containing receptor kinase A4.3	89		2670052	2668470	116		5233130	5234712			
MATE efflux family protein	90		2695076	2699854	117		5258182	5263132			
Serine/threonine-protein kinase	91		2705237	2705994	118		5268294	5269052			
LMW-glutenin	92	LMW-D^{t}5	2708173	2709093	119	LMW-D7	5271184	5272095			
Serine/threonine-protein kinase	93		2728899	2735537	120		5321218	5325288			

wall-associated kinase 2	94		2744468	2747970	121		5336724	5340756			
Formin-like protein	95		2749201	2753458	122		5341513	5344949	Bradi2g39860.1		Sb09g000410.1
RING-H2 finger protein	96		2780707	2781231	123		5355104	5360433			
Mitochondrial L2 ribosomal protein	97		2782122	2786580	124		5363246	5365496			
Formin-like protein	98		2789643	2789849	125		5367484	5368444	Bradi2g39870.1	LOC_Os05g01330.1	
Hypothetical protein OsI_18100	99		2790710	2795984	126		5368850	5374639	Bradi2g39880.2	LOC_Os05g01320.1	Sb09g000400.1
Wall-associated kinase 2	100		2814339	2811192							
Growth factor-associated protein	101		2835794	2829820	127		5425256	5432378	Bradi2g39840.1	LOC_Os05g01360.1	Sb09g000420.1
Polygalacturonase inhibitor	102		2837796	2836783	128		5432393	5434849	Bradi2g39830.1	LOC_Os05g01370.1	Sb09g000440
Lectin receptor-like protein kinase	103		2850514	2853071	129		5434877	5438518			
