# Supplementary material for: Gene Duplication and Evolution Dynamics in the Homeologous Regions Harboring Multiple Prolamin and Resistance Gene Families in Hexaploid Wheat
Source: Front Plant Sci. 2018 May 23;9:673. doi: 10.3389/fpls.2018.00673 (PMC5974169; doi:10.3389/fpls.2018.00673)
Supplement: Supplementary file 3 [file Table_3.PDF]

Table S3. Estimation of prolamin transcript abundances in different developmental stages in Chinese Spring

		10WE		20SE		30SE	
		mapped reads	% gene reads	mapped reads	% gene reads	mapped reads	% gene reads
delta	A	7137	0.00%	4490	0.00%	2140	0.00%
	B	163204	0.11%	41190	0.03%	39354	0.03%
	D	361067	0.24%	490987	0.32%	434804	0.28%
	Total	531408	0.35%	536667	0.35%	476298	0.31%
gamma	A	5427922	3.59%	5342578	3.50%	5423923	3.55%
	B	8448585	5.58%	13334354	8.75%	15013019	9.83%
	D	8120195	5.36%	8892039	5.83%	10961726	7.17%
	Total	21996702	14.53%	27568971	18.08%	31398668	20.55%
omega	A	1571951	1.04%	1431771	0.94%	1189753	0.78%
	B	4101121	2.71%	3582749	2.35%	3018575	1.98%
	D	7450934	4.92%	7233931	4.75%	9437461	6.18%
	Total	13124006	8.67%	12248451	8.03%	13645789	8.93%
alpha	A	3766569	2.49%	7918698	5.19%	7538404	4.93%
	B	4778905	3.16%	14083289	9.24%	12195241	7.98%
	D	4846752	3.20%	9589118	6.29%	9894724	6.48%
	Total	13392226	8.85%	31591105	20.72%	29628369	19.39%
LMW	A	1883080	1.24%	4190657	2.75%	3624393	2.37%
	B	5248074	3.47%	10800748	7.09%	11084241	7.25%
	D	6148573	4.06%	10208473	6.70%	10092308	6.61%
	Total	13279727	8.77%	25199878	16.53%	24800942	16.23%
HMW	A	27048	0.02%	34396	0.02%	23462	0.02%
	B	3319999	2.19%	6622124	4.34%	6857098	4.49%
	D	4353874	2.88%	7162466	4.70%	5126568	3.36%
	Total	7700921	5.09%	13818986	9.07%	12007128	7.86%

Note: **10WE** - whole endosperm tissue 10 days post-anthesis, **20SE** - starchy endosperm tissue 20 days post-anthesis, **30SE** - stachy endosperm tissue 30 days post anthesis. Transcriptome data was downloaded from published data deposited in NCBI (Marcussen et al., 2014). Transcriptome reads were mapped to the annotated Chinese Spring cds dateset plus manually annotated prolamin genes. The abundance of each prolamin group (% gene reads) from different genomes was calculated by total reads mapped to the specific group divided by the total reads mapped to the complete set of wheat Chinese Spring gene coding regions.
