# Supplementary material for: Gene Duplication and Evolution Dynamics in the Homeologous Regions Harboring Multiple Prolamin and Resistance Gene Families in Hexaploid Wheat
Source: Front Plant Sci. 2018 May 23;9:673. doi: 10.3389/fpls.2018.00673 (PMC5974169; doi:10.3389/fpls.2018.00673)
Supplement: Supplementary file 4 [file Table_4.PDF]

Table S4: Alignment of Chinese Spring ω-gliadin genes to the TGAC Chinese Spring genome assembly

Gene	Gene length (bp)	TGAC Seq Scaffold ID	Length n(bp)	Match size	Identity%	Gene start	Gene end	TGAC start	TGAC end	E-value	Comment
ω-A1	987	TGACv1_scaffold_019727_1AS	78307	987	100	1	987	32042	33028	0	Perfect match
ω-A2	1080	no hit									
ω-A3	1116	TGACv1_scaffold_019336_1AS	119466	728	100	604	1116	39704	40019	0	Gap (N) in the middle
ω-A4	1118	TGACv1_scaffold_019220_1AS	142195	1118	100	1	1118	109763	108646	0	perfect match
ω-B1	1309	TGACv1_scaffold_050405_1BS	67355	928	97.41	389	1309	35225	36149	0	Partial, shift
ω-B2	1224	TGACv1_scaffold_053921_1BS	3105	288	100	937	1224	1	288	6.00E-148	partial, end of sequence
ω-B3	1236	TGACv1_scaffold_726552_1BS	545	217	100	1	217	329	545	2.00E-108	partial, end of sequence
ω-B4	1299	TGACv1_scaffold_054043_1BS	2970	447	100	1	447	2524	2970	0	partial, end of sequence
ω-B5	1317	TGACv1_scaffold_726552_1BS	545	217	100	1	217	329	545	2.00E-108	partial, end of sequence
ω-B6	1335	TGACv1_scaffold_058645_1BS	964	649	99.69	1	649	649	1	0	partial, end of sequence
ω-B7	1368	TGACv1_scaffold_055162_1BS	2125	177	100	1192	1368	2125	1949	3.00E-86	partial, end of sequence
ω-B8	1457	TGACv1_scaffold_050741_1BS	50759	863	93.28	1	844	24161	25016	0	Partial, shift
ω-D1	1134	TGACv1_scaffold_080437_1DS	96687	792	96.59	1	788	90724	91510	0	Partial, shift
ω-D2	1158	TGACv1_scaffold_640817_U	292782	1158	100	1	1158	40439	39282	0	perfect match
ω-D3	1158	TGACv1_scaffold_640817_U	292782	1158	100	1	1158	40439	39282	0	perfect match
ω-D4	1163	TGACv1_scaffold_081584_1DS	31752	427	100	737	1163	7776	7350	0	Gap (N) in the middle
ω-D5	1165	no hit									
ω-D6	1513	TGACv1_scaffold_080539_1DS	85265	535	99.63	1	1513	77404	78290	1.00E-135	Gap (N) in the middle
ω-D7	330	TGACv1_scaffold_080539_1DS	85265	330	100	1	330	71443	71772	7.00E-172	perfect match

Note: When the 19 ω-gliadin genes were used in a BLASTN search against the published CS draft genome sequence with Illimina shotgun reads, only four scaffolds contained sequences that have perfect matches to five ω-gliadin gene sequences (ω-A1, ω-A4, ω-D2, ω-D3, and ω-D7). The rest of ω-gliadin genes in the PacBio assembly were only partially aligned due to the present of gaps represented by Ns in the coding regions or missing sequences in the CS draft genome sequence database, suggesting that short reads might have issues in resolving complicated ω-gliadin gene sequences as compared to the PacBio long reads.
