# Supplementary material for: Gene Duplication and Evolution Dynamics in the Homeologous Regions Harboring Multiple Prolamin and Resistance Gene Families in Hexaploid Wheat
Source: Front Plant Sci. 2018 May 23;9:673. doi: 10.3389/fpls.2018.00673 (PMC5974169; doi:10.3389/fpls.2018.00673)
Supplement: Supplementary file 5 [file Image_1.pdf]

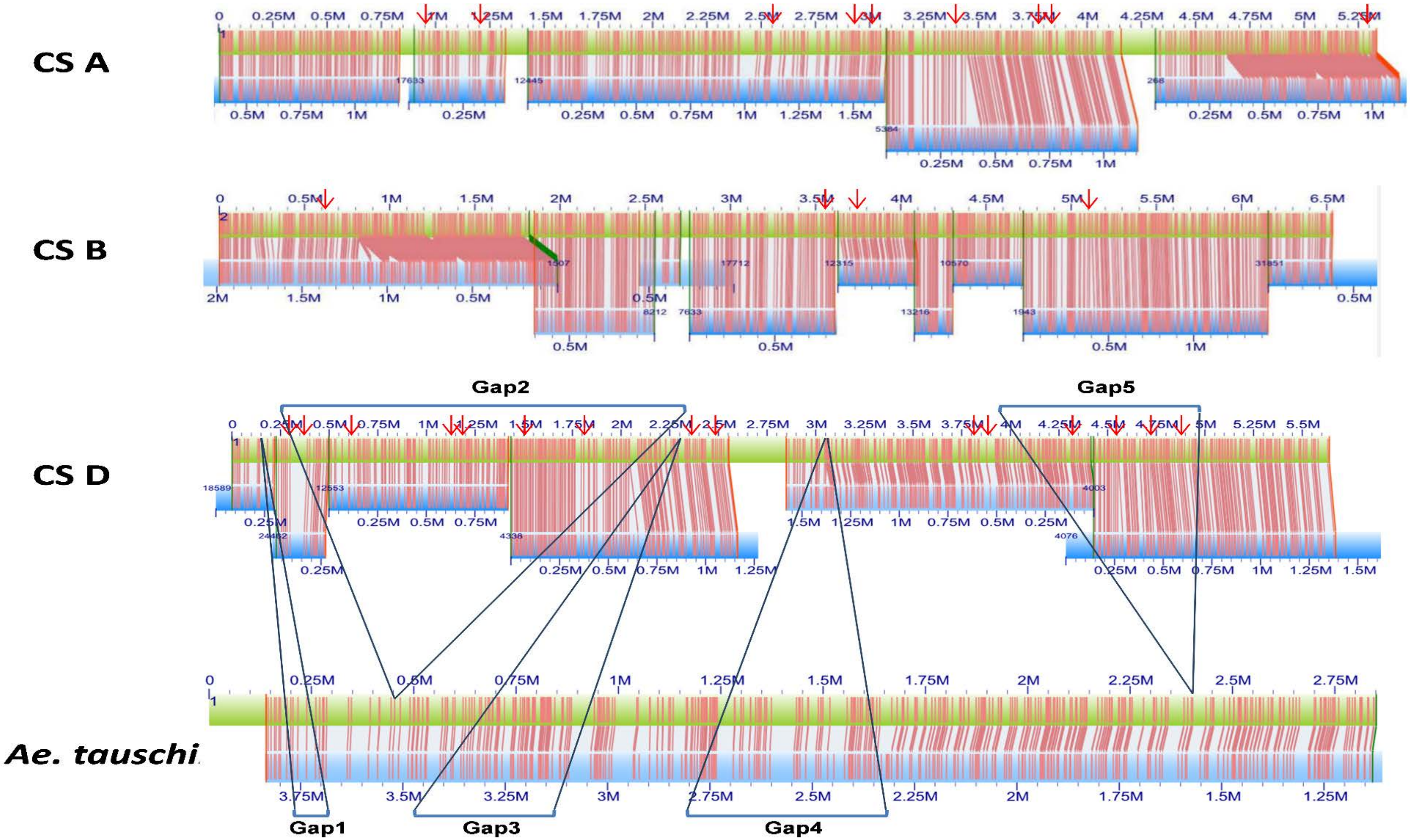

Figure S1. BioNano genome map alignments with the sequence assemblies of genomic regions harboring the prolamin and resistance gene families. BioNano optical maps and sequence assemblies of the Chinese Spring prolamin regions are described in the manuscript text. The alignments of the optical maps and assembled sequences was based on information of endonuclease, Nt.BspQ1, sites from the optical map and *in silico* digestion of the sequences (see Materials and Methods). Vertical lines represent agreements of sequence motifs of the endonuclease restriction site (GCTTCTTC) between the consensus sequences and BioNano map contigs. Red arrows indicate gap positions in the sequences. The *Ae. tauschii* region contains no gap in the sequence (gaps have been filled by PacBio sequences) and covered by a single BioNano map contig. Blue bars indicate five structural variations in one D genome and the corresponding positions in the other D genome regions.
