# Supplementary material for: Gene Duplication and Evolution Dynamics in the Homeologous Regions Harboring Multiple Prolamin and Resistance Gene Families in Hexaploid Wheat
Source: Front Plant Sci. 2018 May 23;9:673. doi: 10.3389/fpls.2018.00673 (PMC5974169; doi:10.3389/fpls.2018.00673)
Supplement: Supplementary file 6 [file Image_2.pdf]

Figure S2. Nucleotide and deduced amino acid sequences of prolamin genes

Note: LMW-B5, LMW-D8, and ω-A4 are retrieved by BLAST search against the CS PacBio contigs. Through BLAST search again the wheat genome sequence data (https://urgi.versailles.inra.fr/jbrowseiwgsc/gmod_jbrowse/), they are mapped to different genomic regions, likely resulted from translocation events.

δ-A1* 900 bp

ATGAAGATCTTCTTGGTCTTTGCCCTCCTCGTTGTATCAACGATCATCACCACCGCGACCGCGCAGCTCG
ACCCTAGCATCCATGTACAAGAAAGGCCACAACAATCGTTTCCGCAGCAGCAACCACTTACCCAACAACA
ACCATTCCCGCTGCAAGAGCCACAACAACCACTATTCCAGCAACAACAACCGTATCCACAACAGTCACTT
CGCCAACAACAACTTCCCCAGCAACATTTATTTCCGCAGCAACCGCCGCAACAACAATTTCCATAGTAGA
TGCCACTTCCGCATCAACAACAAATATTCCCGCAACAACAACAACCCCCACAACAACAACCATTTTACCA
ATATCAACAACCATTAACACAACAACCATACACGCGAGAGCAACCATTGCCACAACAACAACCTTCTGTG
GAGGAAAACCAACAATTGAACTTGTGCAAGGAGTTCCTGCTGCAGCAGTGCAACCCGGAGGAGAAAGTGT
CATTACTGCAGTCAATGATCCCGTTCCTCCGACCAAAGACCTCGCAACAGAACAACTGCCAGTTGAAGCG
GCAACAATGTTGTCGACAACTTGCACATATCAGCGAGCCGTCCCGATGTCCGGCCATCCACAACATTGTG
CACGCCATCATCATGCAACAACAACAACAACATGTGGATAGAGGTTTCGTCCAGCCTCAACCACAACAGT
TGGGCCAGGGAATGCCCATGCAGCCTCAACATCAATTGGGCCAGGGCTTAAGCCTACCTCAACAACTAGC
CCAGTTCAAGTTGGTTAGGTTACTTGTGATTCAGACCTTGCCTATGTTATGCAATGTGCATGTCCCATCT
GATTGCTACACCATTAGTGCACCATTTGGTGGCATCACTGCCTACAACGGTGGACAATGA

δ-A2* 892 bp

ATGAAGATCTTCTTGGTCTTTGCCCTCCTGGTTGCCACAACGATCAGCATCACCGCCACTGCGCAGCTCG
ACCCTAGCGTCCATGACTTAGAAAGGCCACACCAATCATTTCCACAACAGCAGCCACTTCCCCCGCTACA
ACCATTCCCGCCGCAAGAGCCATAACAACCATTTCCGTTGCAGCAACCACAACACCTGCAACAACAACCA
CTTCCCCAACAACATCTATTTCCGCAGCAACCGCCACAACAACAATTTCCACAGCAACAACAAACATTCC
CGCAACCTCATCAACAACCACAATTCCCGAAACAACAACCATTTTACCAATATCAACAACCATTTACACA
ACAACCATAACCGAAAGAGCAACCATTGGCACAACAACAACCTTGTGTAGAGGAAAGACAACGACTGAAC
TTGTGCAAGGAATTCCTCTTGCAGCAGTGCAACCCGGAGGAGAAGGTGTCGTCACTCCATTCGATGATCC
CGTTCCTCTGACCAAAGACCTCGCAACAGAATAGCTGCCAGTTGAAGCGACAAGAATGCTGTCGCCAACT
TGCACATATCCATGAGCAGTCTTGATGCCTGGCCATCCAAAGCCCTGTGCATGCCATCATCGTGCAACAA
CAACAACGACGACAACAACAACAGGTTGATAGAGGTTTTGGCCAGCCTCAACCCACCACAATTGGGCCAG
GGAATGCCCATGCAACCTCAATAGCCACTGGGCCAGGGCTTTATCCTACCTCAGCAACTAGCCCAGTTTA
AGTTGGTTAGGTCACTTGCGATTCAGATCCTGCCTATGTTATGCAACGTGCATGTCCCGTCTGATTGCTA
CACCATTACTGCATATTTTGGTAGCATGACTGCCTGCAGCTGTGGACAATGA

γ-A1 1020 bp

ATGAAGACCTTACTCATCCTGACAATCATTGCGGTGGCACTAACTACCACCACCGCCAATATACAGGTCG
ACCCTAGTGGCCAAGTACAATGGCCACAACAACAACAACCATTCCCCCAGCCCCAACAACCATTCTCCCA
ACAACCACAACAAATTTTTCCCCAACCCCAACAAACATTCCCCCATCAACCACAACAAGCATTTCCCCAA
CCCCAACAAACATTCCCCCATCAACCACAACAACAATTTCCCCAGCCCCAGCAACCACAACAACCATTTC
CCCAGCAACCACAACAACAATTTCCCCAGCCCCAACAACCACAACAACCATTTCCCCAGCAACCACAACA
ACAATTTCCCCAGCCCCAACAACCACAACAACCATTTCCCCAGCCCCAACAACCCCAACTACCATTTCCG
CAACAACCACAACAACCATTCCCCCAGCCTCAACAACCCCAACAACCATTTCCCCAGTTACAGCAACCAC
AACAACCTTTACCCCAGCCCCAACAACCGCAACAACCATTCCCCCAGCAACAACAACCATTGATTCAGCC
ATACCTACAACAACAGATGAACCCCTGCAAGAATTACCTCTTGCAGCAATGCAACCCTGTGTCATTGGTG
TCATCCCTCGTGTCAATGATCTTGCCACGAAGTGATTGCAAGGTGATGCGGCAACAATGTTGCCAACAAC
TAGCACAGATTCCTCAGCAGCTCCAGTGCGCAGCCATCCATGGCATCGTGCATTCCATCATCATGCAGCA
AGAACAACAACAACAACAACAACAACAACAACAACAACAACAACAACAAGGCATACAGATCATGCGGCCA
CTATTTCAGCTCGTCCAGGGTCAGGGCATCATCCAACCTCAACAACCAGCTCAATTGGAGGTGATCAGGT

CATTGGTATTGGGAACTCTTCCAACCATGTGCAACGTGTTTGTTCCACCTGAGTGCTCCACCACCAAGGC
ACCATTTGCCAGCATAGTCGCCGACATTGGTGGCCAATGA

γ-A1 340 aa

MKTLLILTIIAVALTTTTANIQVDPSGQVQWPQQQQPFPQPQQPFSQQPQQIFPQPQQTFPHQPQQAFPQ
PQQTFPHQPQQQFPQPQQPQQPFPQQPQQQFPQPQQPQQPFPQQPQQQFPQPQQPQQPFPQPQQPQLPFP
QQPQQPFPQPQQPQQPFPQLQQPQQPLPQPQQPQQPFPQQQQPLIQPYLQQQMNPCKNYLLQQCNPVSLV
SSLVSMILPRSDCKVMRQQCCQQLAQIPQQLQCAAIHGIVHSIIMQQEQQQQQQQQQQQQQQQGIQIMRP
LFQLVQGQGIIQPQQPAQLEVIRSLVLGTLPTMCNVFVPPECSTTKAPFASIVADIGGQ.

γ-A2* 873 bp

ATGAAGACCTTACTCATCCTCACAATCTTTGCGGCGGCTCTAACCATCGCCACCGCCAATATACAGGTCG
ACCCTAGTGGCCAAGTACAATGGCCACAACAACAACCATTCTCCCAGCCCCAACCATTCTCCCAGCAACC
ACAACAAGCGTTTCCCCAACCCCAACAAACATCCCCCCTTCAACCACAATAAGTATTTCCCCAACCACAA
CAACCACAACAACAATTTCCCCAGCCCCAGCAACCACAACAACCATTTCCCTAGCCCCAACAACCCCAAC
TACCATTTCCCCAACAACCCCAACAACCATTCCCCCAGCCTCATCAACCCCAACAACCATTTCCCCAGTC
ACAACAAACCACAACAACCTTTTCCCCAGCCCCAACAACAATTCCCGCAGCCCCAACAAAAGCAACAATC
ATTCCCTCAGCAACAACAACCGTTGATTCAGCCATATCTACAACAACAGATGAACCCCTGCAAGAATTAC
CTCTTGCAGCAATGCAACCCCGTGTCATTAGTGTCGTCCCTCGTGTCAATGATCTTGCCACGAAGTGACT
GCCAGGTGATGCAGCAACAATGTTGCCAACAACTAGCACAGATTCCTCGGCAGCTCCAGTGCACAGCCAT
CCATAGCGTGGTGCATGCCATCATCATGCAGCAAGAACAACAAGGCATACAGATCCTGCGACCACTGTTT
CAGCTTGTCCAGGACAGGGCATCATCCAACCTCAACAACCAGCTCAATATGAGGTGATCAGGTCATTGGT
ATTGAGAACCCTTCCAAACATGTGCAACATGTATGTCCGACCTAACTGCTCCACCATCAACGCACCATTT
GCCAGCATAGTTGCCGGCATTGGTGGCCAATGA

γ-A3 858 bp

ATGAAGACCTTATTCATCCTAACAATCCTTGCGATGGCAACAACTATCGCCACCGCGAATATGCAGGTCG
ACCCCAGCGGCCAAGTACAATGGCCACAACAACAACCATTCCGCCAGCCCCAACAACCATTCTACCAGCA
ACCACAACACACATTTCCCCAACCCCAACAAACATTCCCCCATCAACCACAACAACAATTTCCCCAGCCC
CAGCAACCACAACAACAATTTCCGCAGCCCCAGCAACCACAACAACCATTTCCCCAGCCCCAACAAGCCC
AACTACCATTTCCCCAACAACCACAACAACCATTCCCCCAGCCTCAACAACCCCAACAACCATTTCCCCA
GTCACAGCAACCACAACAACCTTTTCCCCAGCCCCAACAACCGCAACAATCATTCCCCCAGCAACAACAA
CCGTTGATTCAGCCATATCTACAACAACAGATGAACCCTTGCAAGAATTACCTCTTACAACAATGCAACC
CTGTGTCATTGGTGTCATCCCTCGTGTCAATGATATTGCCACGAAGTGATTGCCAGGTGATGCAGCAACA
ATGTTGCCAACAACTAGCACAGATTCCTCGCCAGCTCCAGTGTGCAGCCATCCATAGCGTCGTGCATTCC
ATCGTCATGCAGCAAGAACAACAACAAGGCATACAGATCCTCCGGCCACTGTTTCAGCTCGTCCAAGGTC
AGGGCATCATCCAACCTCAACAACCAGCTCAATATGAGGTGATCAGGTCATTGGTATTGAGAACCCTTCC
AAACATGTGCAACGTGTATGTCCGACCTGACTGCTCCACCATCAACGCACCATTTGCCAGCATAGTCGCC
GGCATCAGTGGACAATGA

>γ-A3 286 aa

MKTLFILTILAMATTIATANMQVDPSGQVQWPQQQPFRQPQQPFYQQPQHTFPQPQQTFPHQPQQQFPQP
QQPQQQFPQPQQPQQPFPQPQQAQLPFPQQPQQPFPQPQQPQQPFPQSQQPQQPFPQPQQPQQSFPQQQQ
PLIQPYLQQQMNPCKNYLLQQCNPVSLVSSLVSMILPRSDCQVMQQQCCQQLAQIPRQLQCAAIHSVVHS
IVMQQEQQQGIQILRPLFQLVQGQGIIQPQQPAQYEVIRSLVLRTLPNMCNVYVRPDCSTINAPFASIVA
GISGQ.

γ-A4 858 bp

ATGAAGACCTTATTCATCCTAACAATCCTTGCGATGGCAACAACTATCGCCACCGCGAATATGCAGGTCG
ACCCCAGCGGCCAAGTACAATGGCCACAACAACAACCATTCCGCCAGCCCCAACAACCATTCTACCAGCA
ACCACAACACACATTTCCCCAACCCCAACAAACATTCCCCCATCAACCACAACAACAATTTCCCCAGCCC
CAGCAACCACAACAACAATTTCCGCAGCCCCAGCAACCACAACAACCATTTCCCCAGCCCCAACAAGCCC
AACTACCATTTCCCCAACAACCACAACAACCATTCCCCCAGCCTCAACAACCCCAACAACCATTTCCCCA
GTCACAGCAACCACAACAACCTTTTCCCCAGCCCCAACAACCGCAACAATCATTCCCCCAGCAACAACAA
CCGTTGATTCAGCCATATCTACAACAACAGATGAACCCTTGCAAGAATTACCTCTTACAGCAATGCAACC
CTGTGTCATTGGTGTCATCCCTCGTGTCAATGATCTTGCCACGAAGTGATTGCCAGGTGATGCAGCAACA
ATGTTGCCAACAACTAGCACAGATTCCTCGCCAGCTCCAGTGTGCAGCCATCCATAGCGTCGTGCATTCC
ATCGTCATGCAGCAAGAACAACAACAAGGCATACAGATCCTCCGGCCACTGTTTCAGCTCGTCCAAGGTC
AGGGCATCATCCAACCTCAACAACCAGCTCAATATGAGGTGATCAGGTCATTGGTATTGAGAACCCTTCC
AAACATGTGCAACGTGTATGTCCGACCTGACTGCTCCACCATCAACGCACCATTTGCCAGCATAGTCGCC
GGCATCAGTGGACAATGA

>γ-A4 286 aa

MKTLFILTILAMATTIATANMQVDPSGQVQWPQQQPFRQPQQPFYQQPQHTFPQPQQTFPHQPQQQFPQP
QQPQQQFPQPQQPQQPFPQPQQAQLPFPQQPQQPFPQPQQPQQPFPQSQQPQQPFPQPQQPQQSFPQQQQ
PLIQPYLQQQMNPCKNYLLQQCNPVSLVSSLVSMILPRSDCQVMQQQCCQQLAQIPRQLQCAAIHSVVHS
IVMQQEQQQGIQILRPLFQLVQGQGIIQPQQPAQYEVIRSLVLRTLPNMCNVYVRPDCSTINAPFASIVA
GISGQ.

ω-A1* 987 bp

ATGAAGACCTTCCTCATCTTTGTCCTCCTTGCCATGGCGATGAACATCGCCACTGCCGCTAGGCAGCTAA
ACCCTAGCAACAAAGAATTACAATCACCTCAACAATCATTTTCCTATCAACAACAACCATTTCTACAACA
GCCATATCCACAACAACCATATCCATCACAGCAACCATATCCATCGCAACAACCATTTCCCACACCCCAA
CAACAATTTTCCCAGCAATCACAACAACCATTTCCCCAGACCCAACAATCATTCCCTCTGCAACCACAAC
AGCCATTCCCCCAGCAACCCCAACAACCATTTCCCCGGCCCCAACTACCATTCCCCCAGCAACCAGAACA
AATAATTCCCCAACAACCATTCCCCCTGCAACCGCAACAACCATTCCCCCAGCAACCCCAACAACCATTT
CCCCAGCCCCAACAACCAATCTCCGTGCAACCACAACAACCATTCCCCCAGCAATCCCAACAATCACAAC
AACCTTTTCCCTAGCCCCAACAATTATTTCTTGAACTCCAACAACCAATTCACCAGCAACCGCAACAACC
ATTCCCCCAACAACCGCAACAACCATTTCCCCAGCAACCGCAACAACCATTCCCCCAACAATCACAACAA
CCATCCCCCCAACAACCACAACAACCTTTCCCCCTACAACCACAACAACCATTCCCCCAACAACCACAAT
AATCATTCCTCCTAGGACCGCAACAACCATTTCCCCAGCAACCCCAACAATCACAACAATCATTTCCCCA
GCCCCAACCCCAGCAACCCCAACAACCATCCATCCTGCAACCACAACAACCATTACCCCAACAACCACAA
CAACCATTTCTACAGCCCCAACAACAATTATCCCAGCAACCAGAACAAACAATTTCCCAGCAACCCCAGC
AACCACACCAACCTCAACAACCATATCCACAACAACAACCATATGAGACTAGTCTTACAAGCATCGGTGG
CCAATGA

ω-A2* 1080 bp

ATGAAGACCTTCCTCATCTTTGTCCTCCTTGCCATGGCGATGAACACCGCCATTGCCGCTAGGCAACTAA
ACCCTAACAACAAAGAGTTACAATCACCTCAACAATCATTTTCCCATCAACAAAAACTATTTCCACAACA
GCCATATCCACAACAACCATATCCATCACAGCAACCATATCCATCACAACAACCATTTTCCACACCCCAA
CAACAATTTCCCCAGCAATCACAACAATTATTTCCCCAACCCCAACAACCGACCCCCTTACAACCACAAC
AACCATTCCCCCAGCAACCCCAACAACCACAACAACCTTTTCCACAGCCCCAACAACCATTTTCCTGGCA
ACCACAACAACCATTTCCCCAGACCCAACAATCGTTCCCTCTGCAACCACAACAGCCATTCCCCTAGCAA
GCCCAACAACCATTTCCCCAACCCCAACTACCATTCCCCCAGCAACCAGAACAAATAATTCCCCAGCAAC
CAGAACAAATAATTACCCAACAACCATTCCCCCTGCAACCCCAACAAGCATTCCCCCAGCAACCCCAACA
ACCATTTCCCCAGCCCCAACAACCAATCCCCGTGCAACCACAGCAACCATTCCCCCAGCAATCCCAACAA
TCACAACAACCTTTTCCCCGACCCCAACAATTATTTCCTGAACTCCAACAACCAATTCCCCAGCAAGCGC
AACAACCATTCCCCCAACAATCGCAACAACCATTCCCCCAGCAACCACAACAACCATTCCCCCTGCAACC
ACAACAATCATTCCCCCAACAACCACAACAACCATTCCCTCAACAACCACAACAACCTTTCCCTCTACAA

CCACAACAACCATTCCCCCTACGACCGCAACAACCATTTTCCCAGCAACCCTCCATCCTGCAACCACAAC
AACCACAACAACCATTTCTGCAGCCCCAACAACAATTATCCCAGCAACTAGAACAAACAATTTCCCAGCA
ACCCCAACAACCATTCCCCCAGCAACCACACCAACCTCAACAACCATATCCACAACAACAACCATCTGGG
AGTAGTCTTACAAGCATCGGTGGCCAATGA

ω-A3* 915 bp

ATGAAGACCTTCCTCATCTTTGTCCTCCTTGCCATGGCGATGAACACCGCCATTGCCGCTAGGCAGCTAA
ACCCTAGCAACAAAGAGTTACAATCACCTCAACAATCATTTTCCCATCAACAACAACCATTTCCACAACA
GCCATATCCACAACAACCATATCCATCATAGCAACCATATCCATCACAACAACCATTTTCCACACCCCAA
CAACAATTTCTCCAGCAATCACAACAATTATATCCCCAACCCCAACAACCGACCCCCTTACAACCACAAC
AACCATTCCCCCAGCAACCCCAACAACCACAACAACCTTTTCCACAGCCCCAACAACCATTTTCCTGGCA
ACCACAACAACCATTTCCCCAGACCCAACAATCGTTCCCTCTGCAACCACAACAGCCATTCCCCCAGCAA
CCCCAACAACCATTTCCCCAACCCCAACTACCATTCCCCCAGCAACCAGAACAAATAATTCCCCAGCAAC
CAGAACAAATAATTCCCCACCAACCATTCCCCCTGCAACCCCAACAAGCATTCCCCCAGCAACCCCAACA
ACCATTTCCCCAGCCCCAACAACCAATCCCCGTGCAACCACAACAACCATTCCCCCAGCAATCCCAACAA
TCACAACAACCTTTTCCCCGACCCCAACAATTATTTCCTGAACTCCAACAACCAATTCCCCAGCAAGCGC
AACAACCATTCCCCCAACAATCGCAACAACAATTCCCCCAGCAACCACACCAATCATTCCCCCTGCAACC
GCAACAATCATTCCCCCAACAACCACAACAACCATTCCCTCAACAACCACAACAACCTTTCCCTCTATAA
CCACAACAACCATTCCCCCTTCAACCGCAACAACCATTTTCCCAGCAACCCCAACAATCACAACAATCAT
TTCCCCAGCCCCAACCCCAGCAACCATCCATCCTGCAACCACAACAACCATTTTTGCAGCCCCAACAACA
ATTATCCCAGCAACTAGAACAAACAATTTCCCAGCAACCCCAACAACCATTCCCCCAGCAACCACACCAA
CCTCAACAACCATATCCACAACAACAACCATCTGGGAGTAGTCTTACAAGCATCGGTGGCCAATGA

ω-A4* 1118 bp

ATGAAGACCTTCCTCATCTTCGTCCTCCTTGCCATGGCGATGAGCATCGTCACTGCTGCTAGGCAGCTAA
ACCCTAGCAAGCAAGAGTTGCAATCACCACAACAATTATATCCGCAGCAACCATATCCACAGCAACCATA
TCCACCACAACAACCATTTCCCACACCCCAACAATATTTCCCCCATCAATCACAACAACCATTTTCCCCA
CCACAACAATCATTTCCCCAACCCCAACAAGCAACCCCCCTACAACCACAACAACCATTCCCCCAGCAAC
CCCAACAACCACAACAAGCTTTTCCCCAACCCCAACAACAATTTGCCTTGCAACCACAACAACAATTTCC
CCAGCTCCAACAACCACAACAATCATTCCCACAACAACCCCAGAGACCACACCCATTCCCCCAACAACTA
GAACAAGTAATTTCACAGCAACCACAACAACCATTCCTCCTGCAACCGCAACAACCATTCCCCCAGCAAC
CAGAACAAATAATATCCCAGCAACCCCAACAACTATTTTCTCAGTCACAACAACCATTTCCCCAGCAACC
CCAACAACCATTTCCCCTGCAACCGCAACAACCATTTCCCCAACAACCAGCACAAATAATTGCTCAGCAA
CCTCAACAACCATCCCCTCTGCAACCACAACAACCGTTCCTCCGGCAACCACAACAATCGTTCCTCCAGC
AACCACAACAACCATTTCCCCAACCCCAACAAGTAGTACAAATAAATCCCCAGCAACCCCAACAACCATT
TTCCCAGTCACGACAACCATTTCCCCAGCAACCCCAACAACCATTCCCACTGCAACCGCAACAACCATTT
CCCCAGCAATCAGCACAAATAATTCCTCAGCAACCCCAACAACCATTCCCTCTACAACCACAACAATCAT
TCCTTCGGCAATCACAACAACCGTTCCTCCAACAACCACAACAACCATCTCCCCAACCCCAACAAGTAGT
ACAAATAATTCCCCAGCAACCCCAACAACCATTCCCTCTGCTGGCAAACCAACCTCAGCAACCTTATCCA
CAACAGCAACCATCAGGAGTAATGGTATAGGCATCGGGGGCCAAGGAAATGAAGAGCTATAGTACTAG

LMW-A1* 999 bp

ATGAAGACATTCCTCGTCTTTTCCCTCCTCGCTCTTGCGGCGGCAAGTGCCGTTGCGCAAATTTCACAGC
AACAACAACAACAACAACCATTTCCGCAGCAACAACAACCACCATTTTCGCAGCAACAAGAGCCACCATA
TTCACAGCAATAGCAACCACCATTTTCGCAACAACAACAATCACCATTTTCGCAGCAACAACAACAACAA
CCACCATTTTTGCAGCAACACCAACCACCGTTTTCACAACAGCCACCAATTTCACAGCAGCAACAACCAC
CATTTTCACAGCAACAACAACCACCATTTTCACAGCAACAACAACCACCATTTTCGCAGCAGCAACAACA
ACAACCACCATTTTCACAACAACAACAACCACCGTTTTCACAACAGCCACCAATTTCACAGCAACAACAA
CCACCATTTTCGCAGCAACAACAACCACCATTTTCACAGCAACAACAAATACCAGTTATTCATCCATCTG

TTTTGCAGCAACTAAACCCATGCAAGGTATTCCTCCAGCAGCAGTGCATCCCTGTGGCAATGCAGCGATG
TCTTGCTAGGTCACAAATGTTGCAGCAGAGGATTTGCCATGTGATGCAGCAACAATGTTGCCAACAGTTG
CGGCAAATCCCCGGGCAATCCCGCCATGAGTCAATCCGTGCTATCATCTACTCTATCATCCTGCAGCAAC
AACAACAACAACAACAACAAGTTCAGAGTATCATCCAAGCTCAGCAACAACAACCCCAACAGTTGGGCCA
ATGTGTCTCCCAACCCCAACAACAATCGCAGCAGCAACTCGGGCAACAACCTCAACAACAACAATTGGCA
CAGGGTACCTTTTTGCAGCCACACCAGATAGCTCAATTTGAGGTGATGACTTCCATTGCACTCCGTACCT
TGCCAACGATGTGCAATGTCAACGTGCCGTTGTACGGAACCACCACTAGTGCGCCATTCGGCGTTGGCAC
TGGAGTTGGTGCCTACTGA

LMW-A2 1131 bp

ATGAAGACCTTCCTCGTCTTTGCCCTCCTCGCTCTTGCGGCGGCAAGTGCCGTTGCGCAAATTTCACAGC
AACAACAACAACCACCATTTTCACAGCAACAACAACCACCATTTTCGCAGCAACAGCAATCACCATTTTC
GCAACAACAACAACAACCACCATTTTTGCAGCAACAACAACCACCGTTTTCACAACAGCCACCAATTTCA
CAGCAGCAACAACCACCATTTTCACAGCAACAACAACCACAATTTTCACAGCAACAGCAGCCACCATATT
CGCAGCAACAACAGCCACCATATTCGCAGCAACAACAACCACCATTTTCGCAGCAACAGCAACCACCATT
TTCGCAGCAACAACAACCACCATTTTTGCAGCAACAACAACAACCACCATTTACACAGCAACAACAACCA
TCGTTTTCACAACAGCCACCAATTTCACAGCAGCAGCAGCAGCAACAACAACAACAACAACCATTTACAC
AGCAACAACAACCACCATTTTCACAACAGCCACCAATTTCACAGCAGCAACAACCACCATTTTCGCAGCA
ACAACAACCACCATTTTCACAGCAACAACAAATACCAGTTATTCATCCATCTGTTTTGCAGCAGCTAAAC
CCATGCAAGGTATTCCTCCAGCAGCAGTGCATCCCTGTGGCAATGCAGCGATGTCTTGCTAGGTCACAAA
TGTTGCAGCAGAGCATTTGCCATGTGATGCAGCAACAATGTTGCCAGCAGTTGCGGCAAATCCCCGAGCA
ATCCCGCCATGAGTCAATCCGTGCTATCATCTACTCTATCATCCTGCAGCAGCAGCAGCAGCAACAACAA
CAACAACAACAGGGTCAGAGTATCATCCAATATCAGCAACAACAACCCCAACAGTTGGGCCAATGTGTCT
CCCAACCCCAACAGCAGTTGCAGCAGCAACTCGGGCAACAACCTCAACAACAACAATTGGCACATGGTAC
CTTTTTGCAGCCACACCAGATAGCTCAGCTTGAGGTGATGACTTCCATTGCACTCCGTACCCTGCCAACA
ATGTGCAGTGTCAATGTGCCGTTGTACGAAACCACCACTAGTGTGCCATTAGGCGTTGGCATCGGAGTTG
GTGTCTACTGA

LMW-A2 377 aa

MKTFLVFALLALAAASAVAQISQQQQPPFSQQQQPPFSQQQQSPFSQQQQQPPFLQQQQPPFSQQPPIS
QQQQPPFSQQQQPQFSQQQQPPYSQQQQPPYSQQQQPPFSQQQQPPFSQQQQPPFLQQQQPPFTQQQQP
SFSQQPPISQQQQQQQQQQQPFTQQQQPPFSQQPPISQQQQPPFSQQQQPPFSQQQQIPVIHPSVLQQLN
PCKVFLQQQCIPVAMQRCLARSQMLQQSICHVMQQQCCQQLRQIPEQSRHESIRAIIYSIILQQQQQQQ
QQQQGQSIIQYQQQQPQQLGQCVSQPQQQLQQQLGQQPQQQQLAHGTFLQPHQIAQLEVMTSIALRTLPT
MCSVNVPLYETTTSVPLGVGIGVGVY.

LMW-A3* 915 bp

ATGAAGACCTTCCTCGTCTTTGCCCTCCTCGCCGTTGTGGCAACAAGTGCCATTGCGCAGATGGATACTA
GCTACATCCCTGGTTTGGAGAGACCATGGCAGCAGCAACCATTACCACCACAACAGACATTTCCACAACA
ACCACCATTTTCACAATAACAACAACAACAACAACAACAACCATTTCCTCAACAACCATCATTTTCACAG
CAACAACCACCATTTTCACAGCAACAACCAATTCTACCACAGGGACCACCATTTTCACAGCAAACACAAC
CTGTTCTACCGCAACAATCACCATTTTCACAGCAACAACAACTAATTTTACCTCCACAACAACAACAACA
GCTTCCGCAACAACAAATCTCTATTGTTCAACCATCCGTTTTGCAGCAGCTAAACCCATGCAAGGTATTC
CTCCAGCAGCAGTGCAGCCCTGTGGCAATGCCACAACGTCTTGCTAGGTCGCAAATGTGGCAGCAGAGCC
GTTGCCATGTGATGCAGCAACAATGTTGCCAGCAGTTGTCGCAAATCCCCGAACAATCCCGCTATGATGC
AATCCGTGCCATCACCTACTCCATCATCCTACAAGAACAACAACAGGGTTTTGTCCAAGCTCAGCAGCAA
CAACCCCAACAGTCAGGTCAAGGTGTCTCCCAATCCCAACAGCAGTCGCAGCAGCAGCTCGGACAATGTT
CTTTCCAACAACCTCAACAGCAACTGGGTCAACAGCCTCAACAACAACAGGTACAACAGGGTACCTTTTT
GCAGCCACACCAGATAGCTCACCTTGAGGTGATGACTTCCATTGCGCTCCGTACCCTGCCAACGATGTGC
AGTGTCAATGTGCCATTGTACAGCTCCACCACTAGTGTGCCATTCGGAGTTGGCACCGGAGTTGGTGCCT
ACTGA

LMW-A4* 886 bp

ATGAAGACCTTCCTCGTCTTTGCCCTCCTCACCGTTGTGGCGACAAGTACCATTGCGCAGATGGAGACTA
GCTGCATCCCTGGTTTGGAGAGACCATGGCAGCAGCAACCATTACCACCACAACAGACATTATTTCCACA
ACAACAACCATTTCCACAACAACAACCACCATTTTCACAACAACAACCATCATTTTCGCAACAACAACCA
CCATTTTCGCAGCAACAACCAATTCTACCGCAGCCACCATTTTCACTGCAACAACAACCAGTTCTACCGC
AACAATCACCTTTTTCGCAGCAACAACTAGTTTTACCTCCACAACAACAATACCAACAGCTTCTGCAACA
ACAAATCCCTATTGTTCAGCCATCCGTTTTGCAGCAGCTAAACCCATGCAAGGTATTCCTCCAGCAGCAG
TGTGCCACAACGTCTTGCTAGGTCGCAAATGTGGCAGCAGAGCAGTTGCCATGTGATGCAGCAACAATGT
TGCCAGCAGTTGCCGCAAATCCCCGAACAATCTCGCTATGATGCAATCCGTGCCATCACCTACTCCATCA
TCTTACAAGAACAACAACAGGGTTTTGTCCAAGCTCAGCAGCAACAACCCCAACAGTTGGGTCAAGGTGT
CTCCCAATCCCAACAACAATCGCAGCAGCAGCTCGGACAATGTTCTTTCCAACAACCTCAACAGCAACTG
GGTCAACAGCCTCAACAACAACAGATACTACAGGGTACCTTTTTGCAGCCACACCAGATAGCTCACCTTG
AGGTGATGACTTCCATTGCACTCCGTACCCTGCCAACGATGTGCAGTGTCAATGTGCCGTTGTACAGCTC
CACCACTAGTGTGCCATTCAGTGTTGGCACCGGAGTTGGTGCCTAC

δ-B1 954 bp

ATGAAGATCCTCTTGGTCTTTGCCCTCCTCGTTGTATCAACGACCATCACCACCGCGATCGTGCAGCTCG
ACCCTAGTGTCCATGTCCAAGAAAGGCCACAACAATCATTTCTGCAGCAGCAACCACTTACCCAGCAACA
ACCATTGCCGCTGCAAGAACCACAACAACCACTATTCCCGCAAAAAGAGCCACAACAACCATTTTCGCTG
CAGCAACCACAACCCCAGGAACAACAACCATATCCACAACAGCCACTTCTCCAACAACAACTTCCCCAGC
AACATCTATTCCCGCAGCAGCCACCACAACAACAATTTCCGCAGCAGATGCCACTTCCATATGAACAACA
AACATTCCCGCAACAACAGCAACAGCAACCACAATTCCCGCAACAACAACCATTTCCCCAATATCAACAA
CCATTAACACAACAACCATACCCGCAAGAGCAACCATTGCCACAACAACAACCTTCTGTGGAGGAAAAAC
AACAATTGAACTTGTGCAAGGAGTTCCTCCTGCAACAGTGCAACCCGGAGGAGAAACTGTCATTAATCCA
GTCAGTGATCCCGTTCCTCCGACCAAAGACCTCGCAACAGAATAGCTGCCAGTTGAAGCGTCTACAATGT
TGTCGACAACTTGCACGTATCAATGAACCGTCCCGATGCCCGGCCATCCACAACATTGTGCATGCCATCG
TCATGCAACAACAACATGTGGATAGAGGTTTCGGCCAGCCTCAACCACAACAGTTGGGCCAGGGAATGCC
CATGCAGCCTCAATATCAATTGGGCCAGGGCTTTATCCTACCTCAACAACTAGCCCAGTTCAAGTTGGTT
AGGTTACTTGTGATTCAGACCCTGCCTATGTTATGCAACGTGCATGTCCCATCTGACTGCTACACCACTA
GTGCACCATTTGGTAGCATGACTGCCATGAACGGTGGACAGTGA

δ-B1 318 aa

MKILLVFALLVVSTTITTAIVQLDPSVHVQERPQQSFLQQQPLTQQQPLPLQEPQQPLFPQKEPQQPFSL
QQPQPQEQQPYPQQPLLQQQLPQQHLFPQQPPQQQFPQQMPLPYEQQTFPQQQQQPQFPQQQPFPQYQQ
PLTQQPYPQEQPLPQQQPSVEEKQQLNLCKEFLLQQCNPEEKLSLIQSVIPFLRPKTSQQNSCQLKRLQC
CRQLARINEPSRCPAIHNIVHAIVMQQQHVDRGFGQPQPQQLGQGMPMQPQYQLGQGFILPQQLAQFKLV
RLLVIQTLPMLCNVHVPSDCYTTSAPFGSMTAMNGGQ.

γ-B1 909 bp

ATGAAGACCTTACTCATCCTAACAATCCTTGCGATGGCAACAACCATCGCCACCGCCAATATGCAAGTCG
ACCCCAGCGGCCAAGTACAATGGCCACAACAACAACCATTCCCCCAGCCCCAACAACCATTCTGCCAGCA
ACCACAACGAACTATTCCCCAACCCCATCAAACATTCCACCATCAACCACAACAAACATTTCCCCAACCC
CAACAAACATACCCCCATCAACCACAACAACAATTTCCCCAGACCCAACAACCACAACAACCATTTCCCC
AGCCCCAACAAACATTCCCCCAACAACCCCAACTACCATTTCCCCAACAACGCCAACAACCATTCCCCCA
GCCTCAGCAACCCCAACAACCATTTCCCCAGTCACAACAACCACAACAACCTTTTCCCCAGCCCCAACAA
CAATTTCCGCAGCCCCAACAACCACAACAATCATTCCCCCAACAACAACAACCGGCGATTCAGTCATTTC
TACAACAACAGATGAACCCCTGCAAGAATTTCCTCTTGCAGCAATGCAACCATGTGTCATTGGTGTCATC
TCTCGTGTCAATAATTTTGCCACGAAGTGATTGCCAGGTGATGCAGCAACAATGTTGCCAACAACTAGCA

CAAATTCCTCAACAGCTCCAGTGCGCAGCCATCCACAGCGTCGCGCATTCCATCATCATGCAACAAGAAC
AACAACAAGGCGTGCCGATCCTGCGGCCACTATTTCAGCTCGCCCAGGGTCTGGGTATCATCCAACCTCA
ACAACCAGCTCAATTGGAGGGGATCAGGTCATTGGTATTGAAAACTCTTCCAACCATGTGCAACGTGTAT
GTGCCACCTGACTGCTCCACCATCAACGTACCATATGCCAACATAGACGATGGCATTGGTGGCCAATGA

γ-B1 303 aa

MKTLLILTILAMATTIATANMQVDPSGQVQWPQQQPFPQPQQPFCQQPQRTIPQPHQTFHHQPQQTFPQP
QQTYPHQPQQQFPQTQQPQQPFPQPQQTFPQQPQLPFPQQRQQPFPQPQQPQQPFPQSQQPQQPFPQPQQ
QFPQPQQPQQSFPQQQQPAIQSFLQQQMNPCKNFLLQQCNHVSLVSSLVSIILPRSDCQVMQQQCCQQLA
QIPQQLQCAAIHSVAHSIIMQQEQQQGVPILRPLFQLAQGLGIIQPQQPAQLEGIRSLVLKTLPTMCNVY
VPPDCSTINVPYANIDDGIGGQ.

γ-B2 894 bp

ATGAAGACCTTACTCATCCTAACAATCCTTGCGATGGCAACAACAATCGCCACTGCCAATATGCAGGTCG
ACCCTAGCAGCCGAGTACAATGGCCACAAGAACGACCTTTCCCCCAGTCCCAACAACCATTCTCCCAGCA
ACCACAACAAATATTTCCCCAACCCCAACAAACATTCCCCCATCAACCACAACAAGCATTTCCCCAACCT
CAACAAACATTCCCCCATCGACCACAACAACAATTTCCCCAGCCCCAGCAACCACAACAACCATTTCCCC
AGCCCCAACAACCCCAACTACCATTTCCCCGACAACCACAACAACCATTCCCCCAGCCTCAACAACCCCA
ACAACCATTTCCCCAGTCACAGCAACCACAACAACCTTTTCCCCAACCCCAACAACAATTTCCGCAGCCC
CAACAACCACAACAATCATTCCCCCAACAACAACAACTGATGATTCAGTCATTTCTACAACAACAGATGA
ACCCCTGCAAGAATTTTCTCTTGCAGCAATGCAACCCTATGTCATTGGTGTCATCTCTCGTGTCAATAAT
CTTGCCACGAAGTGATTGCCAGGTGATGCAGCAACAATGTTGCCAACAACTAGCACAAATTCCTCAACAG
CTCCAGTGCGCAGCCATCCACAGCGTCGCGCATTCCATCATCATGCAGCAAGAACAACAACAAGGCGTGC
AGATCCTGCGACCACTATTTCAGCTCGCCCAGGGTCTGGGTATCATCCAACCTCAACAACCAGCTCAATT
GGAGGGGATCAGGTCATTGGTATTGAAAACTCTTCCAACCATGTGCAACGTGTATGTCCCACCTGACTGC
TCCACCATCAACGTACCATATGCCAGCATAGACGCCGGCATTGGTGGCCAATGA

γ-B2 298 aa

MKTLLILTILAMATTIATANMQVDPSSRVQWPQERPFPQSQQPFSQQPQQIFPQPQQTFPHQPQQAFPQP
QQTFPHRPQQQFPQPQQPQQPFPQPQQPQLPFPRQPQQPFPQPQQPQQPFPQSQQPQQPFPQPQQQFPQP
QQPQQSFPQQQQLMIQSFLQQQMNPCKNFLLQQCNPMSLVSSLVSIILPRSDCQVMQQQCCQQLAQIPQQ
LQCAAIHSVAHSIIMQQEQQQGVQILRPLFQLAQGLGIIQPQQPAQLEGIRSLVLKTLPTMCNVYVPPDC
STINVPYASIDAGIGGQ.

γ-B3* 1065 bp

ATGAAGACCTTAATCATCCTGACGATCCTTGCGATGGCAATAACCATCAGCACCGCCAATATGCAGGTCG
ACCCTAGCGGCCAAATACAATGGTCACAACAACAACTAGTCCCCCAGCCCCAAGAGCCATTATCCTAGCA
ACCACAACAAGCATTTCCCCAACCCCAACAAACATTCCCCCATCAACCACAACAACAAGTTCCCCAGCCT
CAGCAACCACAACAACCATTTCTCCAGCCCCAACAAGCATTCCCCCAACAACCACAACAACCATTCCCCC
AGACTCAACAACCACAACAACCATTTTCCCAGCAACCACAACAACCATTTCCCCAGACTCAACAGCCACA
ACAACCATTTCCCCAGCAACCACAACAACTATTCCCCCAGACTCAACAACCACAACAACCATTTCCCCAG
CAACCACAACAACCATTCCCCCAGACTCAACAACCACAACAACCATTTCCCCAGTTCCAGCAACCACAAC
AACCTTTTCCCCAGCCCCAACAACAATTCCCGCAGCCCCAACAACCGCAACGATCATTCCCCCAGCAACA
AAGACCGTCCTTTCAGCCATCTCTACAACAACGGTTGAACCCATAAAAGAATATCCTCTTGCAACAATGC
AAACCTGCATCATTGGTGTCATCCCTTTGGTCGATAATCTGGCCACAAAATGATTGCCAAGTGATGCAGC
AACAATGCTGCCAAGAACTAGCACAAATTCCTCAGCAGCTCCAGTGTGCAGCCATCCATAGCGTCGTGCA
TTCCATCATCATGCAGCAACAAGAACAAGAACAAGAACAACAACAACAACAACAACAACAACAACAGCAA
GGCATGCGTATCTTTTTTCCACTATCTCGACAACAACATGTGGGTCAAGGTACTCTCGTCCAAGGCCAGG

GCATCATCCAACCTCAACAACCAGCTCAATTGGAGGCGATCAGGTCATTGGTGTTGCAAACTCTTCCAAC
CATCTGCAATGTGTATGTCCCACCTGAGTGCTCCAACATCAGGGCACCATTTGCCAGCATAGTCGCGGGG
ATTGGTGGCCAATGA

γ-B4 1107 bp

ATGAAGACCTTACTCATCCTGACGATCCTTGCGATGGCAATAACCATCAGCACCGCCAATATGCAGGTCG
ACCCTAGTGGCCAAGTACAATGGCCACAACAACAACTAGTCCCCAACCCCAACAGCCATTATCCCAGCA
ACCGCAACAAGCATTTCCCCAACCCCAACAAACATTTCCCCATCAACCACAACAACAAGTTCCCCAGCCT
CAGCAACCACAACAACCATTTCTCCAGCCCCAACAAGCATTCCCCCAACAACCACAACAACCATTCCCTC
AGACTCAACAACCACAACAACCATTTCCCCAGCAACCACAACAACCATTCCCCCAGACTCAACAGCCACA
ACAACCATTTCCCCAGCAACCACAACAACCATTTCCCCAGCAATCACAACAACCATTCCCCCAGACTCAA
CAACCACAACAACCATTTCCCCAGCAACCACAACAACCATTCCCCCAGACTCAACAACCACAACAACCAT
TTCCCCAGTTCCAGCAACCACACCAACCTTTTCCCCAGCCCCAACAACAATTCCCGCAGCCCCAACAACC
GCAACAATCATTCCCCCAGCAACAACGACCGTTCATTCAGCCATCTCTACAACAACGTTTGAACCCATGC
AAGAATATCCTCTTGCAACAATGCAAACCTGCGTCATTGGTGTCATCCCTCTGGTCGATAATCTGGCCAC
AAAGCGATTGCCAAGTGATGCAGCAACAATGCTGCCAAGAACTAGCACAGATTCCTCAGCAGCTCCAGTG
CGCAGCCATCCATAGCGTCGTGCATTCCATCATCGTGCAGCAGCAGCAACAACAACAACAACAACAACAA
CAACAACAAGGCATGCATATCCTGCTGCCACTATCTCAACAACAACAGTTGGGTCAAGGTACTCTCGTCC
AAGGCCAGGGCATCATCCAACCTCAGCAACTAGCTCAATTGGAGGCGATCAGGTCATTGGTGTTGCAAAC
TCTTCCAACCATGTGCAACGTGTATGTCCCACCTGAGTGCTCCATCATCAGGGCACCATTTGCCAGCATC
AGGGCATCATCAGGGCACCATTTGCCAGCACTAGTCGCGGGGATTGGTGGCCAATGA

γ-B4 369 aa

MKTLLILTILAMAITISTANMQVDPSGQVQWPQQQLVPQPQQPLSQQPQQAFPQPQQTFPHQPQQQVPQP
QQPQQPFLQPQQAFPQQPQQPFPQTQQPQQPFPQQPQQPFPQTQQPQQPFPQQPQQPFPQQSQQPFPQTQ
QPQQPFPQQPQQPFPQTQQPQQPFPQFQQPHQPFPQPQQQFPQPQQPQQSFPQQQRPFIQPSLQQRLNPC
KNILLQQCKPASLVSSLWSIIWPQSDCQVMQQQCCQELAQIPQQLQCAAIHSVVHSIIVQQQQQQQQQQ
QQQGMHILLPLSQQQQLGQGTLVQGQGIIQPQQLAQLEAIRSLVLQTLPTMCNVYVPPECSIIRAPFASI
RASSGHHLPALVAGIGGQ.

γ-B5* 1035 bp

ATGAAGACCTTACTCATCCTAACAATCCTTGTGATGGCAGTAACCATCGGCACCGCCAATATGCAGGTCG
ACCCTAGCGGCCAAGTACAATGGCCACAACAACAACCAGTCCTGCTGCCTCAGCAACCATTCTCCCAGCA
ACCACAACAAACATTTCCCCAACCCCAACAAACATTCCCCCATCAACCACAACAACAATTTCCCCAGCCT
CAACAACCACAACAACAATTTCTCCAGCCACAACAACCATTCCCCCAACAACCACAACAACCATATCCCC
AGCAACCACAACAACCATTCCCCCAGACTCAACAACCCCAACAACTATTTCCCCAGTCCCAGCAACCACA
ACAACCATATCCCCAGCAACCACAACAACCATTCCCCCAGACTCAACAACCCCAACAACAATTTCCCCAA
TCCCAGCAACCACAATAACCATTTCCCCAACCCCAACAACCGCAACAATCATTCCCCCAGCAACAACCAT
CGTTCATTCAGCCATCTCTACAACAACAGCTGAACCCATGCAAGAATTTACTCTTGCAGCAATGCAGACC
TGTGTCATTGGTCTCATCCCTCTGGTCAATGATCTGGCCACAAAGCGCTTGCCAAGTGATGAGGCAACAA
TGCTGCCAACAACTAGCACAGATTCCTCAGCAGCTCCAGTGTGCAGCCATCCATAGCGTCGTGCATTCCA
TCAGCATGTAGCAAGAACAACAACAACAACAACAACAACAACAACAACAACAACAACAACAACAACAACA
ACAACAACAACAACAACAACAACAACAACAACAACAACAAGGCATGCGTATCCTGCTGCCACTATATCAG
CAACAACAGGTGGGTCAAGGTACTCTCGTCCAGGGCCAGGGCATCATCCAACCCCAACAACCAGCTCAAT
TGGAGGCGATCAGGTCATTGGTGTTGCAAACTCTTCCAACCATGTGCAACGTGTATGTCCCACCTGAGTG
CTCTATCATCAAGGCACCATTTGCCAGCATAGTCACCGGAATTGGTGGCCAATGA

γ-B6 876 bp

ATGAAGACCTTACTCATCCTGACAATCCTTGCGATGGCAATAACCATCGCCACCGCCAATATGCAGGCCG

ACCCTAGCGGCCAAGTACAATGGCCGCAACAACAACCATTCCTGCAGCCTCACCAACCATTCTCCCAGCA
ACCACAACAAATATTTCCCCAACCCCAACAAACATTCCCCCATCAACCACAACAACAATTTCCCCAGCCT
CAGCAACCACAACAACAATTTCTCCAGCCCCGACAACCATTCCCCCAACAACCACAACAACCATATCCCC
AGCAACCACAACAACCGTTCCCCCAGACTCAACAACCCCAACAACCATTTCCCCAGTCCAAGCAACCACA
ACAACCTTTTCCCCAGCCCCAACAACCGCAACAATCATTCCCCCAACAACAACCATCGTTGATTCAACAA
TCTCTACAACAACAGTTGAACCCATGCAAGAATTTCCTCTTGCAGCAATGCAAACCTGTGTCCTTGGTGT
CATCCCTCTGGTCAATCATCTTGCCACCAAGCGATTGCCAGGTGATGCGGCAACAATGTTGTCAACAACT
AGCACAAATTCCTCAGCAACTCCAGTGTGCAGCCATCCATAGCGTCGTGCATTCCATCATCATGCAGCAA
GAACAACAAGAACAACTACAGGGTGTGCAAATCCTGGTGCCACTGTCTCAACAGCAACAGGTGGGTCAAG
GTATTCTCGTCCAGGGTCAAGGCATCATCCAACCTCAACAACCAGCTCAATTGGAGGTGATCAGGTCATT
GGTGTTGCAAACTCTTCCAACCATGTGCAACGTGTATGTCCCACCTTACTGCTCCACCATCAGGGCACCA
TTTGCTAGCATAGTCGCCAGCATTGGTGGCCAATGA

γ-B6 292 aa

MKTLLILTILAMAITIATANMQADPSGQVQWPQQQPFLQPHQPFSQQPQQIFPQPQQTFPHQPQQQFPQP
QQPQQQFLQPRQPFPQQPQQPYPQQPQQPFPQTQQPQQPFPQSKQPQQPFPQPQQPQQSFPQQQPSLIQQ
SLQQQLNPCKNFLLQQCKPVSLVSSLWSIILPPSDCQVMRQQCCQQLAQIPQQLQCAAIHSVVHSIIMQQ
EQQEQLQGVQILVPLSQQQQVGQGILVQGQGIIQPQQPAQLEVIRSLVLQTLPTMCNVYVPPYCSTIRAP
FASIVASIGGQ.

ω-B1* 1309 bp

ATGAAGACCTTCATCATATTTGTCCTCCTTGCCATGACAATGAACAACGCCAGTGCCACTAGGCAACTAA
GCCCTATAGGCAAGGAATTGCATACACAACAACAACAATTCCCCCAACCACAACAATTCCCTCAACCACA
ACAAATCCCCCAACAACAACTCCCCCAACAACATCAAATCCCCCCAGCAATCACAACAATTCCCCCAACA
ACAAATCCCCCAACCACAATAACTCCCCCAGCAACAATTCCCTCAACAGCAGCAATTCCCGCAACAACAA
CAATTCCCCCAACAACAATTTCCCCAATAGCAATTCCCACAATAGAAATTCCCACAACAACAACAATTCC
CGCAACAACAAATCCCCCAGCAACCACAACAACTCCCCCAACAACAACAAATCCCCCAACAACCACAACA
ATTTCCTCAACAACAAAAATTCCTCCAGCAACAATCCCCCCAACAACAGCAATTTCTACAACAGCAATTC
CCCCAACAACAACAATTTCCCTAACAACAATTACCACAACAACAATTCCCTGAACCACAACAAATCCCCC
AACAACAACAAATTCCCCAGCAACCACAACAATTCCCGCAGCAACAATTTCCCCAACAACAACAATTTCC
CCAACAACACCAATTCCCGCAACAATAATTCCCCCAATAGCAATTCCTGCAACACATCCCACAAAAACCA
CATCAATTTCCCCAACAACAACAATTCCCCCAGCAACAATCCCCCTAATAACAACAATTTCCCCAACAAC
AGCAATTCCCGCAACAACAATTCCCCCAACAACCATTCCCGCAACAACAATTTCCCCAACAATAATTCCC
CCAACAGAAATCCCCCCAACAACAACAATTCCCCCAACAGCAATTCCCGCAACACATCCCACAACAACCA
CATCAATTTCCCGAACAACAACAATTCCCCGAGCAACAATCCCCCCAATAACAACAATTTCCCCAAAAAC
AGCAATTCCCGCAACAACAATTCCCCAAACAACCATTCCCGCAACAACAATTTCCCCAACAATAATTCCC
CCAACAGAAATCCCCCCAACAACAACAATTCCCCCAACAACAACAATTACCGCAACAACAATTCTCCCAA
CCACAAAAATACCTCGAACAACAACAATTACCCCAACAACAATTCCCCCAGCAACCACCACAACAATTTT
CCCAACAACAATTTCCAATACCATACCCACCCCAGCAATCACAAGAACCTTCCCCATACAAACAATATCC
ACAACAACAACCATCTGGGAGCGACGTTATAAGTATCAGTGGCAGATGA

ω-B2* 1225 bp

ATGCAATACTCCACAAGAACAATTCCCCCAACAACAACAAGTCCCCCAACCACAACAATTCTCCCAACAA
CAAATCCCCCAACAACATCAAATCCCCCAGCAACCAGAACAACTCCCCCAACAACAACAAATCCCCCAAC
AACAAATCCCCCAACAACATCAAATCCCCCAGCAACCACGACAATTCCCCCAACAACAATTCCCCCAACA
ACAACAATTCCCCCAACAACAGCAATCCCCCCAACAACAATTCCCACAACAACAATTCGCCCAACAACAA
TTGCCGCAACAGGAATTTCCACAACAACAAATCCCCCAGCAACCACAACAACTCCCCCAACAACAAAAAA
TCCCCCAGCCACCACAACAATTTCTCCAACAACAACAATTCCCCTAGCAACAATCCCCCCAACAACATCA
ATTACCCCAATAGCAATTGCCGCAACAACAACAAATCCCCCATCAACAACAAATCCCCCAGCAACCACAA
CAAATCACCCAACGACAACAAATCCCCTAGCAACCACAACAATTCCCCCAACAACAAATCCCGCAACAAC

AATTCCCCCAACATCAATTCCCACAACAGGAATTCCCATAACAACAACAATTCCCGCAACAACAAATCAC
CCAGCAACCACAACAACTCCCCCAACAACAACAAATCCCCCAGCAACCACAACAATTTCCCCAACAACAA
CAATTCCCCCAGCAACAATCACCCCAACAACAGCAATTTCCCCAACAACAATTCCCCCAACAACAACAAT
TACCGCAACAACAATTCCCCCAACCATAACAAATACCCCAACAAAAACAAATCCCCCAGCAACAATTCCC
CCAACAACAATTCCCCCCAAAACAACAATTCCCCCAACGGCAATTCCCGCAACAACAATTCCCCCAACAT
CAATTCCCCCAACAACAACAGTTCCCCAAACAACAATTACCGCAACAACAATTCCCCCAACAGCAATTCC
CCCAACAACAACAGTTCCCCCAACAAGAACAATTACCACAACAAGAATTCCCCCAGCCACAACAATCCCC
TGAACAACAACAATTCCCCCAACAACAATTCCCCCTGCAACCACCACAACAATCCCCCCAAAAACAATTT
CCAATACCATACCCACCCCAGCAATCACAAGAACCTTCCCCATACCAACAATATCCACAACAACAACCAT
CTGGGAGCGACGTTATAAGTATCAGTGGCCTATGA

ω-B3 1236 bp

ATGAAGACCTTCATCATATTTGTCCTCCTTGCCATGGCGATGAACATCGCCAGTGCCAGTAGGCTGCTAA
GCCCTAGAGGCAAGGAATTGCATACTCCACAAGAACAATTCCCCCAACAACAACAATTCCCCCAACCACA
ACAATTCCCCCAACAACAAATCCCCCAACAACATCAAATCCCCCAGCAACCACAACAATTCCCCCAACAA
CAACAATTCCTCCAACAACAACAAATCCCGCAACAACAAATCCCCCAACAACATCAAATCCCCCAGCAAC
CACAACAATTCCCCCAGCAACAGCAATTCCCCCAACAACACCAATCCCCCCAACAACAATTCCCACAACA
ACAATTCCCCCAACAGAAATTGCCGCAACAGGAATTCCCACAACAACAAATCTCCCAGCAACCACAACAA
CTCCCCCAGCAACAACAAATCCCCCAGCAACCACAACAATTTCTCCAACAACAACAATTCCCCCAGCAAC
CACAACAAATCCCCCAACAACAACAAATCCCCCAGCAACCACAACAATTCCCCCAACAACAATTCCCGCA
ACAACAATTTCCCCAACAGCAATTCCCGCAACAGGAATTCCCACAACAACAACAATTCCCGCAACAACAA
ATCACCCAGCAACCACAACAACTCCCCCAACAACAACAAATCCCCCAGCAACCACAACAATTTCCCCAAC
AACAACAATTCCCCCAGCAACAATCACCCCAACAACAGCAATTTCCCCAACAACAATTCCCCCAACAACA
ACAATTACCGCAAAAACAATTCCCCCAACCACAACAAATACCCCAACAACAACAAATCCCCCAGCAACCA
CAACAATTCCCCCAGCAACAATTCCCCCAACAACAGCAATTTCCCCAACAACAAGAATTCCCCCAACAGC
AATTCCCGCAACAACAATTCCACCAACAACAATTACCGCAACAACAATTTCCCCAACAACAATTCCCCCA
ACAACAATTCCCCCAACAACAACAGTTCCCCCAACAACAACAATTAACACAACAACAATTCCCCCGGCCA
CAACAATACCCTGAACAACAACAATTCCCCCAACAACAATTCCCCCAGCAACCACCACAACAATTCCCCC
AACAACAATTTCCAATACCATACCCACCCCAGCAATCACAAGAACCTTCCCCATATCAACAATATCCACA
ACAACAACCATCTGGGAGCGACGTTATAAGTATCAGTGGCCTATGA

ω-B3 412 aa

MKTFIIFVLLAMAMNIASASRLLSPRGKELHTPQEQFPQQQQFPQPQQFPQQQIPQQHQIPQQPQQFPQQ
QQFLQQQQIPQQQIPQQHQIPQQPQQFPQQQQFPQQHQSPQQQFPQQQFPQQKLPQQEFPQQQISQQPQQ
LPQQQQIPQQPQQFLQQQQFPQQPQQIPQQQQIPQQPQQFPQQQFPQQQFPQQQFPQQEFPQQQQFPQQQ
ITQQPQQLPQQQQIPQQPQQFPQQQQFPQQQSPQQQQFPQQQFPQQQQLPQKQFPQPQQIPQQQQIPQQP
QQFPQQQFPQQQQFPQQQEFPQQQFPQQQFHQQQLPQQQFPQQQFPQQQFPQQQQFPQQQQLTQQQFPRP
QQYPEQQQFPQQQFPQQPPQQFPQQQFPIPYPPQQSQEPSPYQQYPQQQPSGSDVISISGL.

ω-B4* 1299 bp

ATGAAGACCTTCATCATATTTGTCCTCCTTGCCATGGCGATGAACATCGCCAGTGCCAGTAGGCTGCTAA
GCACTAGAGGCAAGGAATTGCATACTCCACAAGAACAATTCCTCCAACAACAACAATTCCCCCAACAACA
AATCCCCCAACAACATCAAATCCCCCAGCAACCACAACAATTCCCCCAACAACAACAATTCCTCCAACAA
CAACAAATCCCGCAACAACAAATCCCCCAACAACATCAAATCCCCCAGCAACCACAACAATTCCCCCAGC
AACAGCAATTCCCCCAACAACACCAATCCCCGCAACAACAATTCCCACAACAACAATTCCCCCAACAGAA
ATTGCCGCAACAGGAATTCCCACAACAACAAATCTCCCAGCAACCACAACAACTCCCCCAGCAACAACAA
ATCCCCCAGCAACCACAACAATTTCTCCAACAACAACAATTCCCCCAGCAACAACCCCCCCAACAACATC
AATTTCCCCAACAGCAATTGCCCCAACAACAACAAATCCCCCAACAACAACAAATCCCCCAGCAACCACA
ACAAATCCCCCAACAACAACAAATCCCCCAGCAACCACAACAATTCCCCCAACAACAATTCCCGCAACAA
CAATTTCCCCAATAGCAATTCCCGCAACAGGAATTCCCACAACAACAACAATTCCCGCAACAACAAATCG
CCCGCACACAACAACTCCCCCAACAACAACAAATCCCCCAGCAACCACAACAATTTCCCCAACAACAACA

ATTCCCCCAGCAACAATCACCCCAACAACAGCAATTTCCCCAACAACAATTCCCCCAACAACAACAATTA
CCGCAAAAACAATTCCCCCAACCACAACAAATACCCCAACAACAACAAATCCCCCAGCAACCACAACAAT
TCCCCCAGCAACAATTCCCCCAACAACAGCAATTTCCCCAACAACAAGAATTCCCCCAACAGCAATTCCC
GCAACGACAATTCCACCAACAACAATTACCGCAACAACAATTTCCTCAACAACAATTCCCCCAACAACAA
TTCCCCCAACAACAACAGTTCCCCCAACAACAACAATTAACGCAACAACAATTCCCCCGGTCACAACAAT
CCCCTGAACAACAACAATTCCCCCAACAACAATTCCCCCAGCAACCACCACAACAATTCCCCCAACAACA
ATTTCCAATACCATACCCACCCCAGCAATCACAAGAACCTTCCCCATACCAACAATATCCACAACAACAA
CCATCTGGGAGCGACGTTATAAGTATCAGTGGCCTATGA

ω-B5* 1317 bp

ATGAAGACCTTCATCATATTTGTCCTCCTTGCCATGGCGATGAACATCGCCAGTGCCAGTAGGCTGCTAA
GCCCTAGAGGCAAGGAATTGCATACTCCACAAGAACAATTCCCCCAACAACAACAATTCCCCCAACCACA
ACAATTCCCCCAACAACAAATCCCCCAACAACATCAAATCCCCCAGCAACCACAACAATTCCCCCAACAA
CAACAATTCTTCCAACAACAACAAATCCCGCAACAACAAATCCCCCAACAACATCAAATCCCCCAGCAAC
CACAACAATTCCCCCAGCAACAGCAATTCCCCCAACAACACCAATCCCCCCAACAACAATTCCCACAACA
ACAATTCCACCAACAGAAATTGCCGCAATAGGAATTCCCACAACAACAAATCTCCCAGCAACCACAACAA
CTCCCCCAGCAATAACAAATCCCCCAGCAACCACAACAATTTCTCCAACAACAACAATTCCCCCAGCAAC
AACCCCCCAACAACATCAATTTCCCCAACAACAATTGCCCCAGCAACAACAAATCCCCCAACAACAACAA
ATCCCCCAGCAACCACAACAAATCCCCAACAACAACAAATCCCCCAGCAACCACAACAATTCCCCCAACA
ACAATTCCCGCAACAACAATTTCCCCAACAGCAATTCCCGCAACAGGAATTCCCACAACAATAACAATTC
CCGCAACAAAAAATCGCCCAGCAACCACAACAACTCCCCCAACAACAACAAATCCCCCAGCAACCACAAC
AATTTCCCCAACAACAACAATTCCCCAGCAACAATCACCCCAACAACAGCAATTTCCCCAACAACAATTC
CCCCAACAACAACAATTACCACAAAAACAATTCCCCCAACCACAACAAATACCCCAACAACAACAAATCC
CCCAGCAACCACAACAATTCCCCCAGCAACAATTCCCCCAACAACAGCAATTTCCCCAACAACAAGAATT
CCCCCAACAGCAATTCCCGCAACAACAATTCCACCAACAACAATTACCGCAACAACAATTTCCCCAACAA
CAATTCCCCCTACAGCAATTCCCCCAACAACAACAGTTCCCCCAACAACAACAATTAACGCAACAACAAT
TCCCCCGGCCACAACAATCCCCTGAACAACAACAATTCCCCCAACAACAATTCCCCCAGCAACCACCACA
ACAATTCCCCCAACAACAATTTCCAATACCATACCCACCCCAGCAATCACAAGAACCTTCCCCATACCAA
CAATATCCACAACAACAACCATCTGGGAGCGACGTTATAAGTATCAGTGGCCTATGA

ω-B6 1335 bp

ATGAAGACCTTCATCATATTTGTCCTCCTTGCCATGGCGATGAACATCGCCAGTGCCAGTAGGCTGCTAA
GCCCTAGAGGCAAGGAATTGCATACTCCACAAGAACAATTCCCCCAACAACAACAATTCCCCCAACCACA
ACAATTCCCCCAACAACAAATTCCCCAACAACATCAAATCCCCCAGCAACCACAACAATTCCCCCAACAA
CAACAATTCCTCCAACAACAACAAATCCCGCAACAACAAATCCCCCAACAACATCAAATCCCCCAGCAAC
CACAACAATTCCCCCAGCAACAATTCCCCCAACAACAGCAATTCCCCCAACAACACCAATCCCCCCAACA
ACAATTCCCACAACAACAATTCCCCCAACAGCAATTGCCGCAACAGGAATTCTCACAACAACAAATCTCC
CAGCAACCACAACAACTCCCCCAGCAACAACAAATCCCCCAGCAACCACAACAATTTCTCCAACAACAAC
AATTCCCCCAGCAACAACCCCCCCAACAACATCAATTTCCCCAACAGCAATTGCCCCAACAACAACAAAT
CCCCCAACAACAACAAATCCCCCAGCAACCACAACAAATCCCCCAACAACAACAAATCCCCCAGCAACCA
AAACAATTCCCCCAACAACAATTCCCGCAACAACAATTTCCCCAACAGCAATTCCCGCAACAGGAATTCC
CACAACAACAACAATTCCCGCAACAACAAATCGCCCAGCAACCACAACAACTCCCCCAACAACAACAAAT
CCCCCAGCAACCACAACTATTTCCCCAACAACAACAATTCCCCCAGCAACAATCACCCCAACAACAGCAA
TTTCCCCAACAACAATTCCCCCAACAACAACAATTACCGCAACAACAATTCCCCCAACCACAACAAATAC
CCCAACAACAACAAATCCCCCAGCAACCACAACAATTCCCCCAGCAACAATTCCCCCAACAACAGCAATT
TCCCCAACAACAAGAATTCCCCCAACAGCAATTCCCGCAACAACAATTCCACCAACAACAATTACCACAA
CAACAATTTCCCCAACAACAATTCCCCCAACAGCAATTCCCCCAACAACAACAGTTCCCCCAACAACAAC
AATTAACGCAACAACAATTCCCCCGGCCACAACAATCCCCTGAACAACAACAATTCCCCCAACAACAATT
CCCCCAGCAACCACCACAACAATTCCCCCAACAACAATTTCCAATACCATACCCACCCCAGCAATCACAA
GAACCTTCCCCATACCAACAATATCCACAACAACAACCATCTGGGAGCGACGTTATAAGTATCAGTGGCC
TATGA

ω-B6 445 aa

MKTFIIFVLLAMAMNIASASRLLSPRGKELHTPQEQFPQQQQFPQPQQFPQQQIPQQHQIPQQPQQFPQQ
QQFLQQQQIPQQQIPQQHQIPQQPQQFPQQQFPQQQQFPQQHQSPQQQFPQQQFPQQQLPQQEFSQQQIS
QQPQQLPQQQQIPQQPQQFLQQQQFPQQQPPQQHQFPQQQLPQQQQIPQQQQIPQQPQQIPQQQQIPQQP
KQFPQQQFPQQQFPQQQFPQQEFPQQQQFPQQQIAQQPQQLPQQQQIPQQPQLFPQQQQFPQQQSPQQQQ
FPQQQFPQQQQLPQQQFPQPQQIPQQQQIPQQPQQFPQQQFPQQQQFPQQQEFPQQQFPQQQFHQQQLPQ
QQFPQQQFPQQQFPQQQQFPQQQQLTQQQFPRPQQSPEQQQFPQQQFPQQPPQQFPQQQFPIPYPPQQSQ
EPSPYQQYPQQQPSGSDVISISGL.

ω-B7* 1369 bp

ATGAAGACCTTCATCATATTTGTCCTCCTTGCCATGGCGATGAACATCGCCAGTGCCAGTAGGCAGCTAA
GCCCTAGAGGCAAGGAATTGCATACTCCACAAGAACAATTCCCCCAACAACAACAATTCCCCCAACCACA
ACAATTCCCCCAACAACAAATCCCCAACAACATCAAATCCCCCAGCAACCACAACAATTCCCCCAACAAC
AACAATTCCTCCAACAACAAATCCCCAACAAAAATCCCCCAACAACATCAAATCCCCCAGCAACCACAAA
ATCCCCCACAACAATTCCCCCAACAACAGCAATTCCCCCAACAACACCAATCCCCCCAACAACAATTCCC
ACAACAACAATTCCCCCAACAGCAATTGCCGCAACAGGATTCCCACAACAACAAATCTCCCAGCAACCAC
AACAACTCCCCCAACAACAACAAATCCCCCAGCAACCACAACAATTTCTCCAACAACAACAATTCCCCCA
GCAACAATCCCCCCAACAACATCAATTTCCCCAACAGCAATTGCCCCAATAACAACAATTACCGCAACAA
CAATTCCCCCAACCACAACAAATCCCCCAACAACAACAAATCCCCGAGCAACCACAATAATTCCCCCAAC
AACAATTCCCGCAACAACAATTCCCCCAATAGTAATTCCCGCAACATGAATTCCCACAACAACAACAATA
CCCGCAATCACAAATCGCCCAGCAACCACAACAACTCCCCCAACAACAACAAATCCCCCAGCAACCACAA
CAATTTCCCCAACAACAACAATTCCCCAGCAACAATCCCCCCAACAACAACCCCCCCAATAACAACAATT
TCCCCAACAGCAATTCCCCCAACAACAACAATTACCGCAACAACAATTCCCCCAACCACAATAAATACCC
TAACAACAACAAATCCCCCAGCAACCACAACAATTCCCCCAACAACAATTCCCCCAACAACAGCAATTCC
CCAACAACAACAATTCCCCCAACAACATCAATTTCCCCAACAACAACAATTCCCCCAACAGTAATTCCCG
CAACAACAGTTCCACCAACAACAATTACCGCAACAACAATTTCCCCAACAACAATTCCCCCAACAGCAAT
TCCCCCAACAACAACAGTTCCCCCAACAACAACAATTACCACAACAACAATTCCCCCGGCCACAACAATC
CCCTGAACAACAACAATTCCCCCAACAACAATTCCCCCAGCAACCACCACAACAATTCCCCCAACAACAA
TTTCCAATACCATACCCACCCCAGCAATCACAAGAACCTTCCCCATACCAACAATATCCACAACAACAAC
CATCTGGGAGCGACGTTATAAGTATCAGTGGCCTATGA

ω-B8* 1457 bp

ATGAAGACCTTCATCATATTTGTCCTCCTTGCCATGGCGATGAACATCGCCAGTGCCACTAGGCAGCTAA
GCCCTAGAGGCAAGGAATTGCATACACCACAAGAACAATTCCCCCAACAACAACAATTCCCCCAACCACA
ACAATTCCCCCAATAACAAATCCCCCAACAACAATTGCCACAACAACATCAAATCTCCCAGCAACCACAA
CAAGTCCCCCAACAACAACAAATACCCCAACAACAAATCCTCGAACAACATCAAAATCCCCAGCAACCAC
AACAATTCCCCCAACAACAATTCCCCCAACAACAACAATCCCCCCAACAACAATTCCTGCAACAACAAAT
CCCCCAACAGCAATTGCCGCAACAGGAATTCCCACAACAACAAATCCCCCAACAACCACAACAACTCCCC
CGACAACAACAAATCCCCCAGCAACCAAAACAATTTCCCCAACTACAACAATTCCCCCAGCAACAATCCC
CCCAACAACAACAATTTCCCCAATAGCAATTCCTCCAAAATAACAATTATCGCAACAACAATTCCTCCAA
CCACAAAAAAATCCCCCAACAACAACAAATCCCCCAGCAACCACAACAATTCCCCCAACAACAATTCCTGC
AACAACAATTCCCCCAACAGCAATTCCCGCAACAGGAATTCCCACAACAACAACAATTCCCACAAAAACA
AATCGCCCAGCAATCACAACAACTCCCCAAACAATAACAAATCCCCCAGCAACCACAACGATTTCCCCAA
CAACAACAATTCTCCCAGCAACAATCCCCCCAACAACAATCCGCACAACAACAGCAATTTCCCCAACAAC
AATTACCGCAACAACAATTCCCCCAACCACAACAAATACCCCAACAACAACAAATCCCCCAGCAACCACA
ACAATTCCCCCAGCAACAATTCCCCCAACAACAATAATTTTCCCAACAACAACAATCCCCCCAGCAACAA
TCCCTCCAACAACAATCCCCCCAACAACAACAATTTCCCCAACGGCAATTCCCCCAACAACAACAATTCA
CCCAACCACAACAAATACCCCAACAACAACAAATCCCCCAGCAACCACAACAATTCCCCCAGCAACAATT
CCCCCGACAACATCAATTCCCCCAATAGCAATTCCCGCAACAACAGTTCCCCCAACAACAATTACCGCAA
CAACAATTTCCCCAACAACAATTCCCCGAACAACAATTCCCCCAACAAGAACAGTTCCCCCAACAACAAC
AATTACCGCAACAACAAATCCCTGAACAACAATAATTCCCCCAACAACAATTCCCCCAGCAACCATCACA

ACAATTTCCCCAACAACAATTTCCAATACCATACCCACCCCAACAATCACAAGAACCTTCCCCATACCAA
CAATATCCACAACAACAACCATATGGGAGCAACGTTATAAGTATCAGTGGCCGATGA

LMW-B1* 1065 bp

ATGAAGACCTTCCTCATCTTTGCCCTCCTCGCCGTTGCGGCGACAAGTGCCATTGCACAAATGGAGAATA
GCCACATCCCTGGTTTGGAGAGACCATCGCAGCAACAACCATTACCACCACAACAAACATTATGGCACCA
ACAACAAGAACAACCTATCCAACAACAACCACAACCATTTCCACAACAGCAACCATGTTCACAGCAACAA
CAACAACCACCATTATCGCAGCAACAACAACCACCATTTTCGCAGCAACAACAACCACCATATTCACAGC
AACAACAACCACCATTTTCACAGCAACAACCACCATTTTCACAGCAACAACAACCAGTTCTATCGCAACA
ACCACCATTTTCATAGCAACAACCACCATTTTTGCAGCAACAACAACCAGTTCTACCGCAACAACCATCA
TTTTCGCAGCAACAACTACCACCATTTTCACAGCAACAACCACCATTTTCGCAACAACAACAACCAGTAC
TACCGCAACAACCACCATTTTCGCAACAACAACAATCAGTTCTACCGCAACAACAAATACCATTTGTTCA
TCCATCTATCTTGCAGCAACTAAACCCATGCAAGGTATTCCTCCAGCAGCAATGCAGCCCTGTGGCAATG
CCACAAAGTCTTGCTAGGTCGCAAATGTTGCAGCAGAGCAGTTGCCATGTGATGCAACAACAATGTTGCC
AGCAGTTGCCGCAAATCCCCCAGCAATCCCGCTATGAGGCAATCCGTGCTATCATCTACTCCATCGTCCT
GCAAGAACAACAACAGGTTCAGGGTTCCATCCAAACTCAGCAGCAGCAACCGCAATAGTTGGGCCAATGC
GTTTCCCAACCCCAACAGCAGTCGCAGCAGCAACTCGGGCAACAACCTCAACAACAACAATTGGCACAGG
GTACCTTTTTGCATCCACACCAGATAGCTCAGCTTGAGGTGATGACTTCCATTGCGCTCCGTACCCTGCC
AACGATGTGCCGTGTCAATGTGCCCTTGTATAGAACCACCACTAGTGTGCCATTCAGCGTTGGCACCGGA
GTTGGTGCCTACTGA

LMW-B2 1113 bp

ATGAAGACCTTCCTCATCTTTGCTCTCCTCGCCATTGTGGCGACAAGTGCCATTGCACAAATGGAGAATA
GCCACATCCCTGGTTTGGAGAGACCATCGCAGCAACAACCATTACCACCACAACAAACATTATCGCACCA
CCAACAACAACAACCCATCCAACAACAACCACAACCATTTTCACAACAGCAACCATGTTCACAGCAACAA
CAACAACAACCATTATCGCAGCAACAACAACCACCATTTTCACAACAACAACCACCATTTTCGCAGCAAC
AACAACAACCATTATCGCAGCAACAACAACCACCATTTTCACAACAACAACCACCATTTTCGCAGCAACA
ACAACCACCATTTTCACAACAACAACCACCATTTTCGCAGCAACAACAACCAGTTCTACCGCAACAACCA
TCATTTTCACAGCAACAACTACCACCATTTTCACAGCAACAATCACCGTTTTCGCAACAACAACAAATAG
TACTACAGCAACAACCACCATTTTTGCAACAACAACAACCAAGTCTACCGCAACAACCACCATTTTCGCA
GCAACAACAACAACTAGTTCTACCGCAACAACAAATACCATTTGTTCATCCATCTATCTTGCAGCAGCTA
AACCCATGCAAGGTATTCCTCCAGCAGCAATGCAGCCCTGTGGCAATGCCACAAAGTCTTGCTAGGTCGC
AAATGTTGCAGCAGAGCAGTTGCCATGTGATGCAACAACAATGTTGCCAGCAGTTGCCGCAAATCCCCCA
GCAATCCCGCTATGAGGCAATCCGTGCTATCATCTACTCCATCATCCTGCAAGAACAACAACAGGTTCAG
GGTTCCATCCAAACTCCTCAGCAGCAACCCCAACAGTTGGGCCAATGTGTTTCCCAACCCCAACAGCAGT
CACAACAACAACTCGGGCAACAACCTCAACAACAACAATTGGCACAAGGTACCTTTTTGCAGCCACACCA
GATAGCTCAGCTTGAGGTGATGACTTCCATTGCGCTCCGTACCCTGCCAACGATGTGCCGTGTCAATGTG
CCGTTGTATAGAACCACCACTAGTGTGCCATTCGGTGTTGGCACCGGAGTTGGTTCCTACTAA

LMW-B2 371 aa

MKTFLIFALLAIVATSAIAQMENSHIPGLERPSQQQPLPPQQTLSHHQQQQPIQQQPQPFSQQQPCSQQQ
QQQPLSQQQQPPFSQQQPPFSQQQQQPLSQQQQPPFSQQQPPFSQQQQPPFSQQQPPFSQQQQPVLPQQP
SFSQQQLPPFSQQQSPFSQQQQIVLQQQPPFLQQQQPSLPQQPPFSQQQQQLVLPQQQIPFVHPSILQQL
NPCKVFLQQQCSPVAMPQSLARSQMLQQSSCHVMQQQCCQQLPQIPQQSRYEAIRAIIYSIILQEQQQVQ
GSIQTPQQQPQQLGQCVSQPQQQSQQQLGQQPQQQQLAQGTFLQPHQIAQLEVMTSIALRTLPTMCRVNV
PLYRTTTSVPFGVGTGVGSY.

LMW-B3 1080 bp

ATGAAGACCTTCCTCATCTTTGCCCTCCTCGCCGTTGCGGCGACAAGTGCCATTGCACAAATGGAGAATA

GCCACATCCCTGGTTTGGAGAGACCATCGCAGCAACAACCATTACCACCACAACAAACATTATCGCACCA
ACAACCACAACAACCCATCCAACAACAACCACAACAATTTCCACAACAGCAACCATGTTCACAACAACAA
CAACAACAACAACAACAACAACAACAACAACAACAACAACAACAACAACAACAACAACAACAACCATTATCGC
AGCAACAACAACCACCATTTTCACAGCAACAACAACCACCATTTTCACAGCAACAACCACCATTTTCGCA
GCAACAACAACCAGTTCTACCGCAACAGCCATCATTTTCGCAGCAACAACTACCACCATTTTCACAACAA
CAACCACCGTTTTCACAACAACAACAACCAGTACTACCGCAACAACCACCATTTTCGCAACAACAACAAC
AACAACCAATTCTACCACAACAACCATCATTTTCGCAACAACAACAACAACTAGTTCTACCGCAACAACA
AATACCATTTGTTCATCCATCTATCTTGCAGCAGCTGAACCCATGCAAGGTATTCCTCCAGCAGCAATGC
AGCCCTGTGGCAATGCCACAAAGTCTTGCTAGGTCGCAAATGTTGCAGCAGAGTAGTTGCCATGTGATGC
AACAACAATGTTGCCAGCAGTTGCCGCAAATCCCCCAGCAATCCCGCTATGAGGCAATCCGTGCTATCAT
CTACTCCATCGTCCTGCAAGAACAACAACAGGTTCGGGGTTCCATCCAAACTCAGCAGCAGCAACCGCAA
CAGTTGGGCCAATGCGTTTCCCAACCCCAACAGCAGTCACAGCAGCAACTCGGGCAACAACCTCAACAAC
AACAATTGGCACAGGGTACCTTTTTGCAGCCACACCAGATAGCTCAGCTTGAGGTGATGACTTCCATTGC
GCTCCGTACCCTGCCAATGATGTGCCGTGTCAATGTGCCGTTGTATAGGACCACCACTAGTGTGCCATTT
GGCGTTGGCACTGGAGTTGGTGCCTACTGA

LMW-B3 360 aa

MKTFLIFALLAVAATSAIAQMENSHIPGLERPSQQQPLPPQQTLSHQQPQQPIQQQPQQFPQQQPCSQQQ
QQQQQQQQQQQQQQQQQQQQPLSQQQQPPFSQQQQPPFSQQQPPFSQQQQPVLPQQPSFSQQQLPPFSQQ
QPPFSQQQQPVLPQQPPFSQQQQQQPILPQQPSFSQQQQQLVLPQQQIPFVHPSILQQLNPCKVFLQQQC
SPVAMPQSLARSQMLQQSSCHVMQQQCCQQLPQIPQQSRYEAIRAIIYSIVLQEQQQVRGSIQTQQQQPQ
QLGQCVSQPQQQSQQQLGQQPQQQQLAQGTFLQPHQIAQLEVMTSIALRTLPMMCRVNVPLYRTTTSVPF
GVGTGVGAY.

LMW-B4 1053 bp

ATGAAGACCTTCCTCATCTTTGCCCTCCTCGCCGTTGCGGCGACAAGTGCCATTGCACAAATGGAGACTA
GCCACATCCCTAGCTTGGAGAAACCATTGCAACAACAACCATTACCACTACAACAAATATTATGGTACCA
ACAACAACAACCCATCCAACAACAACCACAACCATTTCCACAACAGCCACCATGTTCACAGCAACAACAA
CCACCATTATCGCAGCAACAACAACCACCATTTTCACAACAACAACCACCATTTTCGCAGCAACAACAAC
CCATTCTACCGCAACAACCACCATTTTCGCAGCAACAACAACAATTTCCGCAGCAACAACAACCACTTTT
ACCGCAACAACCACCATTTTCGCAGCAACAACCACCATTTTCTCAGCAGCAGCAACAACCACCATTTTCG
CAGCAACAACAACAACCAATTCTACTGCAACAACCACCATTTTCACAACACCAACAACCAGTTCTACCGC
AACAACAAATACCATCTGTTCAGCCATCTATCTTGCAGCAGCTAAACCCATGCAAGGTATTCCTCCAGCA
GCAATGCAGCCCTGTGGCAATGCCACAAAGTCTTGCTAGGTCGCAAATGTTGTGGCAGAGTAGTTGCCAT
GTGATGCAGCAACAATGTTGCCGGCAGCTGCCGCAAATCCCCGAACAATCACGCTACGATGCAATCCGTG
CCATCATCTACTCGATCGTCCTACAAGAACAACAACATGGTCAGGGTTTGAACCAACCTCAGCAGCAACA
ACCCCAACAGTCGGTCCAAGGTGTCTCCCAACCCCAACAACAACAGAAGCAGCTCGGACAGTGTTCTTTC
CAACAACCTCAACAACAACAACTGGGTCAATGGCCTCAACAACAACAGGTACCCCAGGGTACCTTGTTGC
AGCCACACCAAATAGCTCAACTTGAGGTGATGACTTCCATTGCACTTCGTACCCTGCCAACGATGTGCAG
TGTCAACGTGCCGGTGTACGGCACCACCACTATTGTGCCATTCGGCGTTGGCACCCGAGTTGGTGCCTAC
TGA

LMW-B4 351 aa

MKTFLIFALLAVAATSAIAQMETSHIPSLEKPLQQQPLPLQQILWYQQQQPIQQQPQPFPQQPPCSQQQQ
PPLSQQQQPPFSQQQPPFSQQQQPILPQQPPFSQQQQQFPQQQQPLLPQQPPFSQQQPPFSQQQQQPPFS
QQQQQPILLQQPPFSQHQQPVLPQQQIPSVQPSILQQLNPCKVFLQQQCSPVAMPQSLARSQMLWQSSCH
VMQQQCCRQLPQIPEQSRYDAIRAIIYSIVLQEQQHGQGLNQPQQQQPQQSVQGVSQPQQQQKQLGQCSF
QQPQQQQLGQWPQQQQVPQGTLLQPHQIAQLEVMTSIALRTLPTMCSVNVPVYGTTTIVPFGVGTRVGAY
.

LMW_B5* 1071 bp

ATGAAGACCTTCCTCATCTTTGCCCTCCTCGCCATTGCGGCGACAAGTGCCATTGCACAAATGGAGACTA
GCCACAACCCTGGCTTGGAGAAACCATCGCAACAACAACCATTACCACTACAATAAATCTTATGGTACCA
CCAACAACAACCCATCCAACAACAACCACAACCATTTCCACAACAGCCACCATGTTCACAGCAACAACAA
CCACCATTATCGCAGCTACAACAACCACCATTTTCACAGCAACAACCACCATTATCGCAGCAACAACAAC
CAGTTCTACCGCAACAACCACCATTTTTGCAGCAACAACAACCATTTCCGCAGCAACAACAACCACCTCT
ACCGCAACAACCACCATTTTCACAGCAACAACCACCATTTTCTCAGCAGCAGCAACAACCAGTTCTACCG
CAACAACCACCATTTTCGCAACAACAACAACAACCAATTCTACCACAACAACCACCATTTTTGCAACACC
AACAACTAGTTCTACCGCAACAACAATTACCATATGTTCATCCATCTATCTTGCAGCAGCTAAACCCATG
CAAGGTATTCCTCCAGCAGCATTGCAGCCCTGTGGCAATGCCACAAAGTCTTGCTAGGTCGCAAATGTTG
TGGCAGAGCAGTTGCCATGTGATGCAGCAACAATGTTGCCAGCAGCTGCCGCGAATCCCCGAACAATCAC
GCTATGATGCAATCCGTGCCATCATCTACTCGATCGTCCTACAAGAACAACAACCTGGTCAGGGTTTCAA
CCAACCTTAGCAGCAACAACCCCAACAGTCGGTCCAAGGTGTCTCCCAACCCCAACAACAACAGAAGCAA
CTCGGACAATGTTCTTTCCAACAACCACAACAACAACAACTCGGTCAATGGCCTCAACAACAACAGGTAC
CACAGGGTACCTTATTGCAGCCACACCAAATAGCTCAACTTGAGGTGATGACTTCCATTGCATTCCATAC
CCTGCCAACGTTGTGCAGTACCAATGTGCCGGTGTACGGCACCACCACTAGTGTGCCATTCGGCGTTGGC
ACCCAAGTTGGTGCCTACTGA

δ-D1 975 bp

ATGAAGATCTTCATGGTCTTTGCCCTCCTCGTTGCATCAACGACCATCACCACCGCGACCGCACAGCTCG
ACCCTCGCATCCATGACCAAGAAAGGCCACAACAATCGTTTCTGCAACAGCAACCACTTATCCAGCAACA
ACCATACCCGCCTCAAGAGCCACAACAACCACTATTCCCGCAAAAAGAGCCACAACAACCATTTCCGCTG
CAGCAGCCACAATACCAGCAACAACAACCGTATCCACAACAACCACTTCCCCAAGAACAACTTCCCCAGC
AACATTTATTTCCGCAGCAGCCGCCACAACAACAATTTCCACAACAGATGCCACTTCCGCATCAACAACA
AACATTCCCGCAACAACAACAACAACAAGAACAACTCCCACAACAACAACCACAATTCCCGCAACAACAA
CCATTTTCCCAATATCAACAACCATTAACACAACAACCATACTCGCAAGAGCAACCATTGCCACAACAAC
AACCTTCTGTAGAGGAAAAACAACAATTGAACTTGTGCAAGGAGTTCCTCCTGCAGCAGTGTAACCCAGA
GGAGAAACTGTCGTTACTCCAGTCAGTGATCCCGTTCCTCCGACCAAAGACCTCGCAACAGAACAGCTGC
CAGTTGAAGCGACTACAATGTTGTCGACAACTTGCACATATCAGTGAACCGTCCCGATGCCCGGCCATCC
ACAACATTGTGCATGCCATCGTCATGCAACAACAACATGTGGATAGAGGTTTCGGCCAGCCTCAACCACA
ACAGTTGGGCCAGGAAATGCCCATGCAGCCTCAACATCAATTGGGCCAGCACTCTATCCTACCTCAACAA
CTAGCCCAGTACCAGTTGGTTAGGTTACTTGTGATTCAGACCCTTCCTATGTTATGCAACGTGCATGTCC
CGTCTGATTGCTACACCATTACTGCACCATTTGGTAGCATGACTGCCTACAATGGTGGACAATGA

δ-D1 325 aa

MKIFMVFALLVASTTITTATAQLDPRIHDQERPQQSFLQQQPLIQQQPYPPQEPQQPLFPQKEPQQPFPL
QQPQYQQQQPYPQQPLPQEQLPQQHLFPQQPPQQQFPQQMPLPHQQQTFPQQQQQQEQLPQQQPQFPQQQ
PFSQYQQPLTQQPYSQEQPLPQQQPSVEEKQQLNLCKEFLLQQCNPEEKLSLLQSVIPFLRPKTSQQNSC
QLKRLQCCRQLAHISEPSRCPAIHNIVHAIVMQQQHVDRGFGQPQPQQLGQEMPMQPQHQLGQHSILPQQ
LAQYQLVRLLVIQTLPMLCNVHVPSDCYTITAPFGSMTAYNGGQ.

δ-D2* 284 bp

ATGAAGATCTTCTTGCTCTTTGCCCTTCTGGTTGCCACAACGATCATCACCACCACCACTGCGCAGCTTG
TCCCTAATGCCCATGACTTAGAAAGGCCACAACAATCATTTCCGCAACAGCAACCACTTCCCCGGCTACA
ACCATTCCCGCCGCAAGAGCCACAACAACTCCCCCCCCCCCCCAAAAAGAGCCACAACAATCATTTCCAC
TGCAGCACCCACAACCCCAGCAACAAGAACCACTTCCCCAACAACATCTATTTCCGCAGCAACCGCCACA
ATAA

γ-D1 909 bp

ATGAAGACCTTACTCATCGTAACAATCCTTGCGATGGCAACAACCATCGCCACCGCCAATATGCAAGTCG
ACCCCGGCTACCAAGTACATTGGCCACAACAACAACCATTCCCCAGCCCCAACAACCATTCTGCCAGCA
ACCACAACAAACTATTCCCCAACCCCATCAAACGTTCCACCATCAACCACAACAAACATTTCCCCAACCC
CAACAAACATACCCCCATCAACCACAACAACAATTTCCCCAGACCCAGCAACCACAACAACCATTTCCCC
AGCCCCAACAAACATTCCCCCAACAACCCCAACTACCATTTCCCCAACAACCCCAACAACCATTCCCCCA
GCCTCAACAACCCCAACAACAATTTCCCCAGTCACAGCAACCACAACAACCTTTTCCCCAGCCCCAACAA
CAATTCCTGCAGCCCCAACAACCGCAACAATCATTCCCCCAGCAACAACAACCGTTGATTCAGCTATCTC
TACAACAACAGATGAACCCCTGCAAGAATTTTCTCTTGCAGCAATGCAACCCTGTGTCATTGGTGTCATC
CCTCATATCAATGATCTTGCCACGAAGTGATTGCCAGGTGATGCAGCAACAATGTTGCCAACAACTGGCA
CAGATTCCTCAGCAGCTCCAGTGTGCAGCCATCCATAGTGTCGTGCATTCCATCATCATGCAGCAAGAAC
AACGACAAGGCGTGCAGATCCGGCGGCCACTGTTTCAGCTCGTTCAGGGTCAGGGCATCATCCAACCTCA
ACAACCAGCTCAATTGGAGGTGATCAGGTCATTGGTATTGAGAACTCTTCCAACCATGTGCAACGTGTAT
GTCTCACCTGACTGCTCCACCATCAACGCACCATTTGCCAGCATAGTCGTCGGCATTGGTGGCCAATGA

γ-D1 303 aa

MKTLLIVTILAMATTIATANMQVDPGYQVHWPQQQPFPQPQQPFCQQPQQTIPQPHQTFHHQPQQTFPQP
QQTYPHQPQQQFPQTQQPQQPFPQPQQTFPQQPQLPFPQQPQQPFPQPQQPQQQFPQSQQPQQPFPQPQQ
QFLQPQQPQQSFPQQQQPLIQLSLQQQMNPCKNFLLQQCNPVSLVSSLISMILPRSDCQVMQQQCCQQLA
QIPQQLQCAAIHSVVHSIIMQQEQRQGVQIRRPLFQLVQGQGIIQPQQPAQLEVIRSLVLRTLPTMCNVY
VSPDCSTINAPFASIVVGIGGQ.

γ-D2 984 bp

ATGAAGACCTTACTCATCCTGACAATCCTTGCGATGGCAATAACCATCGGCACCGCCAATATCCAGGTCG
ACCCTAGCGGCCAAGTACAATGGCTACAACAACAACTAGTCCCCCAGCTCCAACAGCCATTATCCCAGCA
ACCACAACAAACATTTCCCCAACCTCAACAAACATTCCCCCATCAACCACAACAACAAGTTCCCCAGCCT
CAGCAACCACAACAACCATTTCTCCAGCCCCAACAACCATTCCCCCAACAACCACAACAACCATTCCCCC
AGACTCAACAACCACAACAACCATTTCCCCAGCAACCACAACAACCATTTCCCCAGACTCAACAACCCCA
ACAACCATTTCCCCAACAACCACAACAACCATTCCCCCAGACTCAACAACCCCAACAACCATTTCCCCAG
CTCCAGCAACCACAACAACCTTTTCCCCAGCCCCAACAACAATTACCGCAGCCCCAACAACCGCAACAAT
CATTCCCCCAACAACAACGGTCATTCATTCAACCATCTCTACAACAACAGTTGAACCCATGCAAGAATAT
CCTCTTGCAACAATGCAAACCTGCGTCATTGGTGTCATCCCTCTGGTCAATAATCTGGCCACAAAGCGAT
TGCCAAGTGATGCGGCAACAATGCTGCCAACAACTAGCACAGATTCCTCAACAGCTCCAGTGCGCAGCCA
TCCATAGCGTCGTGCATTCCATCATCATGCAGCAGCAGCAACAACAACAACAACAACAAGGCATGCATAT
CTTTCTGCCACTATCTCAGCAGCAACAGGTGGGTCAAGGTTCTCTAGTCCAAGGCCAGGGCATCATCCAA
CCACAACAACCAGCTCAATTGGAGGCGATCAGATCATTGGTATTGCAAACTCTTCCATCCATGTGCAACG
TGTATGTCCCACCAGAGTGCTCCATCATGAGGGCACCATTTGCCAGCATAGTCGCGGGCATTGGTGGCCA
ATGA

γ-D2 328 aa

MKTLLILTILAMAITIGTANIQVDPSGQVQWLQQQLVPQLQQPLSQQPQQTFPQPQQTFPHQPQQQVPQP
QQPQQPFLQPQQPFPQQPQQPFPQTQQPQQPFPQQPQQPFPQTQQPQQPFPQQPQQPFPQTQQPQQPFPQ
LQQPQQPFPQPQQQLPQPQQPQQSFPQQQRSFIQPSLQQQLNPCKNILLQQCKPASLVSSLWSIIWPQSD
CQVMRQQCCQQLAQIPQQLQCAAIHSVVHSIIMQQQQQQQQQGMHIFLPLSQQQQVGQGSLVQGQGIIQ
PQQPAQLEAIRSLVLQTLPSMCNVYVPPECSIMRAPFASIVAGIGGQ.

γ-D3 888 bp

ATGAAGACCTTACTCATCCTAACAATCCTTGCGATGGCAATAACCATCGGCACCGCCAATATGCAGGTCG

ACCCTAGCAGCCAAGTACAATGGCCACAACAACAACCAGTCCCACAGCCTCACCAACCATTCTCCCAGCA
ACCACAACAAACATTTCCCCAACCCCAACAAACATTCCCCCATCAACCACAACAACAATTTCCCCAGCCT
CAGCAACCACAACAACAATTTCTCCAGCCCCAACAACCATTCCCCCAACAACCACAACAACCATATCCCC
AGCAACCACAACAACCATTCCCCCAGACTCAACAACCCCAACAACTATTTCCCCAGTCCCAGCAACCACA
ACAACAATTTTCTCAGCCCCAACAACAATTCCCGCAGCCCCAACAACCGCAACAATCATTCCCCCAACAA
CAACCACCGTTCATTCAGCCATCTCTACAACAACAGGTGAACCCATGCAAGAATTTCCTCTTGCAGCAAT
GCAAACCTGTGTCACTGGTGTCATCCCTCTGGTCAATGATCTGGCCACAAAGCGATTGCCAAGTGATGCG
GCAACAATGCTGCCAACAACTAGCACAGATTCCTCAGCAGCTCCAGTGTGCAGCCATCCATACCGTCATA
CATTCCATCATCATGCAGCAAGAACAACAACAAGGCATGCATATCCTGCTGCCACTATATCAGCAGCAAC
AGGTGGGTCAAGGTACTCTCGTCCAGGGCCAGGGCATCATCCAACCCCAACAACCAGCTCAATTGGAGGC
GATCAGGTCATTGGTGTTGCAAACTCTTCCAACCATGTGCAACGTGTATGTCCCACCTGAGTGCTCCATC
ATCAAGGCACCATTTTCCAGCGTAGTCGCCGGCATTGGTGGCCAATGA

γ-D3 296 aa

MKTLLILTILAMAITIGTANMQVDPSSQVQWPQQQPVPQPHQPFSQQPQQTFPQPQQTFPHQPQQQFPQP
QQPQQQFLQPQQPFPQQPQQPYPQQPQQPFPQTQQPQQLFPQSQQPQQQFSQPQQQFPQPQQPQQSFPQQ
QPPFIQPSLQQQVNPCKNFLLQQCKPVSLVSSLWSMIWPQSDCQVMRQQCCQQLAQIPQQLQCAAIHTVI
HSIIMQQEQQQGMHILLPLYQQQQVGQGTLVQGQGIIQPQQPAQLEAIRSLVLQTLPTMCNVYVPPECSI
IKAPFSSVVAGIGGQ.

γ-D4 897 bp

ATGAAGACCTTACTCATCCAAACAATCCTCGTGATGGCAATAACCATCGCCACCGCCAATATGCAGGTCG
ACCCTAGCGGCCAAGTACCATGGCCACAACAACAACCATTCCCGCAGCCTCACCAACCATTCTCCCAGCA
ACCGCAACAAACATTTCCCCAACCCCAACAAACATTCCCCCATCAACCACAACAACAATTTTCCCAGCCT
CAGCAACCACAACAACAATTTATCCAGCCCCAACAACCATTCCCCCAACAACCACAACAAACATATCCCC
AGCGACCACAACAACCATTCCCCCAGACTCAACAACCCCAACAACCATTTCCCCAGTCCCAGCAACCACA
ACAACCTTTTCCCCAGCCCCAACAACAATTCCCGCAGCCCCAACAACCACAACAATCATTCCCCCAACAA
CAACCATCGTTGATTCAACAATCTCTACAACAACAGTTGAACCCATGCAAGAATTTCCTCTTGCAACAAT
GCAAACCTGTGTCCTTGGTGTCATCCCTCTGGTCAATGATCTTGCCACGAAGCGATTGCCAGGTGATGCG
GCAACAATGTTGCCAACAACTAGCACAAATTCCTCAGCAACTCCAGTGTGCAGCCATCCATAGCATCGTG
CATTCCATCATCATGCAGCAAGAACAACAAGAACAACGACAGGGTGTGCAAATCCTGGTGCCACTGTCTC
AACAGCAACAGGTAGGTCAAGGTACTCTCGTCCAAGGTCAGGGCATCATCCAACCTCAACAACCAGCTCA
ATTGGAGGTGATTAGGTCATTGGTGTTGCAAACTCTTGCAACCATGTGCAACGTGTATGTCCCACCTTAC
TGCTCCACCATCAGGGCACCATTTGCCAGCATAGTCGCCGGCATTGGTGGCCAATGA

γ-D4 299 aa

MKTLLIQTILVMAITIATANMQVDPSGQVPWPQQQPFPQPHQPFSQQPQQTFPQPQQTFPHQPQQQFSQP
QQPQQQFIQPQQPFPQQPQQTYPQRPQQPFPQTQQPQQPFPQSQQPQQPFPQPQQQFPQPQQPQQSFPQQ
QPSLIQQSLQQQLNPCKNFLLQQCKPVSLVSSLWSMILPRSDCQVMRQQCCQQLAQIPQQLQCAAIHSIV
HSIIMQQEQQEQRQGVQILVPLSQQQQVGQGTLVQGQGIIQPQQPAQLEVIRSLVLQTLATMCNVYVPPY
CSTIRAPFASIVAGIGGQ.

ω-D1 1134 bp

ATGAAGACCTTCCTCATCTTTGTCCTCCTTGCCATGGCGATGAACATCGCCACTGCTGCTAGGCAGCTAA
ACCCTAGCAACAAAGAGCTACAATCACCTCAACAATCATTTTCCCATCAACAACAACCATTTCCACAGCA
ACCATATCCACAACAACCATATCCATCACAGCAACCATATCCATCGCAACAACCATTTCCCACACCCCAA
CCACAATTTCCCCAGCAATCACAACAACCATTTACCCAGCCCCAACAACCGACCCCCCTACAACCACAAC
AACCATTCCCCCAGCAACCCCAACAACCACAACAACCTTTTCCACAGCCGCAACAACCATTTCCCTGGCA
ACCACAACAACCATTTCCCCAGACCCAACAATCTTTCCCTCTGCAACCACAACAACCATTCCCCCAGCAA

CCCCAACAACCATTTCCCCAGCCCCAACTACAATTCCCCCAGCAACCAGAACAAATAATTCCCCAGCAAC
CCCAACAACCATTCCTCCTGGAATCGCAACAACCATTCCCCCAGCAACCCCAACAACCATTTCCCCAGCC
CCAACAACTGATCCCCATGCAACCACAACAACCATTCCCCCAGCAATCCCAACAATCACAACAACCTTTT
CCCGGGCCCCAACAATTATTTCCTGAACTCCAACAACCAATTCCCCAGCAACCACAACAACCATTCCCCC
TGCAACCGCAACAACCATTCCCCCAGCAATCGCAACAACCATTCCCCCAGCAACCCCAACAACCATGCCC
CCTGCAACCGCAACAACCATTCCCCCAACAACCACAACAACCATTCCCCCAACAACCACAACAACCTTTC
CCCCTACAACCACAACAACCATTCCCCCTACGACCGCAACAACCATTTTCCCAGCAACCCCAACAATCAC
AACAATCATTTCCCCAGCCCCAACCCCAGCAACCCCAACAACCATCCATCCTGCAACCACAACAACCATT
TCTGCAGCCCCAACAACAATTATCCCAGCAACTAGAACAAACAATTTCCCAGCAACCCCAACAACCATTC
CCCCAGCAACCACACCAACCTCAACAACCATATCCACAACAACAACCATATGGGAGTAGTCTTACAAGCA
TCGATGGCCAATGA

ω-D1 378 aa

MKTFLIFVLLAMAMNIATAARQLNPSNKELQSPQQSFSHQQQPFPQQPYPQQPYPSQQPYPSQQPFPTPQ
PQFPQQSQQPFTQPQQPTPLQPQQPFPQQPQQPQQPFPQPQQPFPWQPQQPFPQTQQSFPLQPQQPFPQQ
PQQPFPQPQLQFPQQPEQIIPQQPQQPFLLESQQPFPQQPQQPFPQPQQLIPMQPQQPFPQQSQQSQQPF
PGPQQLFPELQQPIPQQPQQPFPLQPQQPFPQQSQQPFPQQPQQPCPLQPQQPFPQQPQQPFPQQPQQPF
PLQPQQPFPLRPQQPFSQQPQQSQQSFPQPQPQQPQQPSILQPQQPFLQPQQQLSQQLEQTISQQPQQPF
PQQPHQPQQPYPQQQPYGSSLTSIDGQ.

ω-D2 1158 bp

ATGAAGACCTTCCTCATCTTTGTCCTCCTTGCCATGGCGATGAAGATCGCCACTGCCGCTAGGGAGTTAA
ACCCTAGCAACAAAGAGTTACAATCACCTCAACAATCATTTTCCCATCAACAACAACCATTTCCACAGCA
GCCATATCCACAACAACCATATCCATCACAGCAACCATATCCATCGCAACAACCATTTCCCACACCCCAA
CAACAATTTCCCCAGCAATCACAACAACCATTTACCCAGCCCCAACAACCGACCCCCTTACAACCACAAC
AACCATTCCCCCAGCAACCCCAACAACCACAACAACCTTTTCCACAACCCCAACAACCATTTCCCTGGCA
ACCACAACAACCATTTCCCCAGACCCAACAATCGTTCCCTCTCCAACCACAACAGCCATTCCCCCAGCAA
CCCCAACAACCATTTCCCCAGCCCCAACTACCATTCCCCCAGCAATCAGAACAAATAATTCCCCAGCAAC
CCCAACAACCATTCCCCCTGCAACCGCAACAACCATTCCCCCAGCAACCCCAACAACCATTTCCCCAGCC
CCAACAACCAATCCCCGTGCAACCACAACAATCATTCCCCCAACAATCCCAACAATCACAACAACCTTTT
GCCCAGCCCCAACAATTATTTCCTGAACTCCAACAACCAATTCCCCAGCAACCACAACAACCATTCCCCC
TGCAACCGCAACAACCATTCCCCCAGCAACCGCAACAACCATTCCCCCAGCAACCGCAACAATCATTTCC
CCAGCAACCACAACAACCATTCCCCCAGCAACCGCAACAACCATTCCCCCAACAACCACAACAACCATTC
CCTCAACAACCACAACAACCATTCCCCCTACGACCGCAACAACCATTTCCCCAGCAACCCCAACAATCAC
AACAATCATTTCCCCAGCCCCAACCCCAGCAACCCCAACAACCATCCATCCTTCAACCACAACAACCATT
ACCCCAACAACCACAACAACCATTTCAACAGCCCCAACAACAATTATCCCAGCAACCAGAACAAACAATT
TCCCAGCAACCCCAACAACCATTCCCCCAGCAACCACACCAACCTCAACAACCATATCCACAACAACAAC
CATATGGGAGTAGTCTTACAAGCATCGGTGGCCAATGA

ω-D2 386 aa

MKTFLIFVLLAMAMKIATAARELNPSNKELQSPQQSFSHQQQPFPQQPYPQQPYPSQQPYPSQQPFPTPQ
QQFPQQSQQPFTQPQQPTPLQPQQPFPQQPQQPQQPFPQPQQPFPWQPQQPFPQTQQSFPLQPQQPFPQQ
PQQPFPQPQLPFPQQSEQIIPQQPQQPFPLQPQQPFPQQPQQPFPQPQQPIPVQPQQSFPQQSQQSQQPF
AQPQQLFPELQQPIPQQPQQPFPLQPQQPFPQQPQQPFPQQPQQSFPQQPQQPFPQQPQQPFPQQPQQPF
PQQPQQPFPLRPQQPFPQQPQQSQQSFPQPQPQQPQQPSILQPQQPLPQQPQQPFQQPQQQLSQQPEQTI
SQQPQQPFPQQPHQPQQPYPQQQPYGSSLTSIGGQ.

ω-D3 1158 bp

ATGAAGACCTTCCTCATCTTTGTCCTCCTTGCCATGGCGATGAAGATCGCCACTGCCGCTAGGGAGTTAA
ACCCTAGCAACAAAGAGTTACAATCACCTCAACAATCATTTTCCCATCAACAACAACCATTTCCACAGCA
GCCATATCCACAACAACCATATCCATCACAGCAACCATATCCATCGCAACAACCATTTCCCACACCCCAA
CAACAATTTCCCCAGCAATCACAACAACCATTTACCCAGCCCCAACAACCGACCCCCTTACAACCACAAC
AACCATTCCCCCAGCAACCCCAACAACCACAACAACCTTTTCCACAACCCCAACAACCATTTCCCTGGCA
ACCACAACAACCATTTCCCCAGACCCAACAATCGTTCCCTCTCCAACCACAACAGCCATTCCCCCAGCAA
CCCCAACAACCATTTCCCCAGCCCCAACTACCATTCCCCCAGCAATCAGAACAAATAATTCCCCAGCAAC
CCCAACAACCATTCCCCCTGCAACCGCAACAACCATTCCCCCAGCAACCCCAACAACCATTTCCCCAGCC
CCAACAACCAATCCCCGTGCAACCACAACAATCATTCCCCCAACAATCCCAACAATCACAACAACCTTTT
GCCCAGCCCCAACAATTATTTCCTGAACTCCAACAACCAATTCCCCAGCAACCACAACAACCATTCCCCC
TGCAACCGCAACAACCATTCCCCCAGCAACCGCAACAACCATTCCCCCAGCAACCGCAACAATCATTTCC
CCAGCAACCACAACAACCATTCCCCCAGCAACCGCAACAACCATTCCCCCAACAACCACAACAACCATTC
CCTCAACAACCACAACAACCATTCCCCCTACGACCGCAACAACCATTTCCCCAGCAACCCCAACAATCAC
AACAATCATTTCCCCAGCCCCAACCCCAGCAACCCCAACAACCATCCATCCTTCAACCACAACAACCATT
ACCCCAACAACCACAACAACCATTTCAACAGCCCCAACAACAATTATCCCAGCAACCAGAACAAACAATT
TCCCAGCAACCCCAACAACCATTCCCCCAGCAACCACACCAACCTCAACAACCATATCCACAACAACAAC
CATATGGGAGTAGTCTTACAAGCATCGGTGGCCAATGA

ω-D3 386 aa

MKTFLIFVLLAMAMKIATAARELNPSNKELQSPQQSFSHQQQPFPQQPYPQQPYPSQQPYPSQQPFPTPQ
QQFPQQSQQPFTQPQQPTPLQPQQPFPQQPQQPQQPFPQPQQPFPWQPQQPFPQTQQSFPLQPQQPFPQQ
PQQPFPQPQLPFPQQSEQIIPQQPQQPFPLQPQQPFPQQPQQPFPQPQQPIPVQPQQSFPQQSQQSQQPF
AQPQQLFPELQQPIPQQPQQPFPLQPQQPFPQQPQQPFPQQPQQSFPQQPQQPFPQQPQQPFPQQPQQPF
PQQPQQPFPLRPQQPFPQQPQQSQQSFPQPQPQQPQQPSILQPQQPLPQQPQQPFQQPQQQLSQQPEQTI
SQQPQQPFPQQPHQPQQPYPQQQPYGSSLTSIGGQ.

ω-D4* 1163 bp

ATGAAGACCTTCATCATATTTGTCCTCCTTGCTATGGTGATGAGCATCGCCAGTGCCACTAGGCAACTAA
GCCCTAGAGGCAAGGAGTTGCAAACACCACAAGAACAATTCCCCCAACAGCAATTCCCTCAACCACAACA
AATCCTCCAACAACAATTCCCCCAACGACAACAAATCCCCCAGCAACCGCAACAATTCCCCCAACAGCAA
TTCCCGCAACAGCAAATCCCACAACAGCAAATCCCGCAACAACAATTCCCCCAACCACAACAAATCCCCC
AACAACAATTCCCCCAACAACAACAAATCCCCCAACAACAATTCCCCCAACAACAACAAATCCCCCAGCA
ACCACAACAATTCCCCCAACAACAGCAATTCCCCCAACAACAACAAATCCCCCAGCAACCACAACAATTT
CCCCAACAACAGCAATTCCCCCAACAACAACAATTCCCCCAACAGCAATTCCCGCAACAACAATTCCCCG
AACAACAATTCTCACAACAACAATCCCCCCAACCACAACAAATCCCCCAACAACAATTACTCCAACAACA
ACAAATCCCCCAGCAAGAATTCCCCCAACAACAGCAATTCCCGCAACAACAATTCCCCCAACAGCAATTC
CCTCAACAGCAATTCCCACAACAACAACAAATCCCCCAGCAACAACCACAACAACATCCCCAACAACAAC
AACAATTTCCCCAACAACAACAAATCCCCCAACAAAAATACCCCCAACAACAACAATTCCCCCAACAGCA
ATTCCCCCAACAACAACAATTTCCCCAACAACACCAGTTACCGCAACAACAATTCCCCCAACAACAACAA
ATCTCCGAGCAACAACAACAATTCCCCCAACAACAACAATTCCCCCAACAGCAATTCCCGCAACAACAAT
TTCCCCAACAACAACAATTCCCACAACAACAACAGTTCCCCCAACAACAACAATTCCCCCAACAACAACA
GTTCCCCCAACAACAACAATTCCCGCAACAACAATTCCCCCAGCAACCACAACAACAATTCCCAAAACAA
CAATTTCCCATACCATACCCACCCCAACAACAATCGCAAGAAGCTTCCCCATACCAACAATACCACAACA
ACAACTATCTGGGAGCGACATTATAAGTATCAGTGGCCGATGA

ω-D5* 1165 bp

ATGAAGACCTTCATCATATTTGTCCTCCTTGCTATGGTGATGAGCATCGCCAGTGCCACTAGGCAACTAA
GCCCTAGAGGCAAGGAGTTGCAAACACCACAAGAACAATTCCCCCAACAGCAATTCCCTCAACCACAACA
AATCCTCCAACAACAATTCCCCCAACAACAACAAATCCCCCAGCAACCGCAACAATTCCCCCAACAGCAA
ATCCCACAACAGCAAATCCCGCAACAACAATTCCCCCAACCACAACAAATCCCCCAACAACAATTCCCCC

AACAACAACCAATCCCCCAGCAACCACAGCAATTCCCCCAACAACAACAAATCCCCCAGCAACCACAACA
ATTCCCCCAACAACAGCAATTCCCCCAACAACAACAATTCCCCCAACAGCAATTCCCGCAACAACAATTC
CCCGAACAACAATTCTCACAACAACAATCCCCCCAACCACAACAAATCCCCCAACAACAATTACTCCAAC
AACAACAAATCCCCCAACAACAATTACTCCAACAACAACAAATCCCCAGCAAGAATTCCCCCAACAACAG
CAATTCCCGCAACAACAATTCCCCCAACAGCAATTCCCACAACAACAACAAATCCCCCAGCAACAACCAC
AACAACTTCCCCAACAACAACAACAATTTCCCCAACAACAACAATTCCCCCAACAACAATACCCCCAACA
ACAACAATTCCCCCAACAGCAATTCCCCCAACAACAACAATTTCCCCAACAACACCAGTTACCGCAACAA
CAATTCCCCCAACAACAATTCCCTCAACAACAACAAATCTCCGAGCAACCACAACAATTCCCCCAACAAC
AACAATTCCCCCAACAACAACAAATCCCCCAACAGCAATTCCCGCAACAACAATTTCCCCAACAACAACA
ATTCCCACAACAACAACAGTTCCCCCAACAACAACAATTCCCCCATCCACAACAATTCCCCCAACAACAA
CAGTTCCCCCAACAACAACAATTCCCCCAACAACAATTCCCCCAGCAACCACAACAACAATTCCCCCAAC
AACAATTTCCCATACCATACCCACCCCAACAACAATCACAAGAAGCTTCCCCATACCAACAATACCACAA
CAACACCTATCTGGGAACGACGTTATAAGTATCAGTGGCCGATGA

ω-D6* 1513 bp

ATGAAGACCTTCATCATATTTGTCCTCCTTGCTATGGTGATGAGCATCGCCAGTGCCACTAGGCAACTAA
GCCCTAGAAGCAAGGAGTTGCAAACACCACAAGAACAATTCCCCCAACAGCAATTTCCTCAACCACAACA
AATCCTCCAACAACAATTCCCCCAACAACAACAAATCCCCCAGCAACCGCAACAATTCCCCCAACAACAA
ATCCCGCAACAGCAAATCCCGCAACAACAAATCCCCCAACAACAACAAATCCCCCAGCAACCACAACAAT
TCCCCCAACAACAGCAATTCCCCCAACAACAACAAATCCCCCAGCAACCACAACAATTCCCCCAACAACA
GCAATTCCCCCAACAACAACAAATCCTCCAGCAACCACAACAATTCCCCCAACAACAGCAATTCCCCCAA
CAACAACAAATCCCTCAGCAACCACAACAATTCCCCCAACAAAAGCAATTCCCCCAACAACAACAAATCC
CTCAGCAACCACAACAATTCCCCCAACAACAACAAATCCCTCAGCAACCACAACAATTCCCCCAACAAAA
GCAATTCCCCCAACAACAACAAATCCCCCAGCAACCACAACAATTCCCCCAACAACAGCAATTCCCCCAA
CAACAACAAATCCTCCAGCAACCACAACAATTCCCCCAACAACAACAATTCCCCCAACAACAACAAATCC
CTCAGCAACCACAATAATTCCCCCAACAAAAGCAATTCCCCCAACAACAACAAATCCCCCAGCAACCACA
ACAATTCCCCCAACAACAGCAATTCCCCCAACAACAATTCCCGCAACACCAATTCCCCCAACAGCAATTC
CCACAACAACAATCCCCCCAACCACAACAAATCCCCCAACAACAATTACTCCAACAACAAATCCCCCAGC
AACAATTCCCCCAACAACAGCAATTCCCGCATCAACAATTCCCCCAACAGCAATTTCCTCAACAGCAATT
CCCACAACAACAACAAATCCCCCAGCAACAACCACAACAACTCCCCCAACAACAACAATTTCCCCAACAA
CAACAATTACCCCAACAACAATACCCCGAACAACAACAATTCCCCCAACAGCAATTCCCCCAACAACAAC
AATTTCCCCAACAACACCAGTTACCGCAACAACAATTCCCCCAACAACAACAAATCTCCGAGCAACCACA
ACAATTCCCCCAACAACAACAATTCCCCCAACAACAACAATTCCCCCAACAGCAATTCCCGCAACAACAA
TTTCCCCAACAACAACAATTCCCACAACAACAACAGTTCCCTCAACAACAACAATTCCCCAACAACAACA
GTTCCCCCAACAACAACAATTCCCCCAACAACAATTCCCCCAACAACCACAACAACAATTCCCCCAACAA
CAATTTCCCATACCATACCCACCCCAACAACAATCACAAGAAGCTTCCCCATACCAACAATACCACAACA
ACAACTATCTGGGAGCGACGTTATAAGTATCAGTGGCCGATGA

ω-D7* 330 bp

ATGTTTGTCTCTTCATTTATATAGAAAAAATAGCATGTAAATTTTTAATCCACATTTCTTGCCATCATGG
TACAGATGCAAAATTGCCATGAACTTTTTCTTTTTTGCCATCAAAAATTACACAAACAATAATTCCCCCA
ACAACAACAATTACCGCAACAACAATTGCGCCAACAACAAAAGTTCCCCCAACTGCAACAATTCCCCAAG
CAACCACAACAACAATTTCCCATACCATACCCACCCCAACAACCATTCCCCCAGCAACAACAACCTTCCC
CACACCAACAACCAACTGGGAGCGATGTTATAAGTATCAGTGGCCGATGA

LMW-D1 1065 bp

ATGAAGACCTTCCTCATCTTTGCCCTCCTCGCCGTTGCGGCAACAAGTGCCATTGCACAAATGGAGAATA
GCCACATCCCTGGTTTGGAGAAACCATCGCAGCAACAACCATTACCACTGCAACAAACATTATCGCACCA
ACAACAACAACAACCTGTCCAACAACAACCACAACCATTTCCACAACAGCAACCATGTTCACAGCAACAA
CAACCACCATTATCGCAGCAACAACAACCACCATTTTCACAACAACAACCACCATTTTCGCAGCAACAAC
AACCATCATTTTCGCAGCAACAACAACCACCATTTTCACAGCAACAACCACCATTTTCGCAGCAACAACA

ACCAGTTCTACCGCAACAACCATCATTTTCGCAGCAACAACTACCACCATTTTCACAGCAACAACCACCG
TTTTCGCAACAACAACAACCAGTACTACCGCAACAACCACCATTTTCGCAACAACAACAACCAATTCTAC
CGCAACAACCACCATTTTCGCAACAACAACAACAACCAGTTCTACCGCAACAACAAATACTATTTGTTCA
TCCATCTATCTTGCAGCAGCTAAACCCATGCAAGGTATTCCTCCAGCAGCAATGCAGCCCTGTGGCAATG
CCACAAAGTCTTGCTAGGTCGCAAATGTTGCAGCAGAGCAGTTGCCATGTGATGCAACAACAATGTTGCC
AGCAGTTGCTGCAAATCCCCCAGCAATCCCGCTATGAGGCAATCCGTGCTATCATCTACTCCATCATCCT
GCAAGAACAACAACAGGTTCAGGGTTCCATCCAAACTCAGCAGCAGCAACCCCAAGAGTTGGGCCAATGT
GTTTCCCAACCCCAACAGCAGTCGCAGCAGCAACTCGGGCAACAACCTCAACAACAACAATTGGCACAGG
GTACCTTTTTGCAGCCACACCAGATAGCTCAGCTTGAGGTGATGACTTCCATTGCGCTCCGTACCCTACC
AACGATGTGCCGTGTCAATGTGCCGTTGTATAGAACCACCACTAGTGTGCCATTCGGCGTTGGCGCCGGA
GTTGGTGCCTACTGA

LMW-D1 355 aa

MKTFLIFALLAVAATSAIAQMENSHIPGLEKPSQQQPLPLQQTLSHQQQQQPVQQQPQPFPQQQPCSQQQ
QPPLSQQQQPPFSQQQPPFSQQQQPSFSQQQQPPFSQQQPPFSQQQQPVLPQQPSFSQQQLPPFSQQQPP
FSQQQQPVLPQQPPFSQQQQPILPQQPPFSQQQQQPVLPQQQILFVHPSILQQLNPCKVFLQQQCSPVAM
PQSLARSQMLQQSSCHVMQQQCCQQLLQIPQQSRYEAIRAIIYSIILQEQQQVQGSIQTQQQQPQELGQC
VSQPQQQSQQQLGQQPQQQQLAQGTFLQPHQIAQLEVMTSIALRTLPTMCRVNVPLYRTTTSVPFGVGAG
VGAY.

LMW-D2 924 bp

ATGAAGACCTTCCTCGTCTTTGCCCTCCTCGCCGTTGCGGCGACAAGTGCAATTGCGCAGATGGAGACTA
GATGCATCCCTGGTTTGGAGAGACCATGGCAGCAGCAACCATTACCACCACAACAGACATTTCCACAACA
ACCACTATTTTCACAACAACAACAACAACAACTATTTCCTCAACAACCATCATTTTCGCAGCAACAACCA
CCATTTTGGCAGCAACAACCACCATTTTCTCAGCAACAACCAATTCTACCACAGCAACCACCATTTTCGC
AGCAACAACAACTAGTTCTACCGCAACAACCACCATTTTCACAGCAACAACAACCAGTTTTACCTCCACA
ACAATCACCTTTTCCACAACAACAACAACAACACCAACAGCTGGTGCAACAACAAATCCCTGTTGTTCAG
CCATCCATTTTGCAGCAGCTAAACCCATGCAAGGTATTCCTCCAGCAGCAGTGCAGCCCTGTGGCAATGC
CACAACGTCTTGCTAGGTCGCAAATGTTGCAGCAGAGCAGTTGCCATGTGATGCAACAACAATGTTGCCA
GCAGTTGCCGCAAATCCCCCAGCAATCCCGCTATGAGGCAATCCGTGCTATCATCTACTCCATCATCCTG
CAAGAACAACAACAGGTTCAGGGTTCCATCCAATCTCAGCAGCAGCAACCCCAACAGTTGGGCCAATGTG
TTTCCCAACCCCAACAGCAGTCGCAGCAGCAACTCGGGCAACAACCTCAACAACAACAATTGGCACAGGG
TACCTTTTTGCAGCCACACCAGATAGCTCAGCTTGAGGTGATGACTTCCATTGCGCTCCGTATCCTGCCA
ACGATGTGCAGTGTTAATGTGCCGTTGTACAGAACCACCACTAGTGTGCCATTCGGCGTTGGCACCGGAG
TTGGTGCCTACTGA

LMW-D2 308 aa

MKTFLVFALLAVAATSAIAQMETRCIPGLERPWQQQPLPPQQTFPQQPLFSQQQQQQLFPQQPSFSQQQP
PFWQQQPPFSQQQPILPQQPPFSQQQQLVLPQQPPFSQQQQPVLPPQQSPFPQQQQQHQQLVQQQIPVVQ
PSILQQLNPCKVFLQQQCSPVAMPQRLARSQMLQQSSCHVMQQQCCQQLPQIPQQSRYEAIRAIIYSIIL
QEQQQVQGSIQSQQQQPQQLGQCVSQPQQQSQQQLGQQPQQQQLAQGTFLQPHQIAQLEVMTSIALRILP
TMCSVNVPLYRTTTSVPFGVGTGVGAY.

LMW-D3 897 bp

ATGAAGACCTTCCTCGTCTTTGCCCTTCTAGCCGTTGTGGCGACATCTGCCATTGCACAGATGGAGACTA
GCTGCATCCCTGGTTTGGAGAGACCATGGCAGCAACAACCATTACAACAAAAAGAGACATTTCCACAACA
ACCACCATCTTCACAACAACAACAACCATTTCCTCAACAACCACCATTTTTGCAGCAACAACCTTCATTT
TCGCAGCAACCACTATTTTCACAGAAACAACAACCAGTTCTACCACAACAACCAGCATTTTCGCAGCAAC

AACAAACAGTTCTACCACAACAACCAGCATTTTCGCAGCAACAACACCAACAGCTTCTGCAACAACAAAT
CCCTATTGTTCATCCATCCATTTTGCAGCAGCTAAACCCGTGCAAGGTATTCCTCCAGCAGCAGTGTAGC
CCTGTGGCAATGCCACAACATCTTGCTAGGTCGCAGATGTGGCAGCAGAGCAGTTGCAATGTGATGCAGC
AACAATGTTGCCAACAATTGCCACGAATCCCCGAACAATCCCGCTATGAGGCAATCCGTGCTATCATCTT
CTCCATCATCCTACAAGAACAACAACAGGGTTTTGTCCAACCTCAGCAGCAACAACCCCAACAGTCGGTT
CAAGGTGTCTACCAACCCCAACAGCAGTCGCAGCAGCAGCTCGGACAATGTTCTTTCCAACAACCTCAAC
AACAACTGGGTCAACAACCTCAACAACAACAGGTACAAAAGGGTACCTTTTTGCAGCCACACCAGATAGC
TCGCCTTGAGGTGATGACTTCCATTGCACTCCGTACCCTGCCAACGATGTGCAGTGTCAATGTGCCGTTG
TACAGCTCCATCACTAGTGCGCCATTAGGCGTTGGCAGCCGAGTTGGTGCCTACTGA

LMW-D3 299 aa

MKTFLVFALLAVVATSAIAQMETSCIPGLERPWQQQPLQQKETFPQQPPSSQQQQPFPQQPPFLQQQPSF
SQQPLFSQKQQPVLPQQPAFSQQQQTVLPQQPAFSQQQHQQLLQQQIPIVHPSILQQLNPCKVFLQQQCS
PVAMPQHLARSQMWQQSSCNVMQQQCCQQLPRIPEQSRYEAIRAIIFSIILQEQQQGFVQPQQQQPQQSV
QGVYQPQQQSQQQLGQCSFQQPQQQLGQQPQQQQVQKGTFLQPHQIARLEVMTSIALRTLPTMCSVNVPL
YSSITSAPLGVGSRVGAY.

LMW-D4* 900 bp

ATGAAGACCTTCCTCGTCTTTGCCCTTCTAGCCGTTGTGGCGACATGTGCCATTGCGCAGATGGAGACTA
GGTGCATCCCTGGTTTGGAGAGACCATGGCAGCAACAACCATTACAACCACAACAGATATTTCCACAACA
ACCACCATTTTCACAACAACAACAACAACAACCATTTCCTCAACAACCACCATTTTTAGCACCAACCATC
ATTTTCGCAGCAACTACTATTTTCACAACAACAACGACAACCACTTCTACCGCAACAACCAGCATTTTCG
TAGCAACAACAAACAGTTCTACCGCAACAACCAGCATTTTCTCAACAACAACACCAACAACTTCTGCACC
AACAAATCCCTATTGTTCATCCATCCGTTTTGCAGCAGCTAAACCCATGCAAGGTATTCCTCCAGCAGCA
ATGTAGCCCTGTGGCAATGCCACAACGTCTTGCTAGGTCGCAGATGTGGCAGCAGAGCAGTTGCCATGTG
ATGCGGCAACAATGTTGCCAACAATTGCCGCGAATCCCTGAACAATCCCGCTATGTGGCAATCCGTGCTA
TCATCTTGTCCATCATCCTGCAAGAACAACAATAGGGTTTTGTCCAACCTCAGCAGCAACAACCCCAACA
GTCGGTTCAAGGTGTCTACCAACCCCAACAGCAGTCGCAGCAGCAGCTCGGACAATGTTCTTTCCAACAA
CCTCAACAACAACTGGGTCAACAGCCTCAACAACAACATGTACAATAGGGTACTTGCAGCCACACCAGAT
AGCTCGCCTCGAGGTGATGACTTCCATTGCCCTCAGTACCCTGCCAACGATGTGCAGTGTTAATGTGCCG
TTGTACAGCTCCATCACTAGTGTGCCATTCGGTGTTGGCAGCGGAGTTGGTGCCTACTGA

LMW-D5* 1113 bp

ATGAAGACCTTCCTCATCTTTGCCCTCCTCGCCGTTGCGGCGACAAGTGCCATTGCACAAATGGAGACTA
GCCACATCCCTGGCTTGGAGAAACCATCGCAACAACAACCATTACCACTACAACAAATATTATGGTACCA
CCAACAGCAACCCATCCAACAACAACCACAACCATTTCCACAACAGCCACCATGTTCACAGCAACAACAA
CCACCATTATCGCAGCAACAACAACCACCATTTTCACAGTAACAACCACCATTCTCGCAGCAAGAACTAC
CAGTTCTACCGCAACAACCACCATTTTCGCAGCAACAACAACCATTCCCGCAGCAACAACAACCACTTCT
ACCGCAACAACCCCCATTTTCACAACAACGACCACCATTTTCTCAGCAGCAGCAACAACCAGTTCTACCG
CAACAACCACCATTTACGCAACAACAGCAACAACAACAACAACAACAACAACAACAACAACAACAACAAC
AACAACCAATTCTACCGCAACAACCACCTTTTTCGCAACACCAACAACCGGTTCTGCCGCAACAACAAAT
ACCATATGTTCAGCCATCTATCTTGCAGCAGCTAAACCCATGCAAGGTATTCCTCCAGCAGCAATGCAGC
CCTGTGGCAATGCCACAAAGTCTTGCTAGGTCACAAATGTTGTGGCAGAGCAGTTGCCATGTGATGCAGC
AACAATGTTGCCAGCAGCTGCCGCGAATCCCCGAACAATCACGCTATGATGCAATCCGTGCCATCATCTA
CTCGATCGTCCTACAAGAACAACAACATGGTCAGGGTTTCAACCAACCTCATCAGCAACAACCCCAACAG
TCGGTCCAAGGTGTCTCCCAACCCCAACAACAACAGAAGCAGCTCGGACAGTGTTTTTTCCAACGACCTC
AACAACAACAACTGGGTCAATGGCCTCAACAACAACAGGTACCACAGGGTACCTTGTTGCAGCCACACCA
AATAGCTCAACTTGAGTTGATGACTTCCATTGCACTCCGTACCCTGCCAATGATGTGCAGTGTCAACGTG
CCGGTGTACGGCACCACCACTAGTGTGCCATTCGGCGTTGGCACCCAAGTTGGTGCCTACTGA

LMW-D6 1053 bp

ATGAAGACCTTCCTCATCTTTGCTCTCCTTGCCATTGCGGCGACAAGTGCCATTGCACAAATGGAGACTA
GCCGCGTCCCTGGTTTGGAGAAACCATGGCAGCAACAACCATTACCACCACAACAACAACCACCATGTTC
ACAGCAACAACAACCATTTCCACAGCAACAACAACCAATTATTATACTGCAACAATCACCATTTTCGCAG
CAACAACAACCAGTTCTGCCGCAACAGCAACCAGTTATTATACTGCAACAACCACCATTTTCGCAGCAAC
AACAACCAGTTCTACCACAACAACCACCATTTTCACAACAACAACAACAACAACAACAACAACAACCACC
ATTTTCGCAGCAACAACAACCAGTTCTACCACAACAACCACCATTTTCACAACAACAACAACCACCATTT
TCGCAGCAGCAACAACCATCTTCACAACAACCACCTTTTCCACAACAACACCAACAGTTTCCACAACAAC
AAATCCCTGTTGTTCAACCATCCGTTTTGCAGCAGCTAAACCCATGCAAGGTGTTCCTCCAACAGCAGTG
TAGCCATGTGGCAATGTCGCAACGTCTTGCTAGGTCACAAATGTGGCAACAGAGTAGTTGCCATGTGATG
CAACAACAATGTTGCCAACAGCTGCCGCAAATCCCCGAACAATCCCGCTCTGAGGCAATCCGTGCCATCG
TCTACTCCATCATCCTGCAAGAACAACAACAGGGTTTTGTCCAACCTCAGCAGCAACAACCCCAACAGTC
GGGCCAAGGTGTCTCCCAACACCAACAGCAGTCGCAGCAGCAGCAGCAACTCGGACAGTGTTCTTTCCAA
CAACCTCAACAACTACAACAATTGGGTCAGCAGCCTCAACAACAACAGATACCACAGGGTATATTCTTGC
AGCCACACCAGATATCTCAACTTGAGGTGATGACTTCCATTGCACTCCGTACCTTGCCAACGATGTGCGG
TGTCAACGTGCCGTTGTACAGCTCGACCACTATTATGCCATTCAGCATTGGCACTGGAGTTGGTGGCTAC
TGA

LMW-D6 351 aa

MKTFLIFALLAIAATSAIAQMETSRVPGLEKPWQQQPLPPQQQPPCSQQQQPFPQQQQPIIILQQSPFSQ
QQQPVLPQQQPVIILQQPPFSQQQQPVLPQQPPFSQQQQQQQQQPPFSQQQQPVLPQQPPFSQQQQPPF
SQQQQPSSQQPPFPQQHQQFPQQQIPVVQPSVLQQLNPCKVFLQQQCSHVAMSQRLARSQMWQQSSCHVM
QQQCCQQLPQIPEQSRSEAIRAIVYSIILQEQQQGFVQPQQQQPQQSGQGVSQHQQQSQQQQQLGQCSFQ
QPQQLQQLGQQPQQQQIPQGIFLQPHQISQLEVMTSIALRTLPTMCGVNVPLYSSTTIMPFSIGTGVGGY
.

LMW-D7 912 bp

ATGAAGACCTTCCTCGTCTTTGCCCTCATCGCCGTTGTGGCGACAAGTGCCATTGCACAGATGGAGACTA
GCTGCATCTCTGGTTTGGAGAGACCATGGCAGCAGCAACCATTACCACCACAACAGTCATTTTCACAACA
ACCACCATTTTCACAACAACAACAACAACCATTACCTCAACAACCATCATTTTCGCAGCAACAACCACCA
TTTTCGCAGCAACAACCAATTCTATCGCAGCAACCACCATTTTCACAGCAACAACAACCAGTTCTACCGC
AACAATCACCATTTTCGCAGCAACAACAACTAGTTTTACCTCCACAACAACAACAACAACAACTTGTGCA
ACAGCAAATCCCTATTGTTCAGCCATCCGTTTTGCAGCAGCTAAACCCATGCAAAGTATTCCTCCAGCAG
CAGTGCAGCCCTGTAGCAATGCCACAACGTCTTGCTAGGTCACAGATGTGGCAGCAGAGCAGTTGCCATG
TGATGCAACAACAATGTTGCCAGCAATTGCAGCAAATCCCCGAACAATCCCGCTATGAAGCAATCCGTGC
CATCATCTACTCCATCATCCTGCAAGAACAACAACAGGGCTTTGTCCAACCTCAGCAGCAACAACCCCAA
CAGTCAGGTCAAGGTGTCTCCCAATCCCAACAGCAGTCGCAGCAGCAGCTCGGACAATGTTCTTTCCAAC
AACCTCAACAGCAACTGGGTCAACAGCCTCAACAACAACAACAACAGGTACTACAGGGTACCTTTTTGCA
GCCACACCAGATAGCTCACCTTGAGGCGGTGACTTCCATTGCACTCCGTACCTTGCCAACGATGTGCAGT
GTCAATGTGCCGTTGTACAGCGCCACCACCAGTGTGCCATTCGGCGTTGGCACCGGAGTTGGTGCCTACT
GA

LMW-D7 304 aa

MKTFLVFALIAVVATSAIAQMETSCISGLERPWQQQPLPPQQSFSQQPPFSQQQQQPLPQQPSFSQQQPP
FSQQQPILSQQPPFSQQQQPVLPQQSPFSQQQQLVLPPQQQQQQLVQQQIPIVQPSVLQQLNPCKVFLQQ
QCSPVAMPQRLARSQMWQQSSCHVMQQQCCQQLQQIPEQSRYEAIRAIIYSIILQEQQQGFVQPQQQQPQ
QSGQGVSQSQQQSQQQLGQCSFQQPQQQLGQQPQQQQQQVLQGTFLQPHQIAHLEAVTSIALRTLPTMCS
VNVPLYSATTSVPFGVGTGVGAY.

LMW-D8 1098 bp

ATGAAGACCTTCCTCATCTTTGCCCTCCTTGCCGTTGCAGCGACAAGTGCCATTGCACAAATGGAGACTA
GCCACATCCCTGGCTTGGAGAAACCATCGCAACAACAACCATTACCACTACAACAAATATTATGGTACCA
CCAACAGCAACCGATCCAACAACAACCACAACCATTTCCACAACAGCCACCATGTTCACAGCAACAACAA
CCACCATTATCGCAGCAACAACAACCACCATTTTCACAGCAACAACCACCATTCTCGCAGCAAGAACTAC
CAATTCTACCGCAACAACCACCATTTTCGCAGCAACAACAACCACAATTTTCGCAGCAACAACAACCATT
CCCGCAGCAACAACAACCACTTCTACTGCAACAACCCCCATTTTCACAACAACGACCACCATTTTCTCAG
CAGCAGCAACAACCAGTTCTACCGCAACAACCACCATTTTCGCAACAGCAACAACAACAACCAATTCTAC
CGCAACAACCACCTTTTTCGCAACACCAACAACCGGTTCTTCCGCAACAACAAATACCATATGTTCAGCC
ATCTATCTTGCAGCAGCTAAACCCATGCAAGGTATTCCTCCAGCAGCAATGCAGCCCTGTGGCAATGCCA
CAAAGTCTTGCTAGGTCGCAAATGTTGTGGCAGAGCAGTTGCCATGTGATGCAGCAACAATGTTGCCAGC
AGCTGCCGCGAATCCCCGAACAATCACGCTATGATGCAATCCGTGCCATCATCTACTCGATCGTCCTACA
AGAACAACAACATGGTCAGGGTTTCAACCAACCTCAGCAGCAACAACCCCAACAGTCGGTCCAAGGTGTC
TCCCAACCCCAACAACAACAGAAGCAGCTCGGACAGTGTTCTTTCCAACGACCTCAACAACAACAACTGG
GTCAATGGCCTCAACAACAACAGGTACCACAGGGTACCTTGTTGCAGCCACACCAAATAGCTCAACTTGA
GTTGATGACTTCCATTGCACTCCGTACCCTGCCAATGATGTGCAGTGTCAACGTGCCGGTGTACGGCACC
ACCACTAGTGTGCCATTCGGCGTTGGCACCCAAGTTGGTGCCTACTGA

LMW-D8 366 aa

MKTFLIFALLAVAATSAIAQMETSHIPGLEKPSQQQPLPLQQILWYHQQQPIQQQPQPFPQQPPCSQQQQ
PPLSQQQQPPFSQQQPPFSQQELPILPQQPPFSQQQQPQFSQQQQPFPQQQQPLLLQQPPFSQQRPPFSQ
QQQQPVLPQQPPFSQQQQQQPILPQQPPFSQHQQPVLPQQQIPYVQPSILQQLNPCKVFLQQQCSPVAMP
QSLARSQMLWQSSCHVMQQQCCQQLPRIPEQSRYDAIRAIIYSIVLQEQQHGQGFNQPQQQQPQQSVQGV
SQPQQQQKQLGQCSFQRPQQQQLGQWPQQQQVPQGTLLQPHQIAQLELMTSIALRTLPMMCSVNVPVYGT
TTSVPFGVGTQVGAY.
