# Supplementary material for: Gene Duplication and Evolution Dynamics in the Homeologous Regions Harboring Multiple Prolamin and Resistance Gene Families in Hexaploid Wheat
Source: Front Plant Sci. 2018 May 23;9:673. doi: 10.3389/fpls.2018.00673 (PMC5974169; doi:10.3389/fpls.2018.00673)
Supplement: Supplementary file 7 [file Image_3.pdf]

Figure S3

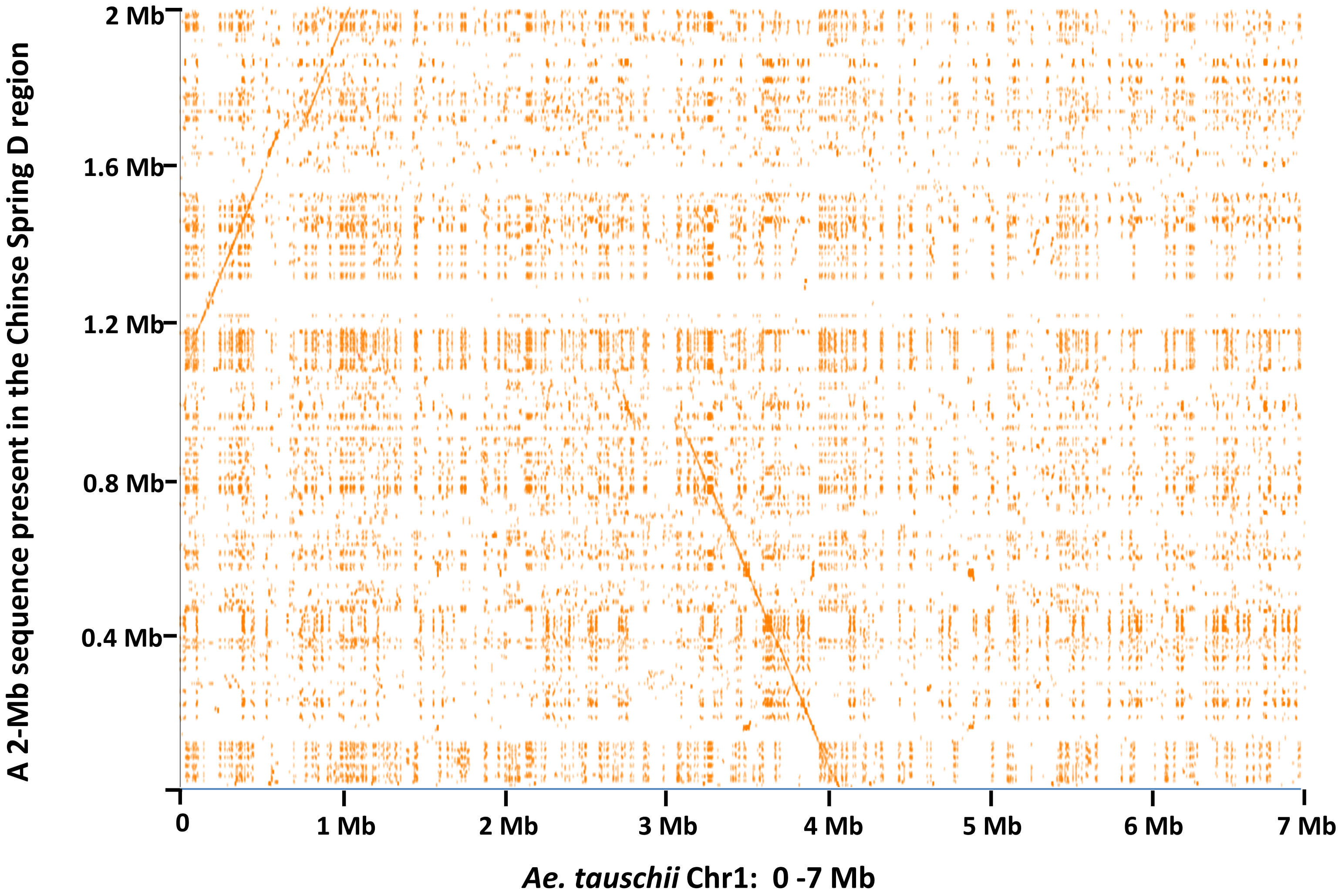

Figure S3. Dotplot analysis of the 2-Mb regions in the Chinese Spring D region against the first 7-Mb sequence of *Ae. tauschii* chromosome 1
