# Supplementary material for: Gene Duplication and Evolution Dynamics in the Homeologous Regions Harboring Multiple Prolamin and Resistance Gene Families in Hexaploid Wheat
Source: Front Plant Sci. 2018 May 23;9:673. doi: 10.3389/fpls.2018.00673 (PMC5974169; doi:10.3389/fpls.2018.00673)
Supplement: Supplementary file 8 [file Image_4.pdf]

Figure S4

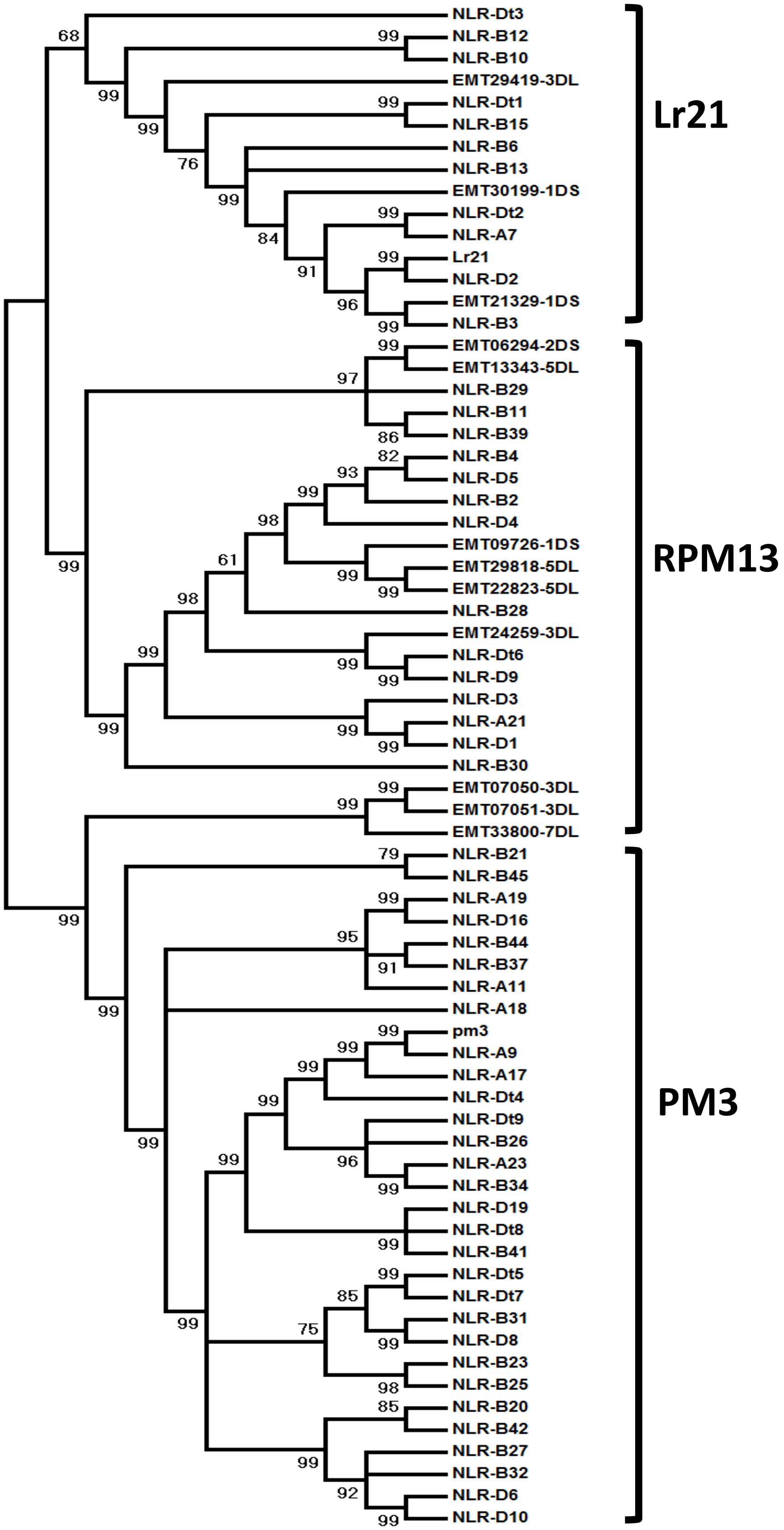

Figure S4. Phylogeny of NLR genes in the orthologous regions of the A, B and D genomes of Chinese Spring and D genome from *Ae. tauschii*.
The coding sequences of *NLR* genes based on the annotation were used for constructing the phylogenetic tree using MEGA7 with the Neighbor-Joining method. The confidence probability (multiplied by 100) that the interior branch length is greater than 0, as estimated using the bootstrap test (1000 replicates) is shown next to the branches. Functional LR21 and Pm3 resistance genes, as well as those annotated in *Ae. tauschii* (Dong *et al.*, 2016) were included in the analysis.
