# Supplementary material for: Gene Duplication and Evolution Dynamics in the Homeologous Regions Harboring Multiple Prolamin and Resistance Gene Families in Hexaploid Wheat
Source: Front Plant Sci. 2018 May 23;9:673. doi: 10.3389/fpls.2018.00673 (PMC5974169; doi:10.3389/fpls.2018.00673)
Supplement: Supplementary file 9 [file Image_5.pdf]

Figure S5

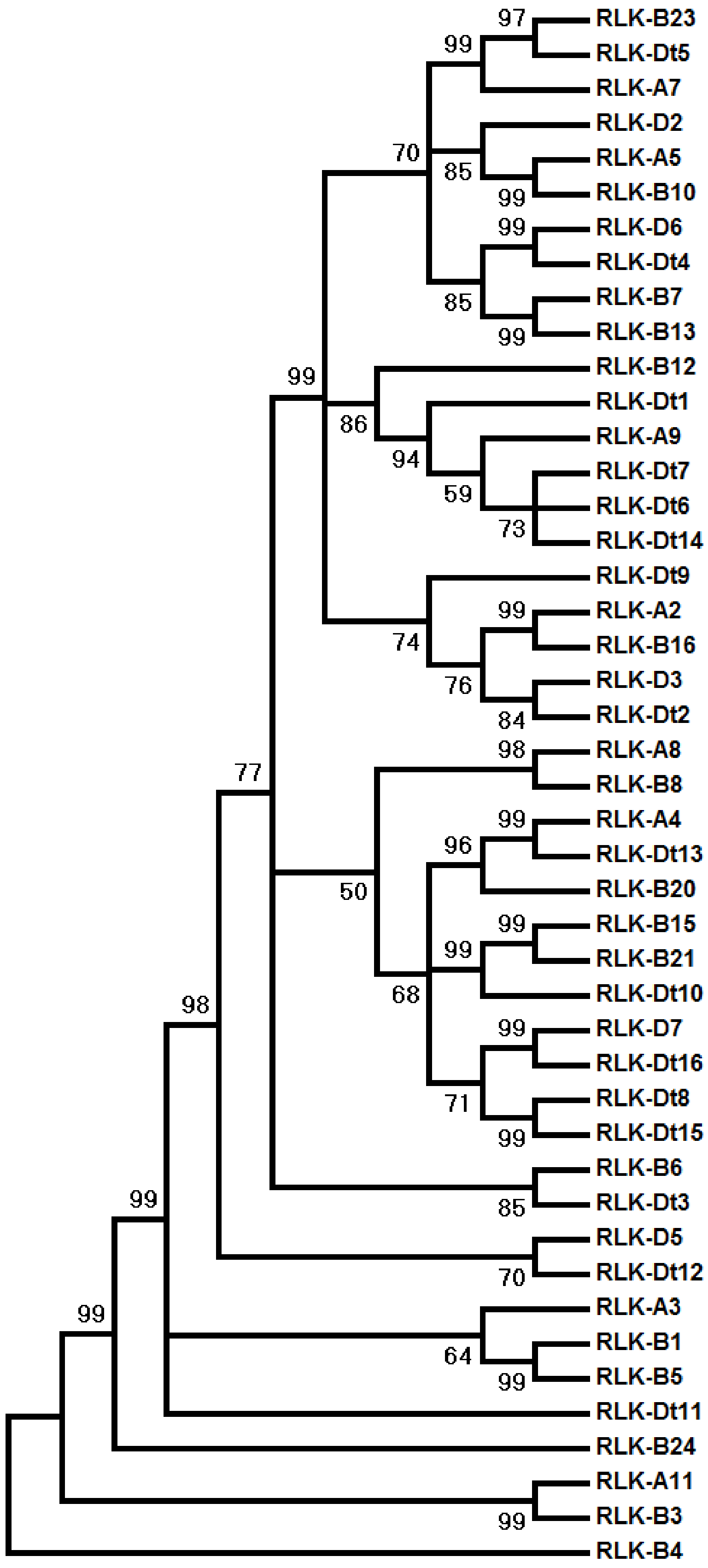

Figure S5. Phylogeny of RLK genes in the orthologous regions of the A, B and D genomes of Chinese Spring and D genome from *Ae. tauschii*. The coding sequences of RLK genes based on the annotation were used for constructing the phylogenetic tree using MEGA7 with the Neighbor-Joining method . The confidence probability (multiplied by 100) that the interior branch length is greater than 0, as estimated using the bootstrap test (1000 replicates) is shown next to the branches.
